# Advancing Energy Development with MBene: Chemical Mechanism, AI, and Applications in Energy Storage and Harvesting

**DOI:** 10.1007/s40820-025-01941-8

**Published:** 2026-01-04

**Authors:** Jai Kumar, Nadeem Hussain Solangi, Rana R. Neiber, Fangyuan Bai, Victor Charles, Pengfei Zhai, Zhuanpei Wang, Xiaowei Yang

**Affiliations:** 1 School of Energy Science and Technology, Henan University, Zhengzhou, 450046 People’s Republic of China; 2 State Key Laboratory of Organic-Inorganic Composites Beijing Key Laboratory of Electrochemical Process and Technology for Materials, Beijing University of Chemical Technology, Beijing, 100029 People’s Republic of China; 3 Beijing Key Laboratory of Ionic Liquids Clean Process, CAS Key Laboratory of Green Process and Engineering, Institute of Process Engineering, Chinese Academy of Sciences, Beijing, 100190 People’s Republic of China; 4 Department of Nanoengineering, Joint School of Nanoscience and Nanoengineering, North Carolina A & T State University, Greensboro, NC 27401 USA; 5 School of Chemistry and Chemical Engineering, Shanghai Jiao Tong University, Shanghai, 200240 People’s Republic of China; 6 Longzihu New Energy Laboratory, Zhengzhou Institute of Emerging Industrial Technology, Henan University, Zhengzhou, 450000 People’s Republic of China

**Keywords:** MBene, MXene, Energy storage, CO_2_ reduction, Nitrogen reduction reactions, Artificial intelligence

## Abstract

MXene derivatives are notable two-dimensional nanomaterials with numerous prospective applications in the domains of energy development. MXene derivative, MBene, diversifies its focus on energy storage and harvesting due to its exceptional electrical conductivity, structural flexibility, and mechanical properties. This comprehensive review describes the sandwich-like structure of the synthesized MBene, derived from its multilayered parent material and its distinct chemical framework to date. The fields of focus encompass the investigation of novel MBenes, the study of phase-changing mechanisms, and the examination of hex-MBenes, ortho-MBenes, tetra-MBenes, tri-MBenes, and MXenes with identical transition metal components. A critical analysis is also provided on the electrochemical mechanism and performance of MBene in energy storage (Li/Na/Mg/Ca/Li–S batteries and supercapacitors), as well as conversion and harvesting (CO_2_ reduction, and nitrogen reduction reactions). The persistent difficulties associated with conducting experimental synthesis and establishing artificial intelligence-based forecasts are extensively deliberated alongside the potential and forthcoming prospects of MBenes. This review provides a single platform for an overview of the MBene’s potential in energy storage and harvesting.

## Highlights

- Revealing the synergistic potential of MBene as an advanced material.
- Comprehensive study into MBene chemistry and electrochemical efficacy.
- The potential research for batteries, supercapacitors, CO_2_ reduction, and nitrogen reduction reactions is unveiled.
- AI-driven predictions and limitations in experimental synthesis are addressed comprehensively.

## Graphical abstract

**Figure:**
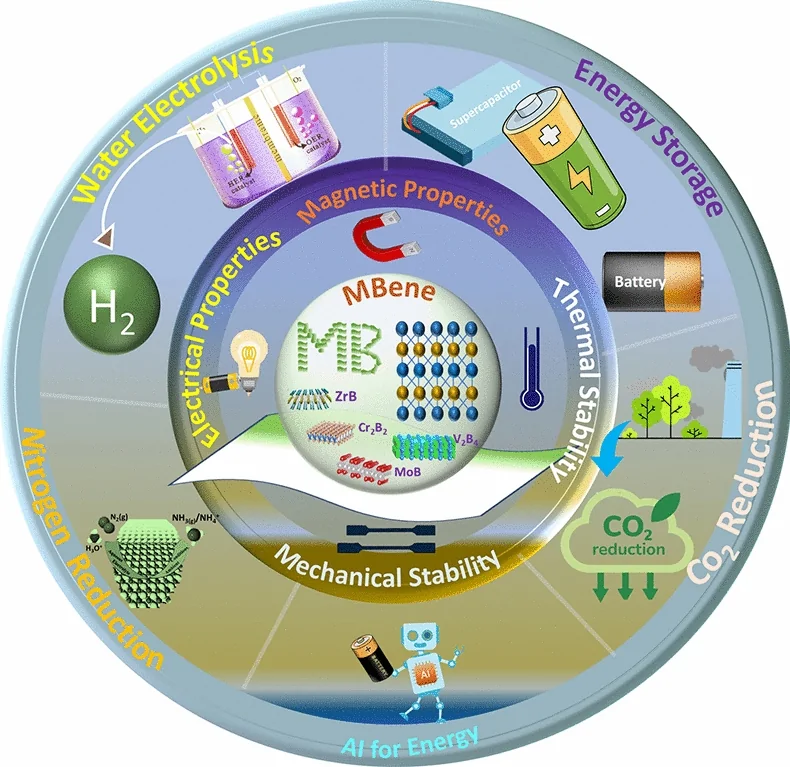

## Introduction

The enhancement of industrialization and commercialization is responsible for the high demand for energy storage, conversion, and transformation. The massive utilization of fossil fuels to meet energy requirements limits their resources, increasing CO_2_ emissions, which threaten human beings [1]. To address these challenges, particularly in developing nations, generating and extensively utilizing sustainable energy sources, including solar, geothermal, wind, and tidal power, is essential [2]. Batteries and supercapacitors (SCs) are the primary sources of energy storage and conversion. The existing battery sources exhibited inadequate electrochemical performance. The chemical composition, structure, and qualities of the electrode material significantly influence the efficacy of charge storage devices. Thus, producing a remarkable material with exceptional electrochemical performance for electrode applications is essential.

**Fig. 1:**
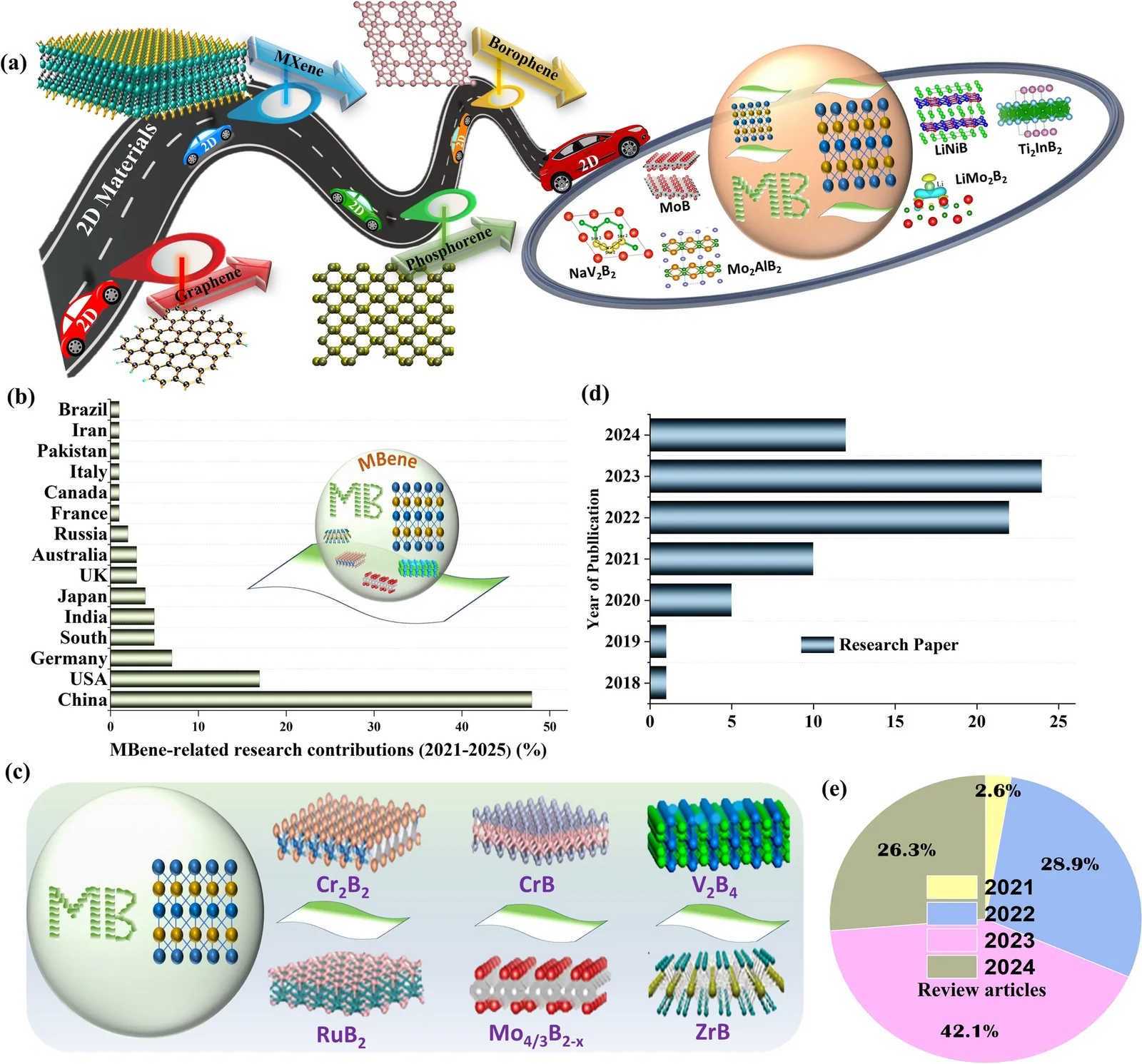
**a–b** Different types of 2D materials and the emergence of MBene in different regions. **c** different derivatives of MBene. **d–e** Statistical analysis of the MBene number of years versus publication from its discovery. (Note: The data were collected directly from science using the keywords MBene-based electrode material for energy storage and harvesting)

The present era is a two-dimensional (2D) material in energy storage and conversion because of its unique properties, including high specific surface area, many active sites, and outstanding thermal, chemical, and physical properties, such as MXene, graphene, and borophene, as illustrated in Fig. 1a [3–5]. MXenes were discovered by Gogotsi and Barsoum from Drexel University. Similar to the MXene structure, MBene has been expanded in several different regions, as shown in Fig. 1b. The pure MXene (M_n+1_X_n_) is an inorganic chemical created by selectively removing the A layer from its parent material, MAX. By selectively etching in HF or in situ HF-generating solutions, the created pure material contained good functional terminations, such as –O, –F, and –OH. MXene generally consists of transition metal carbides, nitrides, and carbonitrides, with the chemical formula M_n+1_X_n_T_*x*_ [6, 7]. In this equation, M, X, and T represent transition metals, carbon or nitrogen, and surface termination groups, respectively [8]. Since the initial discovery of MXene, almost 100 distinct varieties with varying chemical compositions and structures have been identified both theoretically and empirically and utilized in diverse applications, including environmental science, energy, catalysis, and electronics [9, 10]. The unique properties and performance of MXene increase scientists’ interest in extensively investigating the X phase of MXene. Ade and Hillebrecht opened the curtain on the advanced 2D material MBene in 2015 [11]. In the last decay, scientists eliminated the X phase from MAX by B to synthesize the new unique material MBene. Similarly, the 2D material MXene and MBenes was made by selectively removing the A layer from their parental material, MAB. It is also known as the closest relative of MXene due to the solid similarity between the MAX and MAB parent phases. In the general formula of MBene (M_n_B_2n − 2_), M and B represented the transition metal (e.g., Cr, Mo, W, Fe, Mn) and boron, respectively. In the formula, n is the whole 2, 3, and 4 [10, 12]. After extensive studies, the authors identified 50 MAB phases with varying chemical compositions and morphologies, as illustrated in a few examples in Fig. 1c [10]. MBene, like MXene, is a new material with unique performance due to its distinctive structure, morphology, and excellent charge storage capacity. The properties that make it an ideal candidate for charge storage are good electrical conductivity, mechanical stability, and charge transport mobility [13–15]. The theoretical calculation indicated that the MBene is parallel to the MXene in energy storage and conversion. Recently, it is believed that the MBene is very close to MXene. MBene’s superior theoretical charge storage, harvesting, and stability compared to MXene make it a promising candidate for these sectors. The comparative statical analysis of the MBene number of years vs. publication from its discovery is presented in Fig. 1d, e.

A review comparing MBene-based electrodes to MXene, potentially describing the overall mechanism between the two materials and their advancements, may be helpful, especially regarding energy storage and harvesting [16, 17]. This overview outlines the various MBene kinds currently available, as well as the methods used to synthesize them. Additional information was provided regarding the most popular MBene material for energy storage and conversion applications. Along with the results of their study, they compared MXene with MBene. We concluded by addressing the difficulties and potential future projects involving MBenes. We believe this review would be a great addition to a centralized resource outlining the benefits of MBenes for energy conversion and storage.

### Comparison Between MXene and MBene

**Fig. 2:**
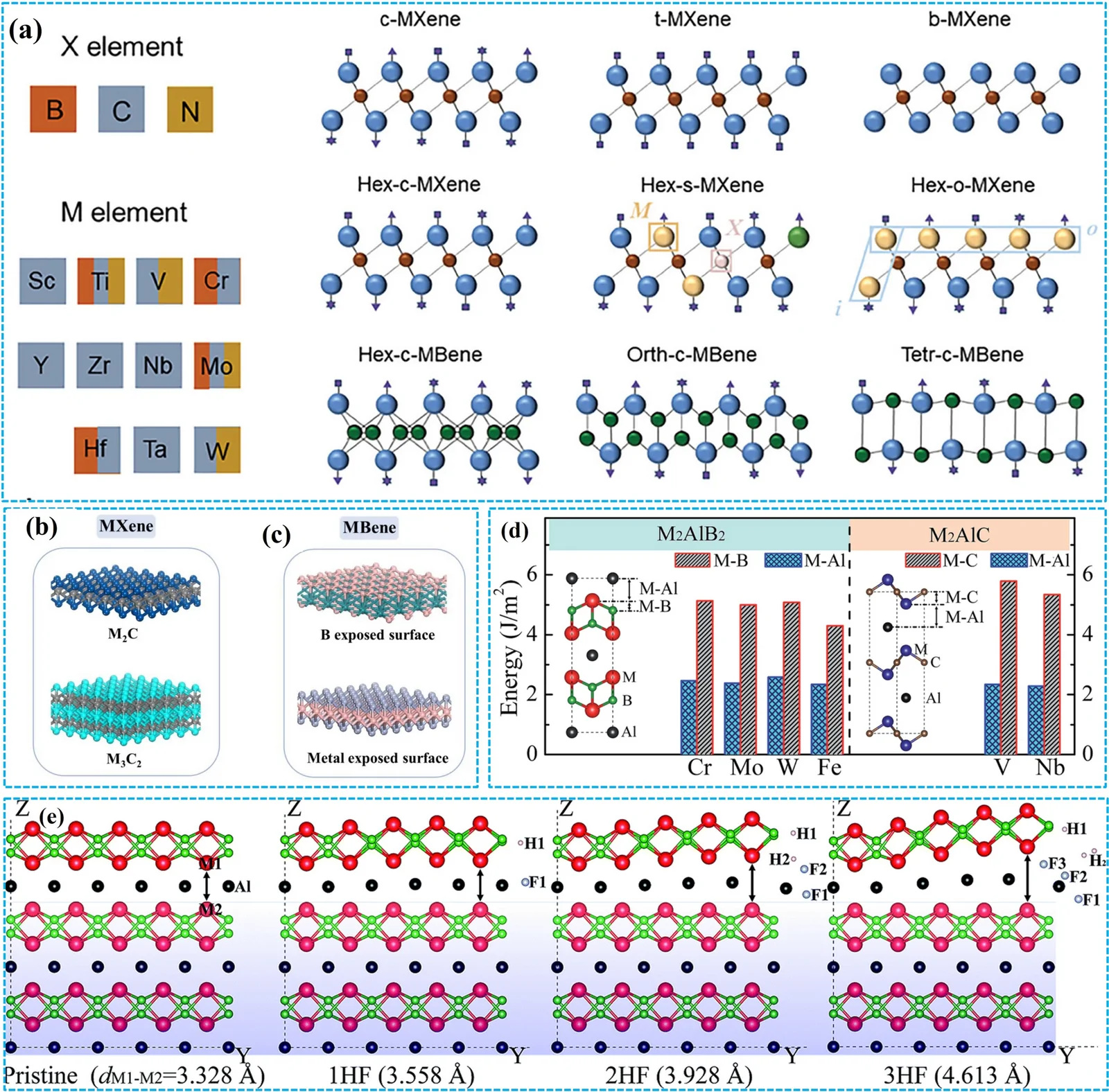
**a** Elemental compositions and structures of MXene and MBene (adapted with permission [4]). Structure of the **b** M_2_C and M_3_C MXene, **c** MBene (adapted with permission from [28]). **c** Difference between the bonding energies of the M_2_AlB_2_ and M_2_AlC represented MAB and MAX phase, **d** where the green, red pink, black, and cyan, balls signify Mo, B, Al, F, and H atoms (adapted with permission [24])

MXene and MBene possess analogous layered architectures consisting of surface and bulk phases; however, MBenes have a greater variety of crystalline forms. Figure 2a illustrates that both materials may be characterized inside a cohesive framework where X equals B, C, or N. Although certain elements such as M_n_ have been synthesized in MAX phases, their matching MXenes have yet to be produced. Historically, 2D borides like MnB₂, MgB₂, and ZrB₂ were categorized as MBenes; however, new research has redefined MBene to encompass 2D transition metal borides particularly originating from MAB phases, characterized by the general formula MnB_2n-2_ (*n* = 2–4) [18]. To conform to the concept of MXene, we revise the general formula of MBene to M_n+1_B_2n_T_*x*_ (*n* = 1–3), considering the unavoidable surface functionalization that occurs during synthesis. This formula encompasses the majority of known MBenes and corresponds with Sun’s revised definition. Based on the elemental competition, MXene and MBene can be differentiated by chemical composition: MXene is an assembly of the transition metal and carbon/nitrogen, and MBene is composed of transition metal and boron. The primary similarity between MBene and MXene lies in their structure, as both 2D materials possess layer-like structures, as presented in Fig. 2b, c. In contrast, M–A bonds are metallic. The interaction power of the ionic bond is always more substantial than the metallic bond, so breaking M–A bonds removes A layer from MAX during the etching process. In contrast, the M–X bonds continue unbroken. The effective closeness between the structural assembly and configuration of the MAX and MAB was experimentally investigated. The nature of the M–B bond is ionic and covalent, but M–A is a weak metallic bond compared to the ionic bond. The M–A bond in MAB has a comparatively high metallic strength compared to the MAX phase. Therefore, eliminating A from the MAX phase is relatively more accessible than from the MAB phase due to the significant difference in electronegativity between the X and B layers. A solid metallic bond between the M–B and M–C layers is highly electronegative compared to that of bromine (3.6), which is higher than that of carbon (2.6). The identical bond behavior of MAB allows the creation of novel 2D transition metal boride. Thus, 2D TMBs are anticipated to be created by splitting the transition metal boride and A or A_2_M layers. The intense binding energies of MXenes and MBene indicate stable architectures. MXenes have been more thoroughly investigated, usually with well-documented binding energies ranging from -3 to -6 eV per formula unit. However, their binding energies are less well documented; MBenes, being more recent, have substantial binding energies, maybe even more than MXenes based on the early study. Generally speaking, the MXene and MBene have binding energy ranging from 3 to − 6 eV and from − 5 eV per formula unit or higher, suggesting strong structures [19–23]. The graphical comparative analysis of the bonding energy between the Mo–Al and Mo–B layers is presented in Fig. 2d, e**.** It can be visualized that the bonding energy of the Mo–B bond is greater than the Mo–Al layer. These findings indicated that the exfoliation of the bulk Mo–B is more complicated than that of the bulk Mo–Al due to the significant difference in their bonding energy. The electron localization function (ELF) provides a meaningful scientific reason why M–B bonding energy is greater than the M–A bonding energy; according to their findings, bonding in M–A is metallic, whereas M–B is covalent, metallic, or ionic [24]. MXenes and MBenes exhibit high binding energies and consequent stability, which suggests promise for various applications. The particular material composition and functionalizing can affect the exact values. Both materials possess different band structures; for example, MXene has a metallic band structure, which allows for flexibility in modification due to the influential surface termination groups on its surface [25, 26]. MBenes exhibit more diverse band structures, ranging from metallic to semiconducting properties, based on the particular metal–boron composition of the molecule [27].

**Fig. 3:**
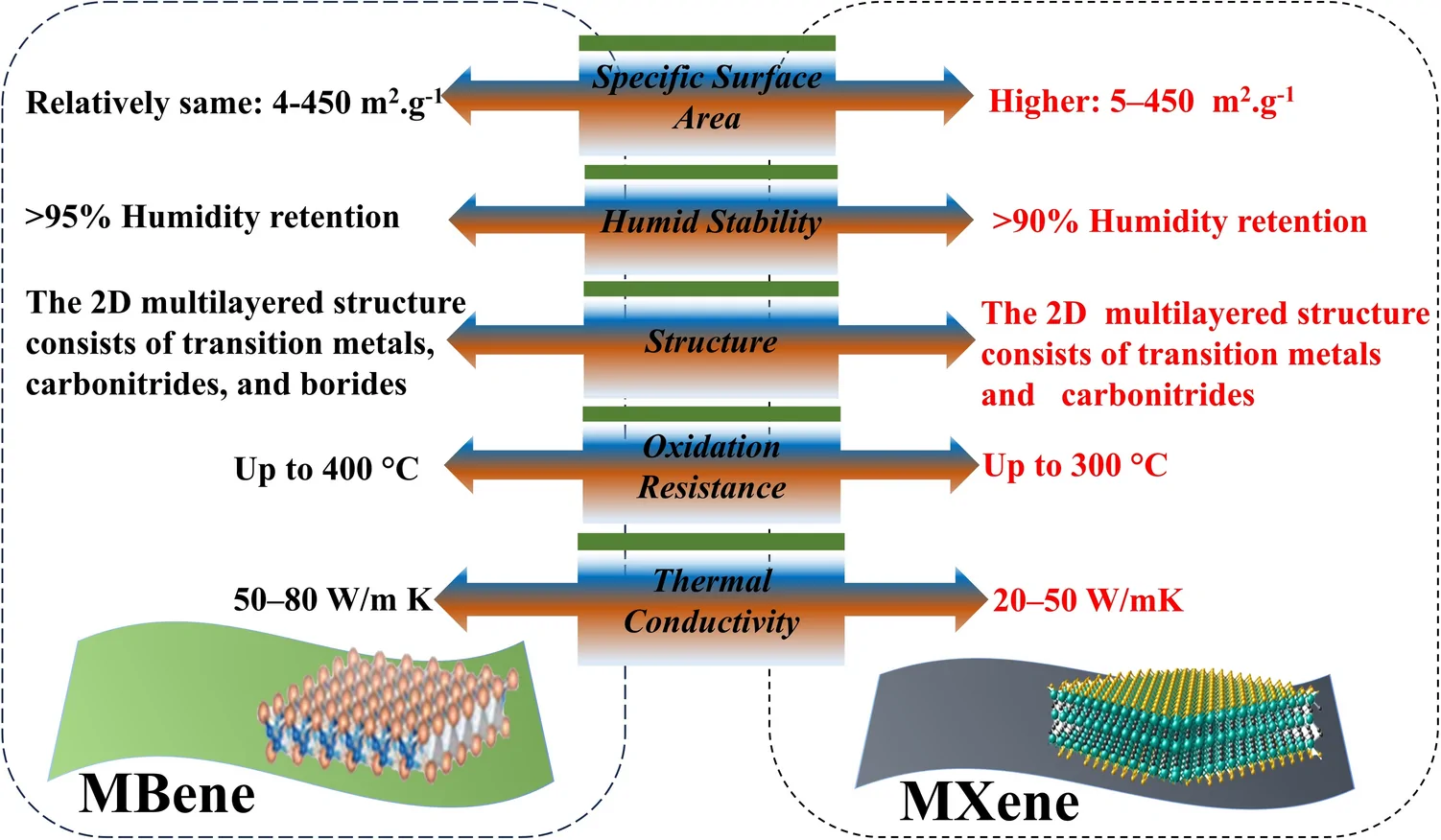
A comparative analysis of the betterment of the other MXene derivative and MBene red and black letters show the characteristics of MXene and MBene, respectively [31]

The MXene possessed -O, -F, and -OH surface functions. Their surface functionalities quickly passivate the surface metal atoms, reducing their adsorption or storage capacity. So, for the adsorption of carbon dioxide (CO_2_) surface functionalities, free MXenes have superiority to adsorbing the CO_2_ over MXene-possessed surface functionalities. The performance of the functionalized free MXene 2D Mo_2_C is higher than the functionalized Mo_2_CT_*x*_ MXene because of the reduction of interlayer distance and the more significant fraction of exposed Mo atoms. The stability of the MXene was also precious by the termination of the MXene; the same trend was observed in the stability of MXene. Zhou et al. [29] and Persson et al. [30] discuss extensively the impact of the surface functionalities on the catalytic activity of the MXene. Compared to MXene, MBenes can be stabilized without surface functionalities, providing a platform for the ideal catalytic degradation of CO_2_. Wang et al. [14] compared the mechanical and thermal stability of the MBene and MXene. Their critical analysis indicated that the synthesized Ti_2_B_2_ is stronger than the MXene. They also did not observe any change in the structure of the MBene at elevated temperatures up to 1773 K as compared to the MXene. MBene’s multilayer hexagonal shape and close-packed atomic stack pattern make it superior to MXene. It also exhibited a strong Ti–B covalent bond across (001), metallic electrical conductivity, and a ceramic-like low heat capacity. Figure 3 reveals that MBene and MXene exhibit comparable structural and physicochemical characteristics; however, MBene shows marginal enhancements in humidity stability and oxidation resistance, which may provide benefits in specific applications.

## Highlights of This Review

Recent review papers have focused on the emerging domain of MBene materials, highlighting their potential as two-dimensional transition metal borides with significant implications for energy storage and catalysis. A notable study is the comparative analysis by Chen et al., which thoroughly examines the structural, electrical, and electrochemical characteristics of MBene about MXene [4]. This study highlights the morphological and surface chemical similarities while emphasizing the greater structural variety and stability concerns of MBenes. Moreover, extensive analyses of MXene materials, including those by Anasori et al., offer profound insights into MXene synthesis, surface terminations, and diverse applications [32]. Incorporating these viewpoints enhances the contextualization of MBene growth and elucidates the rationale for investigating MBene within the broader 2D material framework.

Despite the increasing interest in MBene materials in recent years, most review publications predominantly focus on specific facets, such as synthesis methods, crystal structure, or particular application areas. Zhang et al. [33] focus on the electrocatalytic properties of MBene, whereas Hayat et al. [16] provide comprehensive analyses of structural attributes and synthesis techniques. Other recent publications adopt a tightly focused methodology that emphasizes discrete performance assessments or particular functional results [34].

Nonetheless, despite these significant contributions, the field remains lacking a thorough review that synthesizes MBene’s varied applications, investigates the fundamental mechanisms influencing performance, and examines emerging research trajectories, especially those about artifical intellengence (AI)-assisted material discovery and optimization, which constitute a swiftly advancing and consequential domain. MBene, although theoretically related to MXene, is a unique category of 2D materials characterized by greater chemical and structural variability, necessitating specialized examination. This study fulfills an unmet need by providing a comprehensive and relevant overview of MBene materials that extend beyond synthesis and structure. We methodically investigate MBene’s multifunctional capabilities in energy storage and harvesting while rigorously assessing performance-limiting issues and prospective prospects. Additionally, we integrate data-driven and computational methodologies, an aspect that has been hitherto underrepresented in MBene literature, which will define our study and equip it to inform both foundational research and application-oriented advancements.

## Chemistry of MBenes

**Fig. 4:**
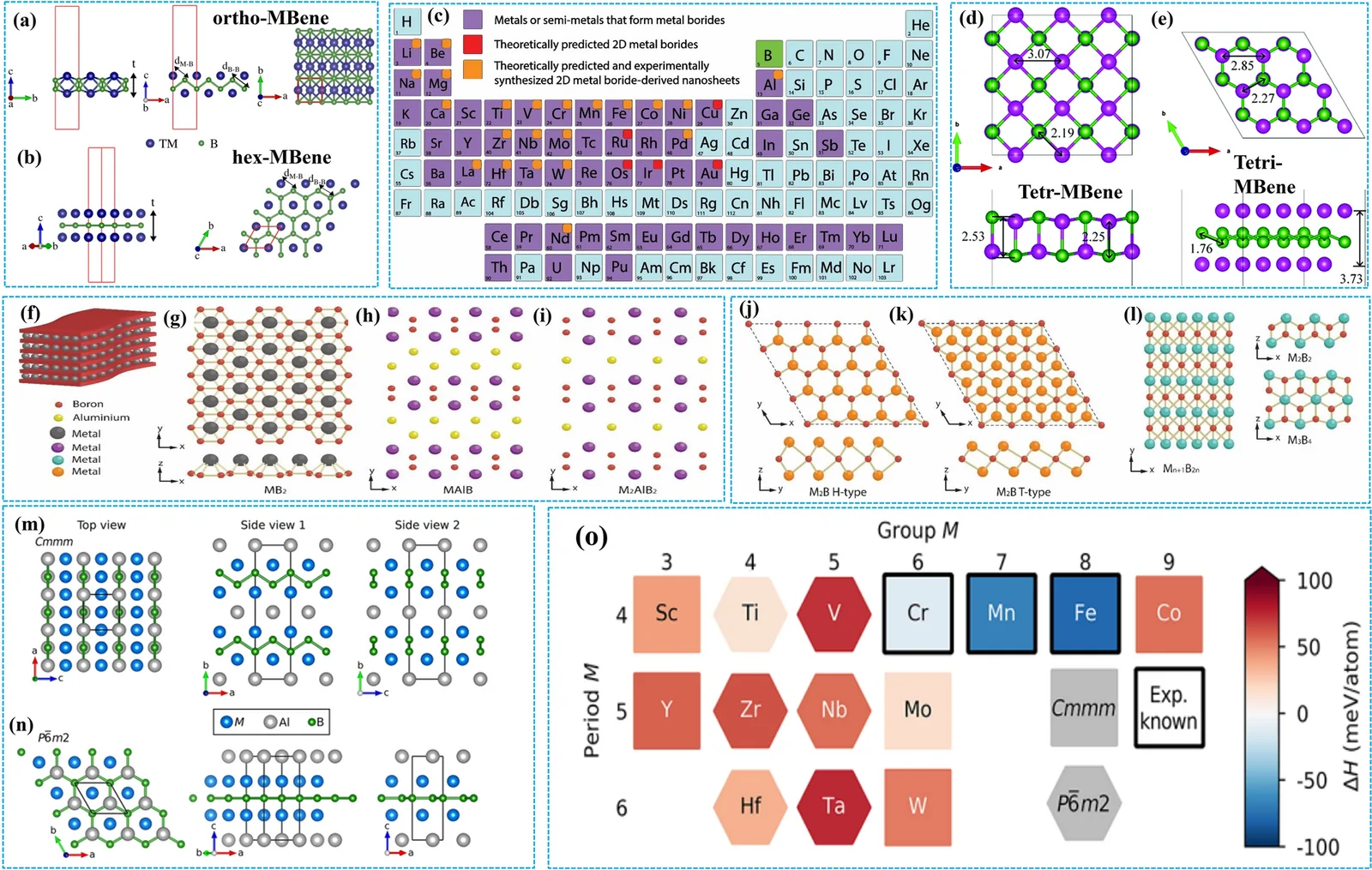
Structural representation of the **a** orth-MAB, **b** hex-MAB (adapted with permission [36]), **c** representation of the element that makes MAB and MBene phase (adapted with permission [37]), side and top views of the, **d** tetr-Mo_2_B_2_ monolayer and **e** tri-Mo_2_B_2_ monolayer. The Mo and B atoms are shown in violet and green color (adapted with permission [39]). Crystal assemblies of metal borides include: **f** layered metal borides, **g** MB_2_ type, **h** MAlB-type MAB phase, **i** M_2_AlB_2_-type MAB phase, **j** H-type M_2_B, **k** T-type M_2_B, and **l** M_n+1_B_2n_ type showing M_2_B_2_ and M_3_B_4_ phases (adapted with permission [37]). The stability characteristics of M_2_AlB_2_ with **m** orthorhombic Cmmm, **n** hexagonal P6̅m2 symmetry, delineated by unit cells indicated with black lines. **o** Theoretical phase stability calculations for M_2_AlB_2_, determining whether Cmmm (square) or P6̅m2 (hexagon) exhibits the lowest energy (adapted with permission [38])

The 2015 unveiling of the advanced 2D material MBene was carried out by Ade and Hillebrecht [11]. There is no precise definition available for MBene. Several scientific studies were improperly employed to characterize distinct 2D transition metal borides as MBene. The initially discovered MBene W_2_B_2_, Mo_2_B_2_, Cr_2_B_2,_ and Fe_2_B_2_ possessed orthorhombic nature [35]. These MBenes included various advantages or features, such as sandwich-like layered architecture and orthorhombic crystal systems. It and its parent material have the general chemical formula M_2_B_2_ and MAB, respectively. Based on crystal symmetries, MBenes are classified as ortho-MBenes and hex-MBenes. The parent material of the orthorhombic MBenes and hexagonal MBenes are represented by the orth-MAB and hex-MAB, respectively. Orthorhombic MBenes have a crystal architecture distinguished by three mutually perpendicular axes of varying lengths (a ≠ b ≠ c) [36]. Asymmetrical characteristics result from this structure, which means that the physical characteristics of the crystal can change according to the direction from which it is seen within the crystal. Due to this asymmetry, orthorhombic MBenes have the potential to exhibit exceptionally distinct electrical, magnetic, and mechanical properties. As opposed to hexagonal MBenes, which possess an arrangement of crystals in which two axes are of similar length and the third axis varies (a = b ≠ c), the angles between the axes are 120° and 90°, respectively [36]. As a result of this symmetry, their isotropic characteristics in the plane can be distinguished from those of their orthorhombic counterparts. In addition to having electronic solid conductivity, mechanical durability, and thermal resistance, hexagonal MBenes can also have other notable features. The structural representation of the orth-MAB and hex-MAB is presented in Fig. 4a, b. The selective elements used from the periodic table to synthesize the MAB and MBene are presented in Fig. 4c. Bo et al. [38] also employed the CALYPSO code to predict two new kinds of MBenes: tetragonal and trigonal Mo_2_B_2_ MBene. Their findings also show that the tetr-Mo_2_B_2_ and tri-MoB_2_ MBene possess promising stability and electrical conductivity. The synthesized tetr-Mo_2_B_2_ MBene possesses the first lowest energy per atom, 0.093 eV, and tri-Mo_2_B_2_ MBene possesses the second lowest energy, as presented in Fig. 4d, e, so tera-Mo_2_B_2_ MBene is more stable than the tri-Mo_2_B_2_MBene. Their groups also predicted the charge storage capacity of the tera-Mo_2_B_2_ MBene and tri-Mo_2_B_2_ MBene in SIBs and lithium-ion batteries (LIBs). The charge storage capacity of the tera-Mo_2_B_2_MBene is higher than that of the tri-Mo_2_B_2_ MBene. Gunda et al. predicted the structure of various MBene and MBene parent materials presented in Fig. 4f-l [37]. Dahlqvist et al. [38] also used theoretical analysis to predict the structure of the MBenes, as presented in Fig. 4m–o.

## Synthesizing Process of the MBene

The synthesis route of the material and the parameters applied during synthesis play a crucial role in altering the material’s properties, particularly its structure and chemical composition. The synthesis of solid nanomaterials is critically influenced by pH and temperature, key parameters for altering the material’s unique properties. The synthesis pathways are categorized into chemical and physical types. The physical approaches are ecologically sustainable and vice versa. This section examines the different synthesis methods of MBene and their effects on the structure of the produced MBenes. The synthesis routes of MBene are very similar to those of MXene. The primary difference in synthesizing both materials is that MBene and MXene are synthesized under mild and harsh conditions [40]. The extreme condition for MXene is required for its strong bonding between layers, compared to MBene.

**Fig. 5:**
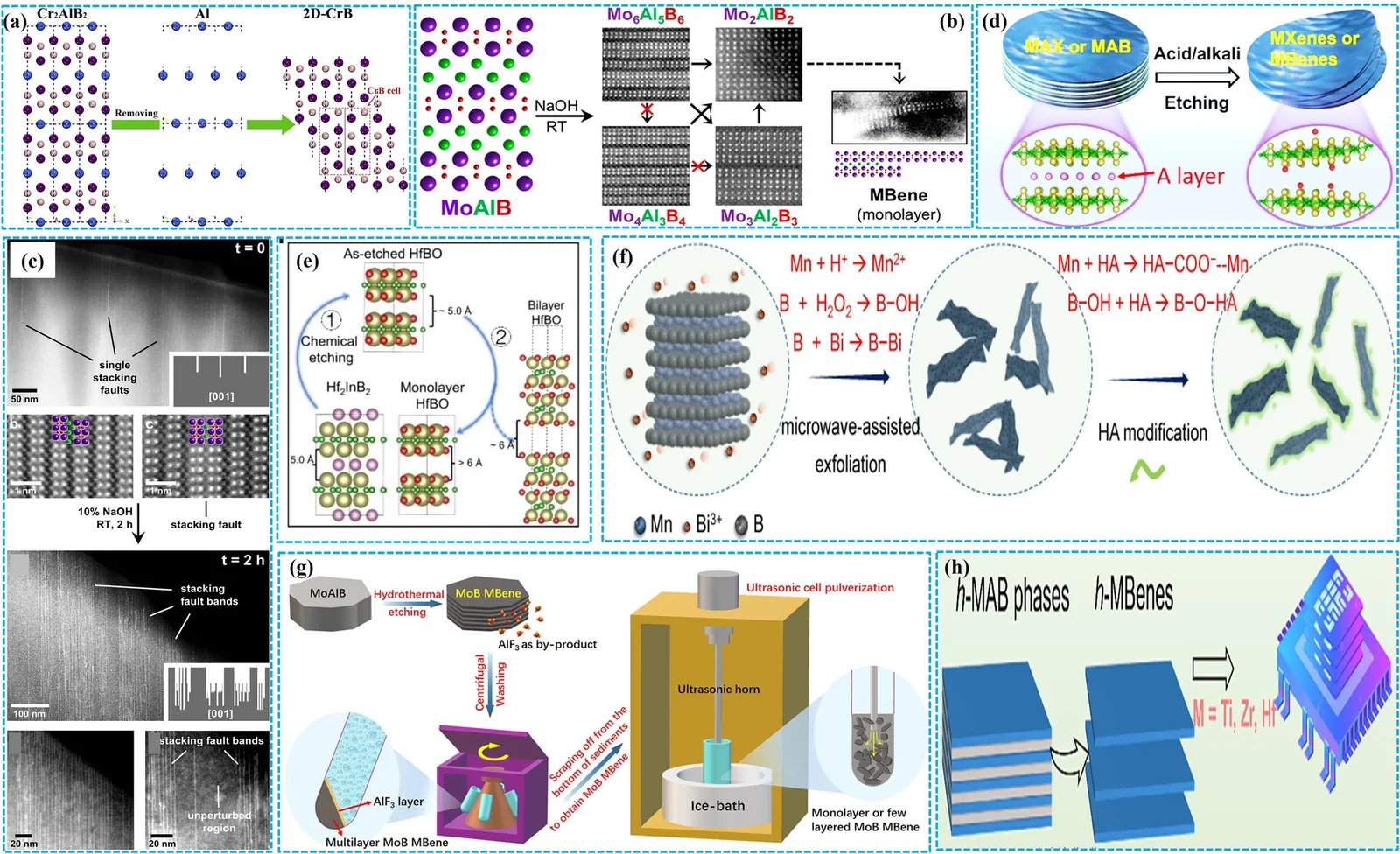
**a** Dilute hydrochloric acidic solution was used as an etchant agent to eliminate Al from Cr_2_AlB_2_ and synthesize CrB MBene nanosheets (adapted with permission [41]). **b** Synthesizing process of the MBene MoB MBene and DF-STEM images. **c** Intensity of the stacking faults on MAB and MBene before and after synthesizing (adapted with permission [42]). **d** Synthesized h-MBene by elective removal of the Al from h-MAB Hf_2_InB_2._ **e** A diagrammatic illustration of the mechanism by which h-MAB phase Hf_2_InB_2_ is converted into h-MBene HfBO (adapted with permission [15]). **f** Synthesizing process of the microwave-supported chemical exfoliation to obtain MnB_2_/MnB (adapted with permission [43]). **g** Synthesizing process of the MoB MBene from MoAlB by hydrothermal etching (adapted with permission [44]). **h** Schematic depiction of the h-MBene production process by selective etching of A layers (adapted with permission [45])

Most current research on 2D MBenes continues the remarkable effort to produce fundamentally thin, delaminated 2D MXene flakes. Scientists have employed two distinct methodologies regarding the structure of the etchant and the starting materials. The approach of the first category involves subjecting MAB phases to either acidic or basic treatments as preliminary materials. The second category focuses on applying bulk granules and their subsequent solvothermal defragmentation into distinct nanoparticles. Additionally, dealloying is another effective and solid technique for synthesizing MBene. In the first case, the produced CrB, MoB, and TiB were synthesized by eliminating the Al from their parent materials, MoAlB, Cr_2_AlB_2_, and Ti_2_AlB_2_, through complete and partial etching of the Al layer. A dilute hydrochloric acid solution was used as an etchant agent to remove the Al layer from the parent Cr_2_AlB_2_ material and synthesize the CrB MBene nanosheets [41]. The comprehensive production process of 2D CrB from its parent material, Cr_2_AlB_2_, by eliminating Al is presented in Fig. 5a. The synthesis process is prolonged, taking seven days to eliminate the Al layer from its precursor at room temperature. The synthesized product possessed a high purity, so functional groups did not exist on the interface of the final product.

The 2D nanosheets have a lamellar morphology and a thickness ranging from several to 10 nm. Alameda et al. employed the LiF/HCl solution technique to remove the Al layer from MoAlB, thereby synthesizing MoB [42, 46]. The process did not eliminate the Al from the parent material, so the final product is not pure; it possesses a small amount of Al residue. The synthesized material, in the form of 2D sheets, was kept at 50 and 300 nm thicknesses with the Al residue. The microscopic investigation by annular dark-field scanning transmission electron microscopy (ADF-STEM) of the synthesized 2D MBene sheet indicated that the partial etching of the Al created stacking faults to develop the etched cavities on the surface of the 2D MBene sheets, as presented in Fig. 5b. Mo_2_AlB_2_ and Mo_3_Al_2_B_3_ are two novel MAB intergrowth phases formed when Al is topo-chemically deintercalated from MoAlB. These defects generate substantial stacking layers. Therefore, the current LiF/HCl solution technique is not feasible for synthesizing MBene. Removing the Al layer from MBene compared to the MAX phase is particularly impractical. In MXene, the identical approach was employed to synthesize multilayered MXene by eliminating the Al layer within 24 h [47]. The Al single layer at the stacking fault replaces the zigzag Al two-layered structure of MoAlB in a linear pattern. The intensity of the fault in the synthesized material at various stages of synthesis is presented in Fig. 5c. Miao et al. [13] employed theoretical and experimental techniques to synthesize the MBene-based anode material, investigating its charge storage performance. They synthesized the h-MBene by the elective elimination of the In layer from the parent material h-MAB Hf_2_InB_2_. The mechanism of the selective removal of the ln from the MAB phase to synthesize the MBene is presented in Fig. 5d. The etching and exfoliation of the parent material of MBene have been achieved in two steps. Initially, In was eliminated from the parent material Hf_2_InB_2_, and an O atom created the bond at the interface of the Hf layer. In the second step, the space between two HfBO monolayers will eventually contract, forming a bilayer HfBO because O atoms are smaller than Hf atoms. This will cause the space adjacent to the monolayer to expand or exfoliate. The detailed process of etching and exfoliating the ln layer from the h-MAB phase Hf_2_InB_2_ to the h-MBene phase HfBO is presented in Fig. 5e. The discovery of h-MBene HfBO introduces a fresh addition to the growing family of two-dimensional compounds and allows for several distinctive applications.

To solve the problem of partial etching of the A layer from the MAB phase, Wang et al. [12] employed the thermally deployed technique to prepare the 2D TiB MBene by almost eliminating the ln layer from Ti_2_InB_2_. While synthesizing the Ti_2_InB_2_, the impurities generated on their interface were Ti_3_ln, Ti_2.2_ln_1.8,_ and Ti_3_In_4_. To remove these impurities, a dilute solution of HCl was prepared to dip the final sample in the solution for 10 h to eliminate all impurities and reduce the likelihood of impurities at the interface of the final TiB MBene product. Still, this post-treatment technique limited the trace amount of 6.3 wt% of TiB_2_ that exists on the interface of the MAB phase, which possessed 93.7 wt% of Ti_2_InB_2_. It is impossible to eliminate the remaining traces of TiB_2_ from Ti_2_InB_2_ because of the robust stability of TiB_2_ versus Ti_2_InB_2_.

Additionally, nitric acid was used as an etchant to remove the impurity from the remaining amount, but it was ineffective. Unfortunately, it caused a new impurity, TiO_x_. So, the synthesized sample Ti_2_lnB_2_ was placed into a dynamically evacuated quartz tube to synthesize pure TilnB_2_ phase at a temperature of 1050 °C for around a week under working pressure 10^−4^ Pa. Fan et al. [48] applied the ultrasound-assisted chemical etching method to synthesize the pristine 2D MgB_2_ MBene from MgAlB_2_ by eliminating Al. In the solution of (polyethylene glycol) PEG-4000 and bulk MgB_2_, 9 ml of H_2_O_2_ and 540 µL CH_3_COOH gradually have been mixed. Next, a pulsed sonicator mode (2.2 son /6.6 s off) ultrasonic treatment was administered for 60 min at 425 W cm^−2^. The synthesized sample of MBene consists of a large amount of targeted MgB_2_ MBene and a small number of traces of MgB_4_ phases. Using microwave assistance, Jin et al. [37] synthesized the manganese boride (MnB) MBene nanosheet. This method used microwaves to help with the reactive acidic etching of MnB, based on previously seen problems during MAB etching. In this process, two etching agents, CH_3_COOH and H_2_O_2_, are used to eliminate the A layer from the MAB step. It takes two h for the microwave generator to heat the initial mixture to 160 °C. After two h, the synthesized sample was washed, and its surface was modified with hyaluronic acid (HA) by sonicating it for an hour. The formation of a bond between bismuth and boron was most likely facilitated by including Bi^3+^ during the etching process, resulting in Bi-anchored MnB_2_/MnB nanosheets (MBBN). The steps used to make MnB_2_/MnB nanosheets by microwave-supported chemical stripping of the MAB phase are shown in Fig. 5f.

Chen et al. [49] The same microwave-associated chemical etching technique was utilized to synthesize the ZrBe nanosheet modified by HA (170 mg) with raw powder (385 mg) and CH_3_COOH dissolved in ethylene glycol (50 mL, EG). Additionally, H_2_O_2_ (3 mL) was added to the mixture and shifted into an autoclave at 160 °C for 2 h. HA variation of the surface process included washing, ultrasonography treatment, and the dispersion of 2D flakes with HA. There is no valuable quantity of supplementary efficient groups oversynthesized 2D MBene flakes. Sahu et al. [49] described the physical vapor deposition technique to eliminate Al from MoAlB, thereby synthesizing pristine MoB MBene. The synthesis process is costly due to the use of an expensive magnetron sputtering machine to eliminate Al. In general, the information showcased here highlights the unexplored possibility of using magnetron sputtering to directly create MBene in MoAlB/AlOx layered heterostructures. Contritely, the aforementioned groundbreaking works did not involve any experimental applications. The problem mentioned above significantly hampered extensive academic research into MBene. Jin et al. [50] experimentally synthesized the 2D MoB MBene by employing the hydrothermal etching route, by removing the Al from MoAlB. The detailed process of synthesizing MBene is described in Fig. 5g. The approaches mentioned for synthesizing 2D MBenes clearly demonstrate the need to develop a successful technique for isolating 2D MBene from substantially MAB-based mixtures. It is imperative and strongly advised to do in-depth research using cutting-edge, high-resolution materials characterization techniques on how the delamination process affects the final surface chemistry. The chemical composition of the parent material of MBene and the impact of the synthesizing route on the chemistry of the synthesized material is given in Table 1.

**Table 1:** Chemical composition of the parent material of MBene and the impact of the synthesizing route on the material’s chemistry

MBene Parent Material	Synthesized MBene	Synthesizing			Thickness of synthesized Sheet	Impact of Synthesizing Route	Surface Chemistry	References
		Method	Temp (k)	Time (h)				
Cr_2_AlB_2_	CrB	HCl Solution	RM	168	Several-10 nm	High-purity product, Complete etching of Al layer	No function group exists on the interface of the product by EDS analysis because of the high purity of the product	[41]
MoAlB	MoB	LiF/HCl solution technique	RM	24	50–300 nm	Low-purity Al residues are presented on the interface of the final product	The synthesized material’s poor purity is responsible for the targeted material’s limited stability and poor performance. So, the current technique should be replaced by an alternative one	[42]
Ti_2_InB_2_	TiB	Dealloying strategy	1050	144	10–20 nm	The synthesized sample is almost pure because no ln residue is present on the interfaces of the final product	The ln layer was slowly eliminated from the Ti_2_InB_2_ phase by increasing the synthesized pure TiB MBene temperature	[14]
MgAlB_2_	MgB_2_	Ultrasound-assisted chemical etching	RM	1	30 nm	Synthesized 2D nanosheets	B-O-PEG with HO-B on the MgB2 interface	[48]
Cr_2_AlB_2_	CrB	Microwave-assisted chemical exfoliation	433	2	10–100 nm	Exfoliation of the MAB phase, resembling that of the exfoliated graphite and the phases of MAX, was seen after 6 h of etching in diluted HCl	The presence of Cl or OH groups on nanosheet surfaces	[43]
ZrB_2_	ZrB	Microwave-assisted chemical exfoliation	433	2	7.4 nm	The pure ZrB_2_ initial powder was incorporated into single nanosheets, preserving its crystal structure	Borate ester development occurs between nanosheets following HA treatment. Compared to flakes that were not altered, this made it possible to stop the aggregation of ZrB nanosheets	[49]
MoAlB	MoB	Multistep low-temperature topotactic	873	4.5	–	Phase changes of MoAlB/Mo_2_AlB_2_ caused Al to substantially deintercalate, resulting in an inert nanosheet hetero structure modification with 2D Mo_2_AlB_2_ layers and unstructured layers of aluminum oxide. Only the outermost layer was etched using MoAlB	The development of a surface layer of aluminum oxide on the interface of the synthesized material	[51]
MoAlB	MoB MBene	Hydrothermal Etching	380–430	MoAlB	–	The synthesized MBene possesses multilayered MoB MBene with an accordion-like structure	In the process of the synthesis of the material, the formation of a surface layer of aluminum oxide on the contact	[50]

## Unveiling the Unique Characteristics of MBenes

The selection of the material as an electrode in energy storage and conversion is right relative to the material’s properties. The material properties are poor, which means it possesses poor electrochemical properties. In this section, we discussed the electrochemical properties of the 2D MBene. The stability of the electrode material during the process of charging and discharging plays a crucial role in their selection as electrode material.

### Mechanical Stability of the MBenes

**Fig. 6:**
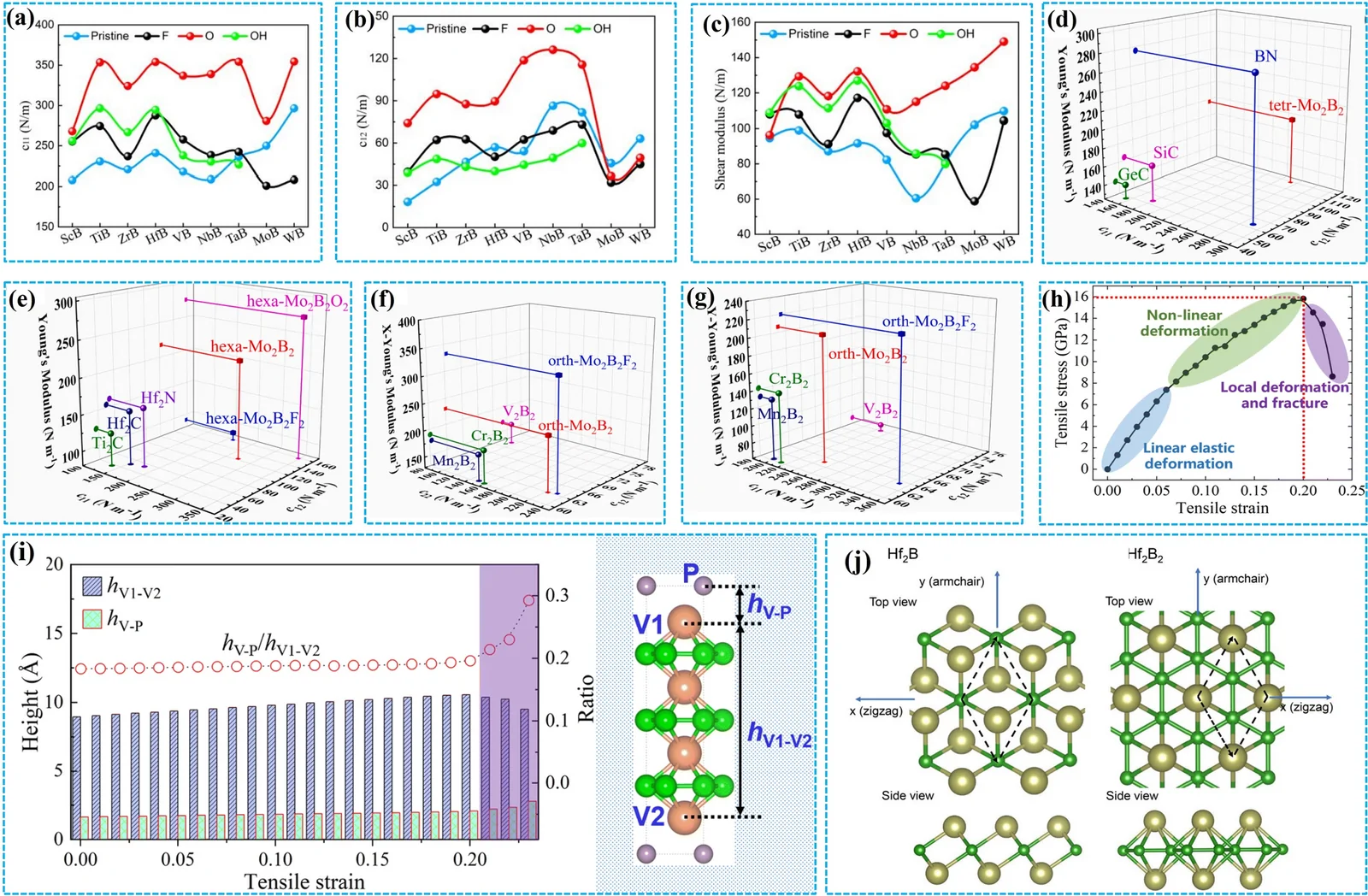
Investigation of the mechanical properties of the MBene, **a** c11, **b** c22, **c** Shear modulus (adapted with permission [52]). **d–g** c11, c12, c22, and Young’s modulus (Y) of pristine and functionalized MBenes (adapted with permission [53]). **h** Stress–strain curve for hex-V4PB6 by applying stress along the c-axis. **i** Structural deformation of hex-V_4_PB_6_ under tensile strain (adapted with permission [54]). **j** Schematic illustration of applied uniaxial and biaxial strain on 2D Hf_2_B and Hf_2_B_2_ (adapted with permission [55])

The capability of an MBene to sustain mechanical loads and strains without experiencing a structural breakdown is called MBene mechanical stability. The evaluation of specific parameters such as elastic constants, stress–strain curves, Young’s modulus, Poisson’s ratio, and fracture toughness can analyze the mechanical stability of the MBene. MBene must be used in flexible electronics or mechanical reinforcement to possess high mechanical stability, for example, the elastic constant c_11_ and c_12_ of the ScB (207.78 and 18.18), ScB-F (255.32 and 39.57), ScB-O ( 267.95 and 74.17), ScB-OH (256.02 and 38.86), respectively [52]. The results indicate that the unmodified MBene exhibits more severe mechanical stability than the functionalized MBene. Similarly, -O-functionalized MBene possesses an excellent elastic constant compared to the –F- and -OH-functionalized MBenes, as presented in Fig. 6a, b. The same results were observed in MXene, which strongly bonds the -O atom with its surfaced transition metal compared to the -F and -OH [56–59]. The findings suggest that functionalized MBenes exhibit greater mechanical rigidity than the corresponding pristine MBenes. Similar to the MBenes, their surface terminations were expected to enhance the mechanical stiffness of MXenes compared to the pristine MXenes [60]. The Poisson’s ratio of the predicted MBene changes from 0.087 to 0.414. Specifically, the Poisson’s NbB-F, NbB-O, and NbB-OH ratios are 0.37, 0.29, and 0.24, respectively. The Poisson’s ratio is higher for the -O-terminated MBene than -F and -OH MBene. The material’s response to shear stress is referred to as the shear modulus, and the rigidity of a system may be determined by the shear modulus value, which can be either big or small. Their findings proved that a large force is required to deform the -O-functionalized MBene as compared to the pristine and –F- and -OH-functionalized MBenes, as shown in Fig. 6c. Young’s modulus quantifies the relationship between strain and uniaxial stress in the direction of tensile application. The Young’s modulus of the pristine TiBMBene, TiB–O, TiB–F, and TiB–OH MBene are 255, 330, 260 and 270 N m^−1^, respectively [52]. Similarly, the MBene functionalized with oxygen possesses superior mechanical stability compared to the -F and -OH functionalization. Generally, the material’s thickness also has a valuable impact on Young’s modulus (Y) of the MBene; for example, MBene thickness increases from 0.8 to 1.0 nm, and MBene Young’s modulus increases from 200 to 320 GPa [52]. Liu et al. [53] employed theoretical calculations to examine the influence of surface functionalization on the mechanical properties of the hexagonal and tetragonal Mo_2_B_2_ MBenes. The surface functionalities on the surface of MBene have a viable impact on its stability. Still, the functionalities of the MBene have no impact on the bonding features of the Mo–B and B–B bonds, which are the same but possess different stability. The hex and tetr-phase of MBenes have two independent quantities, C_11_ and C_12_, and the orth-phase of MBene possesses three independent quantities, C_11_, C_22_, and C_12_. The Young’s modulus and Poisson’s ratio of the tetr-Mo_2_B_2_ are 197.150 N/m and 0.47. The comparative analysis of Young’s modulus and Poisson’s ratio of tetr-Mo_2_B_2_ with BN, SiC, and GeC is presented in Fig. 6d. The finding from the figure shows that Young’s modulus of the MBene is slightly smaller than the BN and more significant than the SiC and GeC, but MBene Poisson’s ratio is more significant than BN, SiC, and GeC. The Young’s modulus (217.408 N m^−1^) and Poisson’s ratio (0.450) of hexa-Mo_2_B_2_ is greater than the Hf_2_N, HF_2_C, and Ti_2_C as shown in Fig. 6e. The –F functionalization also impacts the poison ratio of the orth-Mo_2_B_2_ MBene. The pristine and functionalized orth-Mo_2_B_2_ poison ratios in the x and y directions are 0.292, 0.311, 0.178, and 0.269, respectively. The Poisson’s ratio of the pristine orth-MoB_2_ MBene is greater than the functionalized MBene in both the x and y directions. So, the functionalized orth-MoB_2_ MBene possessed more resistance to word deformation against the applied force than the unfunctionalized MBene because the Poisson ratio enhanced the material’s porosity. The porous material possessed a high Poisson’s ratio and low mechanical stability and vice versa. So, the authors can say that pristine orth-MoB_2_ MBene possessed high porosity compared to the functionalized orth-MoB_2_ MBene. So, pristine orth-MoB_2_ MBene has low mechanical stability compared to the functionalized orth-MoB_2_ MBene. Additionally, Young’s modulus of the hexa- Mo_2_B_2_F_2_ and hexa-Mo_2_B_2_O_2_ are 91.034 and 275.408 N m^−1^; these findings show that the -O-functionalized MBenes possess higher mechanical stiffness than the -F-functionalized MBenes, the functionalization did not valuably impact the Poisson’s ratio of the MBene. The orth-Mo_2_B_2_ MBene has a much higher mechanical strength in the x and y directions (203.261 and 216.579 N m^−1^, respectively) than the other expected MBene structures. In the x and y directions, the Poisson’s ratio of the orth-Mo_2_B_2_ MBene is lower (0.292 and 0.311, respectively) than that of the other expected MBene structures. Their findings show that the mechanical stiffness of MBene increases, and their Poisson’s ratio decreases after the -F functionalization of MBene, as presented in Fig. 6f-g.

Li et al. [54] inspected the effect of longitudinal stretching on the mechanical characteristics of the hex-V_4_PB_6_ MBenes. The generated stress–strain curve delineates three phases: linear, nonlinear, and local deformation zones, characterized by fast collapse corresponding to fractures of less than 6%, 20%, and higher than 20%, respectively, as seen in Fig. 6h, i. Figure 6j delineates the particulars of the lattice deformation examined in this work. The distribution of electrons around the V and B atoms remained primarily unchanged during tensile strain, indicating that the V–B and B–B bonds exhibited significant resistance to tensile stress. Nonetheless, the electrons became highly concentrated around the phosphorus atoms. The ELF value for P being almost one at a strain of 23% signifies that the V–P bond was significantly compromised under tensile stress. Based on these findings, it can be concluded that the point of origin for the collapse of the hex-V_4_PB_6_ lattice would be the crack between the V and P layers as a result of tensile stress. Young’s modulus of the MBene and MXene was predicted to be 200 to 320 [52] and 400–1000 GPa [32, 56, 61, 62]. The elevated value of Young’s modulus is contingent upon the composition, surface functional groups, layered thickness, and the number of layers in MBene and MXene.

Based on the above results, MXene is stronger than MBene, which is why functional groups increase the mechanical stability of the MXene. Adding the functional groups on the MBene layers develops a strong bond with transition metal atoms to enhance the bonding force within the MBene structure. Stronger bonds give the substance additional mechanical deformation resistance. Additionally, the defect (unsaturated bonds) on the interface of the MBene reduced their mechanical stability. The attachment of the functional groups on the interface of the MBene reduced these defects in the interface of the MBene to make it more robust against mechanical forces.

### Thermal Stability of the MBene

The structural degradation resistance of MBene against the temperature is demonstrated by its thermal stability, which may affect the battery’s structure and efficiency. The high thermal stability of the electrode material inhabits electrode material structural degradation, particle detachment, or electrolyte leakage of the MBene during the long cycling performance under strong operating conditions [63]. The thermal stability of the MBene is of significant consequence in various applications, including electronics, catalysis, and energy storage. The cohesive energy is a primary factor that analyzes the thermal stability of the crystalline materials. Coherent energy is the amount required to combine or separate the constituent of a crystalline substance. The high cohesive energy of the material indicated their higher stability. The coherent energy of bare MnB MBene, silicone, phosphorene, and germanene are 4.95, 3.91, 3.48, and 3.24 eV atom^−1^, respectively [64]. These findings show that more energy is required for MBene to disassemble a crystal MBene into its atoms than other 2D materials. So, MBene possesses higher thermal stability than 2D materials.

The higher cohesive energy is responsible for the solid structural stability of M_n_B MBene than the other 2D materials. These findings suggest that monolayer M_n_B exhibits thermodynamic stability and high potential for synthesis in experimental conditions. Another study also investigated the thermal stability of the MBene, the monolayered M_n_B MBene, and their bulk MnB crystal, which possesses a cohesive energy of 4.80 and 5.35 eV atom^−1^. The cohesive power of the bulk MnB crystal is slightly more significant than the monolayered MnB MBene. Still, the layered MnB MBene has better cohesive energy than recently synthesized Mn-based MXene, such as Mn_2_C and MnN_2_, which is 4.42 and 3.45 eV per atom, respectively. The elastic constant, C_11_, C_12_, C_22_, and C_44_ of MBene are 197, 50, 158, and 90 N m^−1^. These findings show that the synthesized MBene has good mechanical stability because it touches the Born–Huang stability criteria [27]. Then, the synthesized MBene possesses dramatic thermal stability up to 1200 k after 20 ps because no chemical alteration occurred in the parent material MBene, while the average movement of Mn atoms is approximately 0.08 Å, it can be concluded that the framework of Mn atoms stays unaltered. This finding shows that MBene possesses excellent thermal stability compared to many synthesized MXenes [27]. Massod et al. [63] investigated the impact of the temperature on the thermal stability of the Ca_6_CrB_4_, Ca_6_MoB_4_, and Ca_6_WB_4_ MBenes. Their theoretical findings show that the MBene’s acceptable structural alteration occurred in the MBene-based electrode at 300 K. The minor structural changes at 300 K did not significantly affect the MBene-based electrode material’s performance. These findings show that the MBene-based anode material has excellent electrochemical performance at higher temperatures. The higher cohesive energy or the thermal and structural stability of the MBene is because of their excellent association with the constituting elements and their rectangular structure. The variation in the magnetic behavior of the MBene with the enhancement of the temperature was employed to investigate the thermal stability of the MBene. They found that the MBene’s magnetic nature transfers into a paramagnetic state by continuously enhancing the temperature up to Tc = 548 K. This means that the MBene is thermally stable at up to 548 K [65–67].

### Electrical Properties of the MBene

**Fig. 7:**
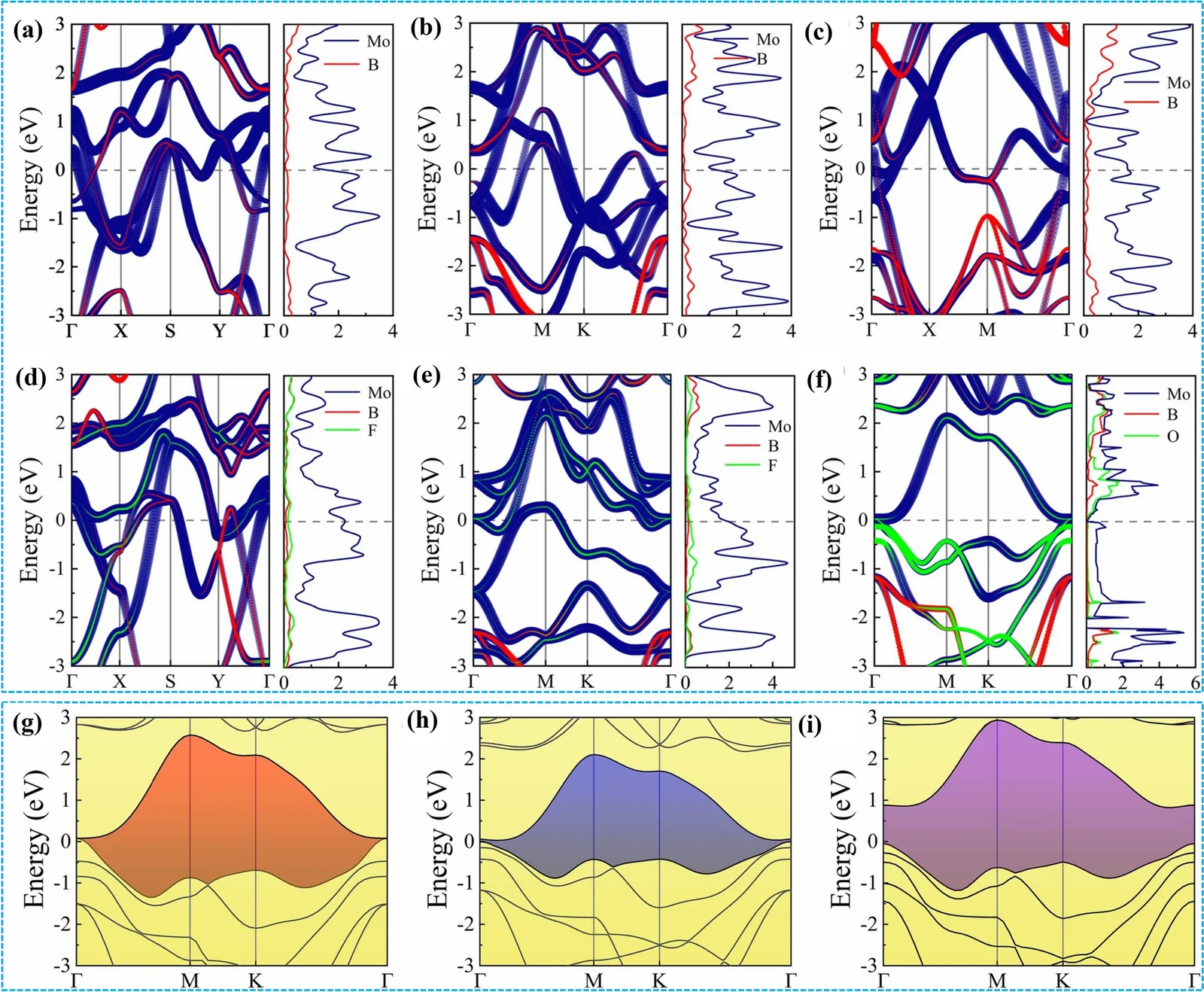
Band assemblies and PDS for **a** orth-Mo_2_B_2_, **b** hexa-Mo_2_B_2_, **c** tetr-Mo_2_B_2_, **d** orth-Mo_2_B_2_-F_2_, **e** hexa-Mo_2_B_2_-F_2_, **f** hexa-Mo_2_B_2_-O_2_ monolayers, Band structures of hexa-Mo_2_B_2_O_2_ single layer by using, **g** HSE06, **h** GGA + SOC, **i** HSE06 + SOC approaches (adapted with permission [53])

The electronic structure of the MBene can be evaluated effectively by employing theoretical calculations. There is an extensive range of possible variations in the electronic band structure of MBenes, which is determined by the transition metal and the particular arrangement of the boron atoms. Certain MBenes are metallic, indicating that they possess bands that can pass through the Fermi level, which results in elevated electrical conductivity. The configuration of the boron atom and transition metal significantly influences the performance of the MBene since the decrease of the band gap and enhancement are closely correlated with the arrangement of the MBene’s constituents. Liu et al. [53] calculated the electronic structure of the three Mo_2_B_2_ MBene phases using a theoretical analysis technique. The multiple energy band exists at the Fermi level in the various phases of the MBene; all these phases possess the metallic characteristic of MBene, as shown in Fig. 7. Additionally, it has been discovered that the Mo atom primarily contributes to the Fermi level. In contrast, the contribution of the B atom is limited inside this region. The adsorption of the functional groups –F and –O on the interface of the orth-Mo_2_B_2_F_2_ and hexa-Mo_2_B_2_F_2_ does not affect their metallic nature to a large extent because after the functionalization of various bands exists throughout the fermi level, and the existence of these bands maintained the metallic nature of the MBenes. However, hexa-Mo_2_B_2_O_2_ exhibits a dramatic shift in its structure. It can be observed that the only degenerative band in the Fermi energy level that hexa-Mo_2_B_2_O_2_ possesses is the one that is located near the Γ point.

Additionally, another theoretical method was used to extensively analyze the electronic structure of the hexa-Mo_2_B_2_O_2_, as presented in Fig. 7a-i. Following the completion of oxygen functionalization, the degenerated energy level displays a band gap of 0.0003 eV, and it begins to split at the Γ point. After considering the SOC impact, the degenerated energy level undergoes splitting at the direction of the Γ point. After careful analysis, it has been determined that the valence band reaches its highest position at the Γ point. These findings show that the MBene possesses an excellent electronic structure before and after its functionalization. Han et al. [68] employed the density functional theory (DFT) calculation to investigate the metallic behavior of the MB_2_ MBene before and after the absorption of the Li ions. It is essential to remember that modifying the morphology of MB_2_ MBene following the adsorption of Li atoms will change the electronic structure. The atoms in the MB_2_ layer are the primary contributors to the states located around the Fermi level, whereas the contribution of the Li atoms is negligible. The findings above demonstrate that the metal characteristics of MB_2_ monolayer can be well preserved both before and after incorporating Li atoms into MB_2_. This enables the electrical conductivity of the MB_2_ layer to fulfill the criteria of LIBs throughout the entirety of the procedure for charging and discharging. Because of this, the MB_2_ monolayer MBene is a perfect material for an anode since it possesses extraordinary electrical characteristics and storage capacity.

Bao et al. [39] utilized the DFT calculation to investigate the metallicity of the tetr-Mo_2_B_2_ and tri-Mo_2_B_2_ MBene. Their findings show that the tetr-Mo_2_B_2_ and tri-Mo_2_B_2_ MBene possess metallic characteristics due to the nonzero DOS at the Fermi energy. The metallic characteristics of the tetr-Mo_2_B_2_ and tri-Mo_2_B_2_ MBene originated from Mo 4*p* orbital and 4*d* orbital. It is impossible to overstate the significance of B 2*p*. Because of their exceptional electronic conductivity, these Mo_2_B_2_ single layers have the potential to be used in energy storage applications. In a shell, all the synthesized MBene possess an excellent electronic characteristic before and after the functionalization and storage of ions. So, MBene is an effective electrode because it sustains excellent electrical conductivity after utilization as an electrode material. The magnetic nature of the MBenes comes from their M-3*d* orbital. The availability of spring and down channels in the MBene shows its metallic nature, with excellent electronic conductivity for all discovered MBenes. So, MBenes possess a fast charge transfer rate during the reaction process. This higher conductivity is demonstrated by the steady density of states at 0 eV Fermi energy levels. Additionally, it is noted that the M atoms mainly offer to the dominating states surrounding the Fermi level, which are primarily made up of the M-3*d* orbital [69]. This suggests that the M atoms have an essential role in the conductivity features of MBenes. In conclusion, MBene exhibits significant promise for electrocatalysis and charge storage devices due to its exceptional lattice stability and high conductivity. In a nutshell, MBene possesses a rectangular structure and excellent electrical, thermal, dynamic, and structural strength. The strong bonding force between the layers of MBene is responsible for their magical stability.

## Surface Terminations: Modulating Interfacial Chemistry in MXenes and MBenes

The surface terminations of MXenes significantly affect their physicochemical and electrochemical characteristics. Traditionally, terminations like –O, –OH, and –F are included during etching and delamination procedures, influencing conductivity, ion intercalation, surface energy, and catalytic activity [70]. Recent studies have highlighted that variations in terminations not only alter surface charge and electronic structure but also influence interlayer spacing and wettability, and crucial parameters for energy-related applications [71–74].

In addition to traditional terminations, methods to modify MXene surfaces have been developed. High-energy techniques, including plasma irradiation, controlled atmosphere annealing, and molten salt procedures, can selectively remove or substitute terminations [75, 76]. Covalent functionalization provides a reliable method for incorporating organic groups or heteroatoms to modify features such as hydrophobicity, catalytic selectivity, or electrochemical activity [77, 78]. These solutions have been demonstrated to significantly enhance performance in applications such as electrocatalysis, supercapacitors, and sensors [79].

Significantly, these surface engineering approaches unveil new possibilities for MBene materials, a subclass of MXenes characterized by M denoting post-transition or rare earth metals. Although MBenes possess a 2D layered structure and are synthesized by an etching method similar to MXenes, they frequently display unique bonding properties and oxidation tendencies. Recent theoretical and experimental findings indicate that MBene terminations (e.g., –O, –S, –Cl) may exhibit enhanced stability due to variations in d-band filling and bonding energy, thereby influencing their surface reactivity and interactions with ions or molecules [80].

Furthermore, functionalized MBenes are emerging as adaptable platforms for imparting novel features that are not readily attainable in MXenes. S-functionalized CrBene and YBene exhibit improved catalytic activity and thermodynamic stability for hydrogen evolution reaction (HER) and carbon dioxide reduction reaction (CO₂RR), due to optimum adsorption energies influenced by surface groups [81, 82]. Covalent modification has shown potential to enhance hydrophobicity and electrochemical durability, rendering MBenes viable candidates for demanding energy storage conditions [83, 84].

The functional tunability of MBenes remains little investigated in contrast to MXenes. Nonetheless, the reduced electronegativity and coordination adaptability of post-transition metals may facilitate wider functionalization opportunities. This may provide precise regulation of electronic states and interfacial dynamics, perhaps revealing application-specific terminations in catalysis or solid-state batteries [85].

In conclusion, surface termination engineering is an essential aspect of MXene and MBene development. Although MXenes have undergone more systematic examination of functionalization and tuning techniques, MBenes provide a novel possibility with distinct benefits that need more exploration. Table 2 presents a comparative assessment to inform future experimental and theoretical endeavors [71–74, 80, 85, 86].

**Table 2:** Comparative examination of surface termination chemistry in MXenes and MBenes

Feature	MBenes	MXenes
Common terminations	–O, –S, –Cl, –Br, potentially –N and organics	–O, –OH, –F, –Cl, –S
Formation mechanism	Comparable techniques; fluorine-free and molten salt etching are being utilized more often	Generated during etching in HF or fluorine-based solutions
Tunability strategy	Covalent functionalization, substitutional doping, molten salt processes	Thermal annealing, plasma treatment, molten salts, covalent grafting
Stability considerations	Enhanced oxidation resistance for certain terminations (e.g., –S) due to metal–B bonding	Some terminations (e.g., –F) unstable in aqueous or high-temperature systems
Impact on electronic properties	Early studies show promising tuning of bandgap and work function with surface groups	Alters band structure, conductivity, Fermi level
Ion transport effects	Termination-dependent ion affinity not yet widely studied	–O, –OH favor Li⁺/Na⁺ intercalation; –F hinders
Hydrophilicity/ interlayer spacing	Hydrophobic terminations or steric groups could tune permeability and stability	Hydrophilic –OH, –O enhance dispersion and swelling
Application impacts	HER, CO₂RR catalysis, electrochemical sensors, beyond Li battery electrolytes	Supercapacitors, Li-/Na-/K-ion batteries, HER/ORR/OER catalysis
Key limitations	Lack of systematic experimental studies; stability under cycling and voltage range needs exploration	Difficult to control termination uniformity, removal of undesirable groups

## MBenes: Versatile and Adaptable

**Fig. 8:**
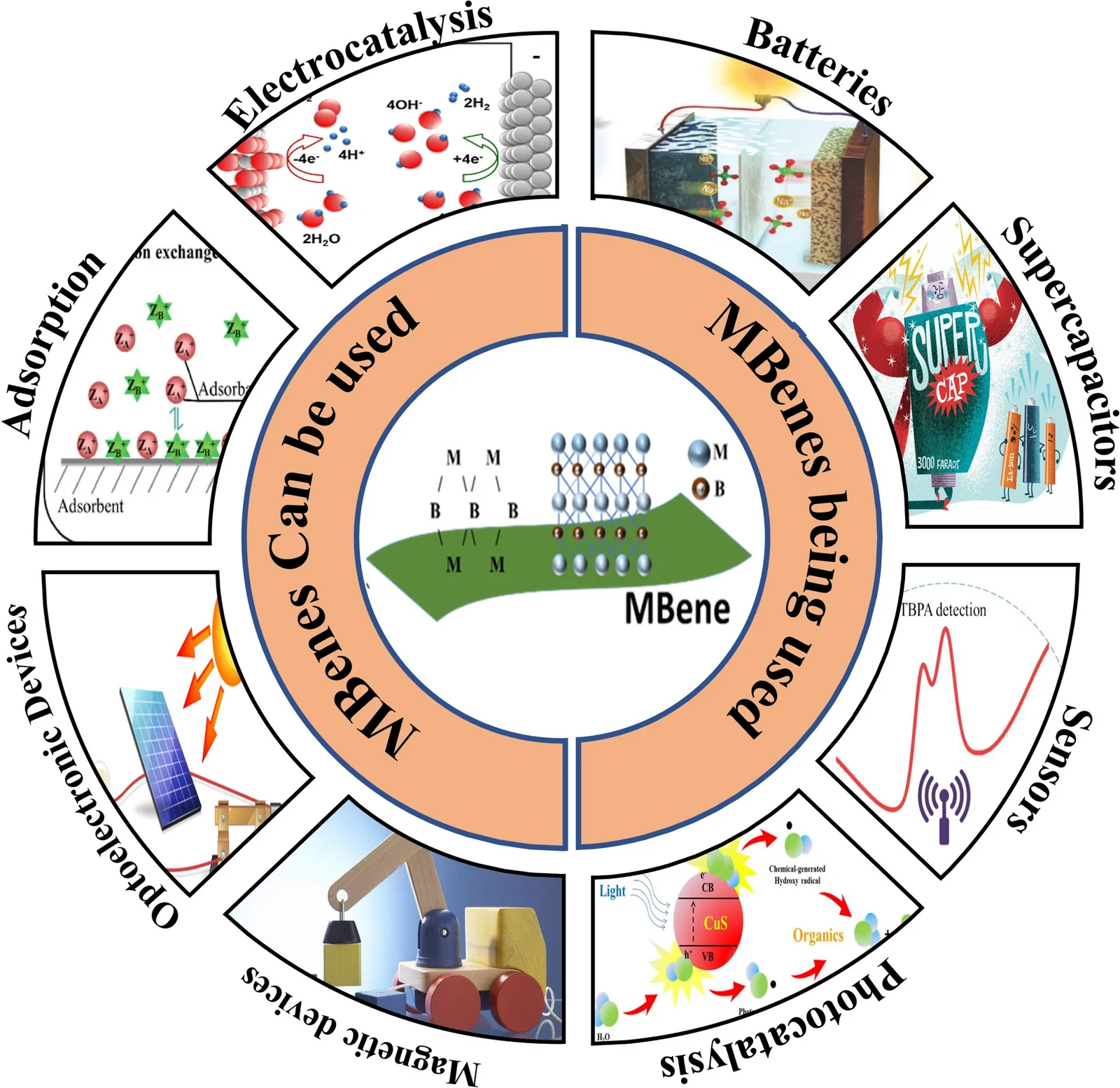
Schematic diagram showing MBenes and the fields where they can be applied

The MBene is an advanced member of the 2D material. The magical electrical and structural properties make MBene an ideal candidate in various fields. To date, limited experimental and theoretical studies on MBenes utilization are available. Few scientific investigations are available about the performance of MBene in LIBs, lithium–sulfur (Li–S) batteries, and supercapacitors. However, these findings show the excellent electrochemical performance of MBene as a cathode material. The theoretical studies demonstration that the high charge storage capacity of the Ti_2_BS_2_ and ScTiB_2_ MBene cathode material in SIBs and LIBs are 942 and 937 mAh g^−1^, respectively [87, 88]. The high charge storage capacity and cycling stability of the MBene are due to the significant SSA and good thermal and mechanical strength. The stability of the material is also a primary parameter in the electrode field for long-term cyclability. Ammonia is the primary source of the fertilizer sector, as well as resins, chemical fiber, and dyes. It is also extensively utilized as a hydrogen carrier and fuel source. Qi et al. [89] reported that the metallic electronic band structure and the electronic properties of the MBene are favorable for the nitrogen reduction reactions (NRR) catalytic activity. The existence of several extra active sites speeds up the NRR process and outstanding selectivity toward NRR. The limited potential of the screened TiB, YB, ZrB, and MoB MBenes are 0.64, 0.68, 0.65, and 0.68 V, respectively. The limited overpotential of the MBene makes MBene an ideal candidate for NRR reactions. Various theoretical studies on utilizing the MBene as an adsorbent, sensors [90], biomarker detection [91], HER, and OER are available [69]. The schematic diagram of the MBenes being used and where MBenes can be used are presented in Fig. 8.

## Maximizing Energy Potential: Choosing the Right MBenes

The electrochemical performance of MBene materials is significantly influenced by the number of layers in their structure. Single-layered MBenes are exceptionally advantageous for energy storage and harvesting applications owing to their elevated specific surface area (SSA), plentiful exposed active sites, and improved quantum confinement effects. These characteristics enable enhanced charge transfer, effective ion adsorption, and rapid electrochemical kinetics, all crucial for high-performance electrode materials [90, 91]. Furthermore, the uniform pore architecture and readily available surfaces of single-layered MBenes facilitate effective ion diffusion and optimize charge storage capacity. Conversely, multilayered MBenes possess a stacked structure that restricts interlayer accessibility and diminishes the total specific surface area, thereby hindering their electrochemical performance. The interlayer restacking characteristic of multilayered MBenes frequently results in slow ion transport, reduced usage of internal active sites, and attenuated quantum effects, akin to the behavior of bulk 3D materials.

Notwithstanding the exceptional electrochemical properties of monolayered MBenes, obstacles persist concerning their scalable fabrication and structural integrity under operational settings. The regulation of layer quantity is a crucial factor in enhancing MBene performance. Research indicates that few-layered MBenes (often 3–5 layers) provide an ideal equilibrium between mechanical strength and available electroactive surface area, enhancing charge adsorption kinetics [92–94]. Exfoliation strategies must be meticulously designed to provide such control. Insights from MXene processing indicate that ultrasonic-assisted exfoliation is a feasible approach [95]. By adjusting factors like as ultrasonic power, frequency, temperature, and sonication duration, one may affect the delamination efficiency, thereby regulating the ultimate quantity of MBene layers [96–98]. Increased ultrasonic power often improves exfoliation efficiency, although it may also cause structural faults; therefore, optimization is essential. Moreover, the interlayer spacing is critical; for example, MBenes with an interlayer distance of roughly 0.34 nm exhibited the greatest CO₂ adsorption capabilities [92, 99, 100]. In MXene-based membranes, best CO₂ separation occurred at membrane thicknesses of 10–20 nm, with performance diminishing at larger thicknesses due to reduced diffusivity [101]. Consequently, the thickness (number of layers) and interlayer spacing of MBenes must be meticulously optimized to achieve a balance between performance, structural integrity, and application-specific criteria. Single-layered MBenes provide enhanced electrochemical performance, but multilayered variants provide improved environmental stability and cycle endurance, underscoring the trade-off between efficacy and resilience that must be considered in practical device development.

## Evaluating MXenes and MBenes for Optimal Energy Solutions

In general, MBene and MXene are an excellent material and possess a promising material for energy storage and harvesting. MXenes have a greater surface area, which enables them to have a better capacity for adsorbing charges. In addition, the consistent pore structure of these materials makes it easier for charges to diffuse, which could result in an increased storage rate. MBenes, however, are exceptionally stable, particularly in humid situations. Because of their interlayer solid contacts, they offer higher resistance to oxidation and degradation, which is an essential advantage for long-term performance [102]. MBenes are inferior to MXenes in terms of their capacity for charge storage capacity and their efficacy in diffusion. On the other hand, MBenes become more persistent in humid environments. It depends on the specific use of the material most suitable for charge storage and conversion. In situations where charge storage capacity and diffusion performance are essential, MXenes stand out as the superior option. If staying stable in humid conditions is the most crucial consideration, then MBenes are the superior option. When selecting a material for charge storage, there are several other considerations to consider, in addition to the capacity for charge storage, the effectiveness of diffusion, and the stability of the material. These factors encompass cost, flexibility, and processing efficiency. Currently, MXenes are more expensive than MBenes; nevertheless, they are also easier to produce on a large scale. The production of MBenes on a large scale is more complex, but processing these compounds is simpler.

Aerogels based on MXene are substances being investigated for their possible high charge storage capacity and additional significant characteristics. Integrating micropores into these lightweight aerogels enhances their ability to adsorb various substances, such as organic solvents. MXene-based aerogels possess substantial surface area and porosity, attributed to their unique structure, resulting in superior adsorption capabilities [103]. Research on MXene aerogels has demonstrated that these materials may be manufactured without external support and possess a high charge storage capacity. Additionally, MXene aerogels have shown remarkable effectiveness in shielding electromagnetic interference, indicative of their multifunctional potential and diversity in architecture and chemistry [103–105]. There is evidence that composites based on MXenes, which combine MXenes with additional substances such as polyethyleneimine and metal oxides, have the potential to have a large capacity for charge storage. MXene-supported adsorbents exhibit exceptional performance and commendable cycle stability for charge storage. Increasing the mechanical strength of composites against attrition by incorporating MXenes indicates potential for their application in charge storage performance [106]. MBenes have the potential to become a more advantageous option as technology and understanding of these materials continue to progress. In conclusion, MXenes are currently more well known and adaptable for energy storage applications. The precise needs of the energy storage application will determine which of these two options is the best available.

## MBene as Energy Storgae Material

The charge storage devices are divided into categories, such as batteries and SCs. These charge storage devices are also divided into subclasses. This section presents the charge storage capacity of the pristine and MBene-based composite electrode materials across different batteries. In addition, we discussed the performance of the MBene-based electrode material in charge storage devices, especially in batteries [107].

### MBene-based Li-ion Batteries

Lithium-ion (LIBs) batteries charge the storage capacity of the rechargeable energy storage device extensively, which relies on the storage and release of the Li^+^ ion during the charging and discharging process. The 2D structure of MBenes provides a large surface area, which can enhance the capacity for lithium storage. The layered structure and many active sites on the interface of the MBene enhanced their charge storage capacity and retention of the metal ions. The high electrical conductivity of MBenes permits fast charge and discharge rates, enhancing the battery’s overall performance. The storage capacity of MBene-based electrodes also extensively depends on the ionic radius and valance electron of the storage metal ions. A small radius is easily inserted in the layered MBene compared to the large radius ions [108]. Additionally, the valence electrons and ionic radius affect metal ions’ adsorption, retention, and transport inside MBene. The theoretical investigations have revealed the incredible prevalence of MBene as a material for anodes LIBs and sodium-ion batteries (SIBs) [109, 110]. Scientists have been using rechargeable LIBs more and more since they were introduced to the market. They appeal because of their substantial capacity, high power density, extended cycle life, and exceptional energy efficiency. In LIBs, performance is closely correlated with the lithium storage capacity of the synthesized MBene-based anode material. Graphite is now utilized as an anode material in commercial lithium-ion batteries owing to its notable characteristics, such as high Coulomb efficiency, substantial cycle stability, and cost-effectiveness. The major hurdles in using graphite as an electrode material are limited theoretical Li storage capacity and stability. This deficiency does not satisfy the growing needs of the contemporary market. Therefore, it is essential to discover new anode materials that can significantly improve LIB performance. Several studies have provided compelling evidence of the significant role that MBenes play in improving LIB function.

**Fig. 9:**
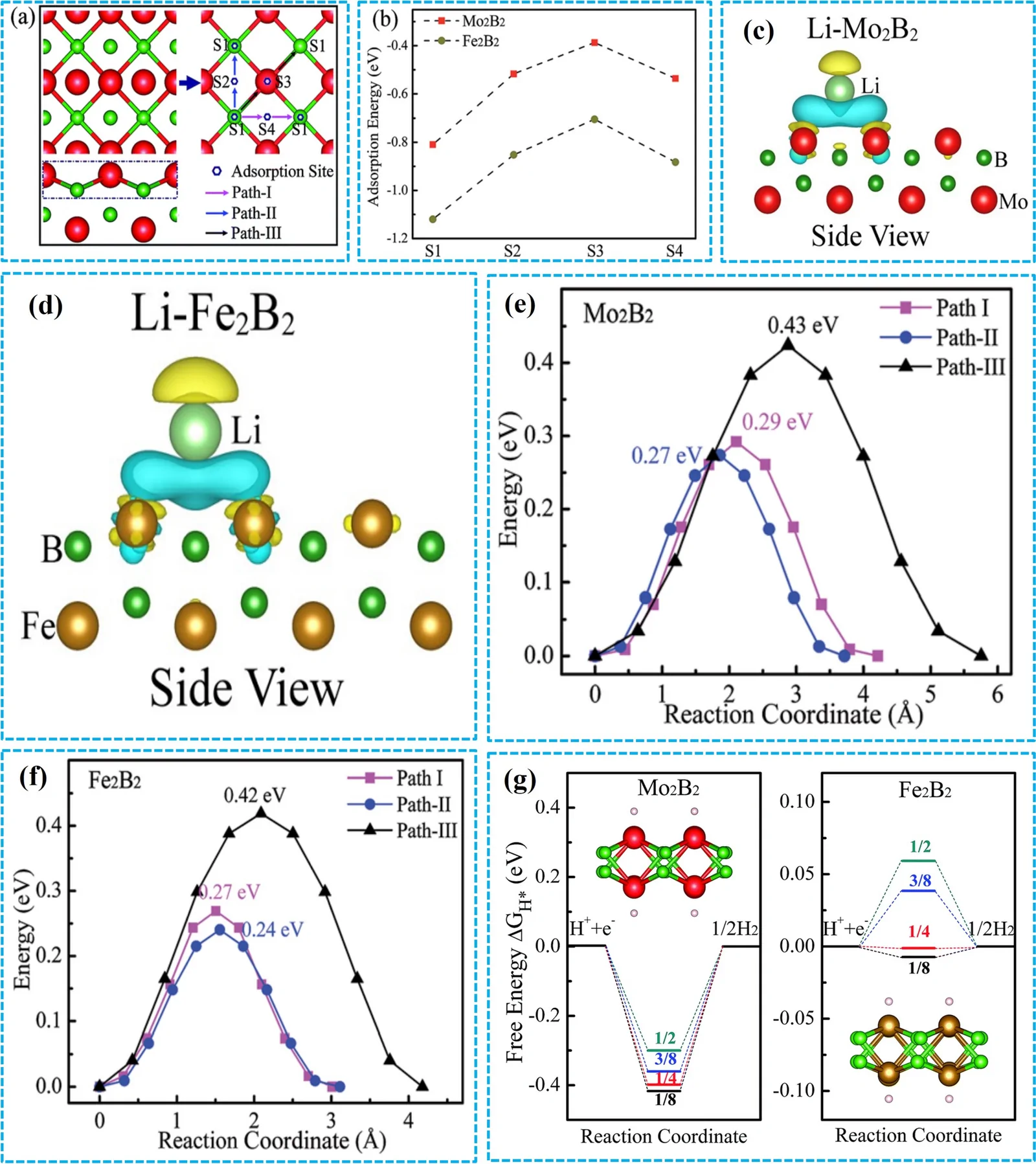
**a** Diffusion and adsorption sites of the Li atoms on MBene, where M (green) and B (green); **b** Adsorption energy of Li from S1-S4. **c–d** Charge energy difference of Mo_2_B_2_ and Fe_2_B_2_ MBenes after Li adsorption. **e–f** Diffusion energy profiles for lithium atoms on two-dimensional Mo_2_B_2_ and Fe_2_B_2_. **g** Premeditated free–energy illustration of the HER at the equilibrium potential (modified with permission [24])

Wang et al. [88] utilized the computational technique to synthesize the to investigate the charge storage capacity of the ScTiB_2_ MBene; it possesses good theoretical capacity up to 937 mAh g^−1^. The adsorption of the Li ions on the Sc interface was more stable than their adsorption on the Ti interface. The results of the positive E3 layer align with the outermost lithium atoms of ScTiB_2_Li_6_, which exhibit a propensity to aggregate into metal clusters, indicating that the monolayer has relinquished control over the outermost adatoms. The hex-ScTiB_2_ monolayer remained nearly intact throughout the Li atomic layer adsorption process, suggesting that the monolayer serves as a sufficiently stable anode material for the Li-ion insertion and extraction operations. The band gap, surface functionality, and distance between the M–B layer play a primary role in altering the electrochemical performance of the MBene. The morphology of the MBene is a very effective parameter, and the charge storage capacity of the porous and hetero surface MBene is better than the non-porous and flat MBene because porous and hetero MBene possesses extra active sites at the surface of the MBene and better interaction at with solvent. The pristine material always has poor charge storage capacity as compared to their composite because of the lack of synergistic effect; the synergistic effect can be generated by the addition of the material that possesses good active sites, stability, and conductivity to transfer their impact into the MBene and improve its performance, and cyclability. These properties of the MBene are associative with the synthesizing routes. The MBene synthesizing route generates a large fringe gap on the interface of MBene, affecting practical functionalities and Al removal capacity. MBene possessed ideal charge storage capacity in energy storage devices like batteries and SCs. Guo et al. [24] employed the simulation-based investigation to predict the 2D Mo_2_B_2_ and Fe_2_B_2_ properties as anode material for LIBs. The adsorption energy of each Li atom was evaluated in their computations. The DFT analysis shows that the MBenes possess four adsorption sites presented as S_1_–S_4_, as shown in Fig. 9a. The lowest adsorption energy of the Li atom at 2D Mo_2_B_2_ and Fe_2_B_2_ at the S1 site was observed at − 0.81 and − 1.12, respectively**.** The adsorption energy of the MBenes at four S sites is presented in Fig. 9b. The highest negative value of the Li adsorption shows that the MBenes interact well with Li ions to adsorb on their interface, enhancing the protection and reversibility of LIBs while helping to avoid the production of metallic Li. Additionally, the valuable charge energy difference appeared in MXene by the migration of the charge from Li to MBenes, as presented in Fig. 9c, d**.** Path-I and Path-II have very similar diffusion energy restrictions, suggesting they might be suitable paths for Li-ion diffusion, which boosts LIB charge–discharge rates. Path-I and II have substantially lower diffusion energy barriers than Path-III for 2D Mo_2_B_2_ and Fe_2_B_2_. The diffusion of adsorbed Li atoms determines LIBs, the charge–discharge rate. The lowest diffusion energy obstacles for LIBs on 2D Mo_2_B_2_, Fe_2_B_2_, graphite, graphene, MoS_2,_ and Ti_3_C_2_ MXene are 0.27, 0.24, 0.4, 0.277, 0.22, and 0.28 eV, respectively. The detailed diffusion behavior of the Li ion over MBenes at different is presented in Fig. 9e-f. The very negative value of the lithium adsorption energy was an exciting discovery. This defines a strong connection between Li atoms and MBene, which provides considerable advantages. It increases the safety and reversibility of LIBs by facilitating the development of problematic Li–metal formations. The systematically determined the Δ*G*_H*_ with H* coverage from 1/8 to 1/2 with an increment of 1/8, which are illustrated in Fig. 9g. Their simulation-based study predicted the theoretical charge storage capacity of the Li ions over 2D Mo_2_B_2_ and Fe_2_B_2_ MBene-based cathodes up to 444 and 665 mAh g^−1^, respectively. Additionally, the two substances show comparable energy obstacles along their diffusion routes, which enhances LIB charge and discharge rates. Mo_2_B_2_ and Fe_2_B_2_ MBenes are excellent choices for anode components for LIBs because of their low diffusion energy obstacles, substantial LIB abilities, and broad applicability.

**Fig. 10:**
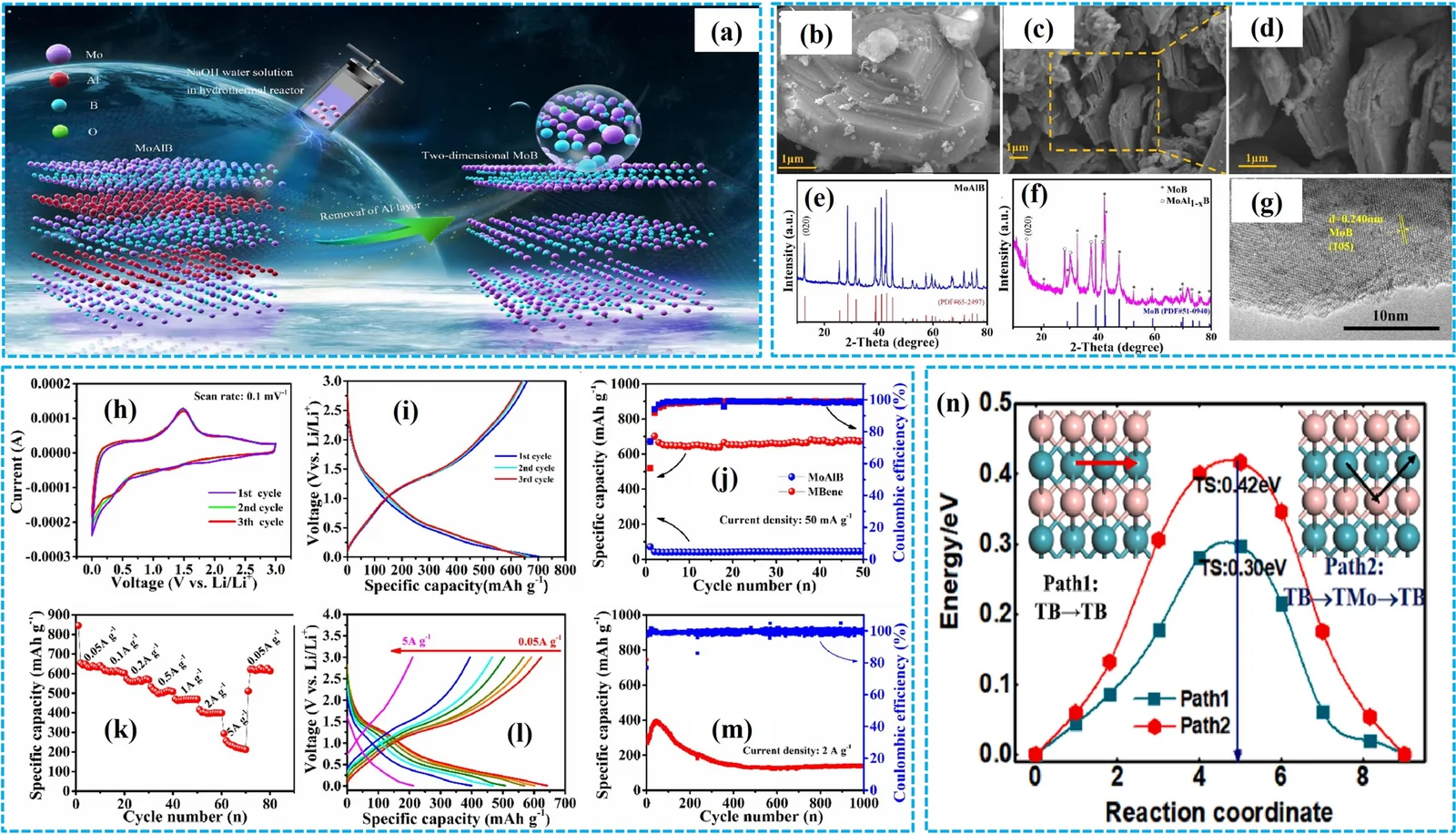
**a** Synthesizing process of the 2D MoB MBene with MoAlB and NaOH reaction. **b** SEM photos of MoAlB phase and **c-d** Their resulting etched MoB particles exhibit clear accordion-like layer structure; **e–f** MoAlB and 2D MoB MBene XRD spectra; and **g** TEM photo of 2D MoB MBene. **h-j** CV, GCD curves at 50 mAh g^−1^, and the cycle performance of MoAlB and 2D MoB MBene, **k-n** Rate capability, GCD and long cycling performance of 2D MoB MBene with their computed diffusion energy barriers for the two pathways; inset image illustrates the diffusion routes for lithium atoms. The green, purple, and undertone atoms represent B, Mo, and Li atoms, respectively (adapted with permission [111])

Xiong et al. [111] utilized the toxic fluorine-free alkane (NaOH) solution-based hydrothermal-assisted technique to eliminate the Al layer from MoAlB to synthesize the pristine 2D MoB MBene-based anode material for the LIBs. The graphical representation of the synthesizing process of the 2D MoB MBene by eliminating the Al layered form parent material MoAlB by NaOH solution-based hydrothermal-assisted technique is presented in Fig. 10a. The MBene-based anode was synthesized by combining MoB, super P carbon black, and carboxymethyl cellulose in weight percentages of 80%, 10%, and 10%, respectively, in deionized water. The lamellar-staking structure and standard X-ray diffraction (XRD) pattern of pristine MoAlB represented scanning electron microscopy (SEM) and XRD pattern in Fig. 10b, e, respectively. The pure MoAlB diffraction peaks matched the MAX phase’s standard XRD pattern. Figure 10c, d shows a similar structure of the synthesized MoB MBene to that of layered MXene materials. The XRD peak shows the practical and straightforward synthesizing of the pristine MoB MBene. However, some quantity of Al still existed in the MoB. The peak (020) of synthesized pristine MoB MBene at (Fig. 10f**)** 14.9° moved right relative to the MoAlB 12.6°, as demonstrated in Fig. 10e. Such alteration indicated the effective etching of A and the transformation of MAB into pristine MoB MBene. The pristine extensive crystalline MoB MBene possessed a lattice distance of 0.24 nm between their fringes, as presented in Fig. 10g by the HRTEM result. The synthesized MoB MBene-based anode possessed a magical specific capacity of up to 144.2 mAh g^−1^ at 2 A g^−1^ after 1000 cycles. They also compared the performance of the pristine MoAlB and MoB MBene as anode material. The specific capacity of the pristine MoAlB is almost zero at 50 mA g^−1^. Still, MBene possessed 671.6 mAh g^−1^ after 50 working cycles. These results show that eliminating the Al layer effectively improves the specific capacity of the MBene because of the enhancement of the specific surface area (SSA). According to the BET results, the SSA of the MoAlB and MoB is 0.48 and 28.1 m^2^ g^−1^, respectively. The detailed electrochemical performance of the synthesized MBene is presented in Fig. 10h-m. Another reason for enhancing the specific capacity is to improve the band gap between the fringes of the MoB sheets by eliminating the Al layer. In general, band gap (Ti–T), thickness, and lattice constantly increase by increasing the atomic number in the same group. The electron localization function (ELF) was computed to examine the bonding character, with ELF values more than 0.5 signifying covalent bonding and values less than 0.5 indicating ionic bonding. Bader charge study indicated a charge transfer of 0.8 e from Li ions to MoB layers. Li migration was examined along two diffusion pathways (Fig. 10n): Path 1, from a top B atom to an adjacent B atom, and Path 2, from a top B atom through Mo to an adjacent B atom. The energy barriers were 0.30 and 0.42 eV respectively, with Path 1 being more advantageous. For example, the specific thickness of the Ti_2_BF_2_, Ti_2_BI_2,_ and Ti_2_BTe_2_ are 4.892, 6.118, and 6.497 Å, respectively [87]. Wang et al. [112] reported the influence of the functionalization on the LIBs. They observe that the S- and Se-functionalized Ti_2_B MBenes possess enchanted electrochemical performance compared to the unfunctionalized Ti_2_B MBenes.

### MBene-based Li–S Batteries

LSBs are one of the capable types of rechargeable devices. The higher energy density and potential make it an ideal energy storage device compared to the traditional LIBs. However, their advancement is impeded by several hurdles, including issues like limited conductivity in sulfur cathodes, the orbital effect, and the gradual decomposition of Li_2_S groups [113]. The shuttle effect refers to the process occurring during the charging and discharging of a battery: The polysulfide (Li_2_S_*x*_) intermediate generated by the positive electrode disintegrates in the electrolyte, migrates through the separator, flows to the negative electrode, and subsequently reacts with the lithium metal present. This results in the permanent depletion of active materials in the battery diminishes its lifespan, and decreases its coulombic efficiency. The shuttle effect can occur when soluble lithium polysulfides (LiPS) are present. This action can have adverse effects on several different components that are contained within the system.

**Fig. 11:**
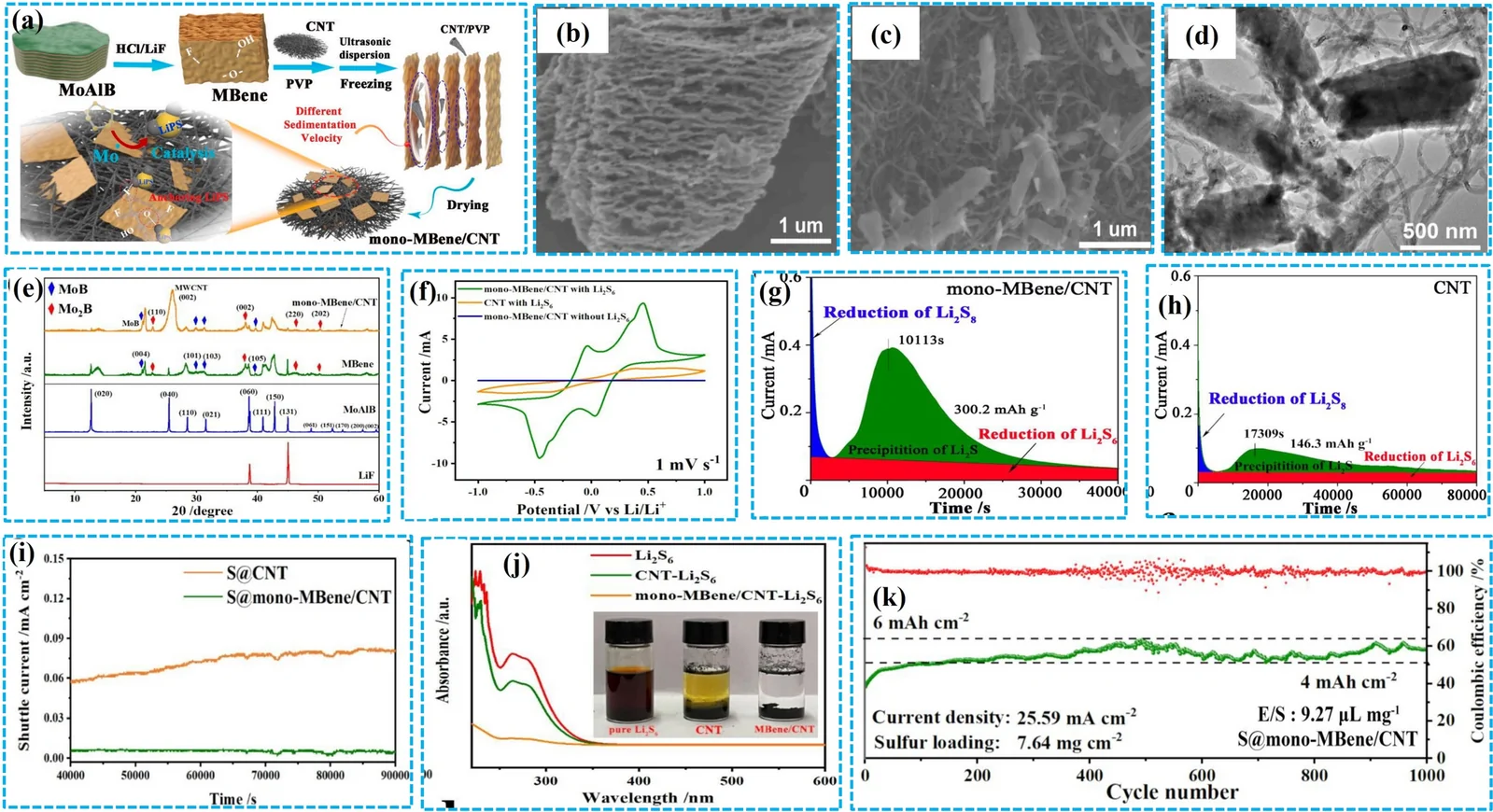
**a** Synthesis of the MBene/CNTs. SEM images of **b** MBene, **c** CNTs/Mbene. **d** TEM images of CNTs/Mbene. **e** XRD analysis of the materials. **f** CV curve. The Li_2_S nucleation test with **g** mono-MBene/CNT, **h** CNT, **i** S@mono-MBene/CNT cathode and S@CNT cathode comparison. **j** Graphic absorption test of Li_2_S_6_ and polysulfide through UV–Vis analysis for mono-MBene/CNT and CNT. **k** Columbic efficiency of high S loading cells with S@mono-MBene/CNT cathode (modified with permission [115])

Li et al. [114] conducted the first study to utilize the HCl/LiF solution and ice template technique to synthesize the MoB by eliminating the Al layer from MoAlB and MBene and CNTs composite-based cathode in Li–S ion batteries. The detailed synthesized process of the MBene/CNT-based composite electrode material for LSBs is presented in Fig. 11a. The existence of the –F, -OH, and –O functional groups on the surface of MBene facilitates the formation of the composite. Unfortunately, the poor conductivity of sulfur up to 5 × 10^–30^ S cm^−1^ and their volume enhancement during the long cyclability and shuttle impact are the primary drawbacks that still limit the commercialized use of the Li–S batteries. In all these factors, the shuttle effect is the crucial parameter and the most viable obstacle in developing and commercializing Li–S batteries because it is responsible for massive self-discharge, rapid capacity degradation, and low coulomb efficiency. Numerous attempts were made to address these problems, mainly by lowering the shuttle effect by attaching lithium polysulfides (LiPS). Materials made of porous carbon, advantageous because of their high porosity and excellent conductivity, are frequently employed as a substrate to load sulfur. Limited capacity and poor cycle reliability are the outcomes of the extreme difficulty of nonpolar carbon in using the intermolecular force to secure the polar LiPS.

Additionally, doping the heteroatoms, including N, O, and F, on carbon material enhanced the interaction of the electrode material with electrolytes to enhance Li–S berries’ electrochemical performance. These modifications limit the conversion of Li–S batteries or increase the stability during charging and discharging. Unfortunately, massive loading of these materials limits the number of active sites, resulting in poor redox kinetics. So, theoretical studies proved that MBene possessed dual advantages of 2D materials and metal boride availability to improve the stability or limit the degradation rate and improve the electrochemical efficacy of the Li–S batteries. In MBene/CNTs composite, CNT is responsible for enhancing the conductivity of the cathode. The massive active sites in the MBene provide extra active sites that can facilitate the conversion of LiPS compared to multilayer structures. The SEM procedure was used to examine the surface and structure of the MBenes before and after blending with CNTs. The pristine MBene possesses a multilayered structure (Fig. 11b). When mixed with CNTs, the multilayered structure converts into a monolayered MBene (Fig. 11c, d). The XRD analysis investigated the crystalline assembly of the CNTs/MBenes (Fig. 11e). Researchers performed CV tests utilizing asymmetrical cells fitted to various cathodes to evaluate the impact of *mono*-MBene on the catalytic conversion capabilities of LiPS.

The results of these tests are depicted in Fig. 11f. Based on these findings, it can be deduced that the active effect caused by Mo atoms is responsible for the increased speed of redox processes in *mono*-MBene [116, 117]. In addition, the mono-MBene nanosheet architecture provides a substantial amount of SSA, making it an ideal habitat for sulfur vulcanization processes. This increase in redox kinetics, which can be attributable to the incorporation of mono-MBene into the cathode, is further supported by a Li_2_S nucleation test that depends on Faraday’s law [118, 119]. The Li_2_S_2_ precipitation of the *mono*-MBene/CNT and CNT is 300.2 and 146.3 mAh g^−1^ (Fig. 11g, h). These findings show that the mono-MBene/CNT is valuable for the nucleation of the Li_2_S_2_. The shuttle effect of the S@mono-MBene/CNTs cathode is inferior to the S@CNTs cathode, as shown in Fig. 11i. These results show that the mano-MBene has a worthwhile influence on reducing the shuttle effect. This phenomenon can be attributed to the solid binding action of mono-MBene on F, O, and B atoms in LiPSs. To more accurately assess the impact of mono-MBene on LiPS anchoring, an optical experiment was conducted using optically clear bottles. Equal quantities of carbon nanotubes and mono-MBene/CNT have been added to a Li_2_S_6_ solution with a dosage of 20 mmol/l, using the same weights. As seen in Fig. 11j, the undiluted Li_2_S_6_ solution first displays a reddish-brown color and retains this shade after 12 h of remaining stationary. Significantly, the Li_2_S_6_ solution, including mono-MBene/CNT, loses color within 12 h. In contrast, Li_2_S_6_ solutions containing CNT additions exhibit a faint yellow coloration over 12 h of standing. The results validate that monolayer MBene has a more vital ability to adsorb Li_2_S_6_ than CNT. This is due to the strong affinity of the F, O, and B atoms in the mono-MBene for LiPS, which allows for effective anchoring. Prior studies have confirmed that B atoms significantly impact anchoring LiPS.

There was a significant difference in peak currents between symmetrical cells with *mono*-MBene/CNT cathodes and those with CNT cathodes when the scan rate was 1 mV s^−1^. This difference indicates that the redox kinetics of the cells were faster. The monolayered MBene/CNTs material decomposition was approximately zero at 600 °C. These findings demonstrate that the synthesized composite has good thermal stability and is unlikely to decompose following molten sulfur injection. The existence of the –F and –O atoms on the interface of the MBene is beneficial for the MBene/CNT composite’s magical power to adsorb the LiPS. The synthesized S-MBene/CNT cathode possessed an initial aerial capacity of up to 12.73 mAh cm^−2^. It maintains its aerial and retention capacity up to 11.07 mAh cm^−2^ and 86.96% after 120 charging and discharging cycles, which is approximately three times higher than the capacity of commercial lithium-ion batteries. It possesses magical long-term cycling efficiency after 1000 working cycles up to the 15.28 mg cm^−2^ loading of the sulfur and lean electrolyte (Fig. 11k). The various parameters enhance the electrochemical performance of the S-MBene/CNT cathode. First is the significant SSA of CNTs and 2D MBene multilayered structure, and the electron-deficient nature of the boron is responsible for the massive and easy adsorption of the polysulfide anion, which assists the transport of lithium ions. Second, the Mo atom increases the conversion rate among LiPS because of its catalytic feature. Thirdly, LiPS may benefit from a robust binding force provided by the many fluorine, oxygen, and boron atoms on the outermost layer of MBene, significantly lessening the shuttle effect. So, the MBene/CNTs cathodes effectively reduce the shuttle effect because of the speed catalysis and MBene chemical adsorption. The detailed electrochemical analysis of the material possesses, in addition to offering a novel sulfur host that can lessen the shuttle effect and improve LiPS conversions for Li–S batteries, this research could serve as a model for using MBene in the energy storage industry. Xiao et al. [120] carried out the DFT calculation to investigate the impact of the functionalization of the MBene on the reduction of the shuttling effect in LSBs. Their findings indicate that the unfunctionalized Mo_2_B_2_ MBene has a greater rate of LiPS breakdown due to the intense binding energy between S and the outer layer of the Mo atoms. The MBene’s functionalization slowed the LiPS breakdown in SIBS batteries. Additionally, the functionalization of the MBene reduced the shuttle effect in LSBs.

### MBene-based Na-ion Batteries

**Fig. 12:**
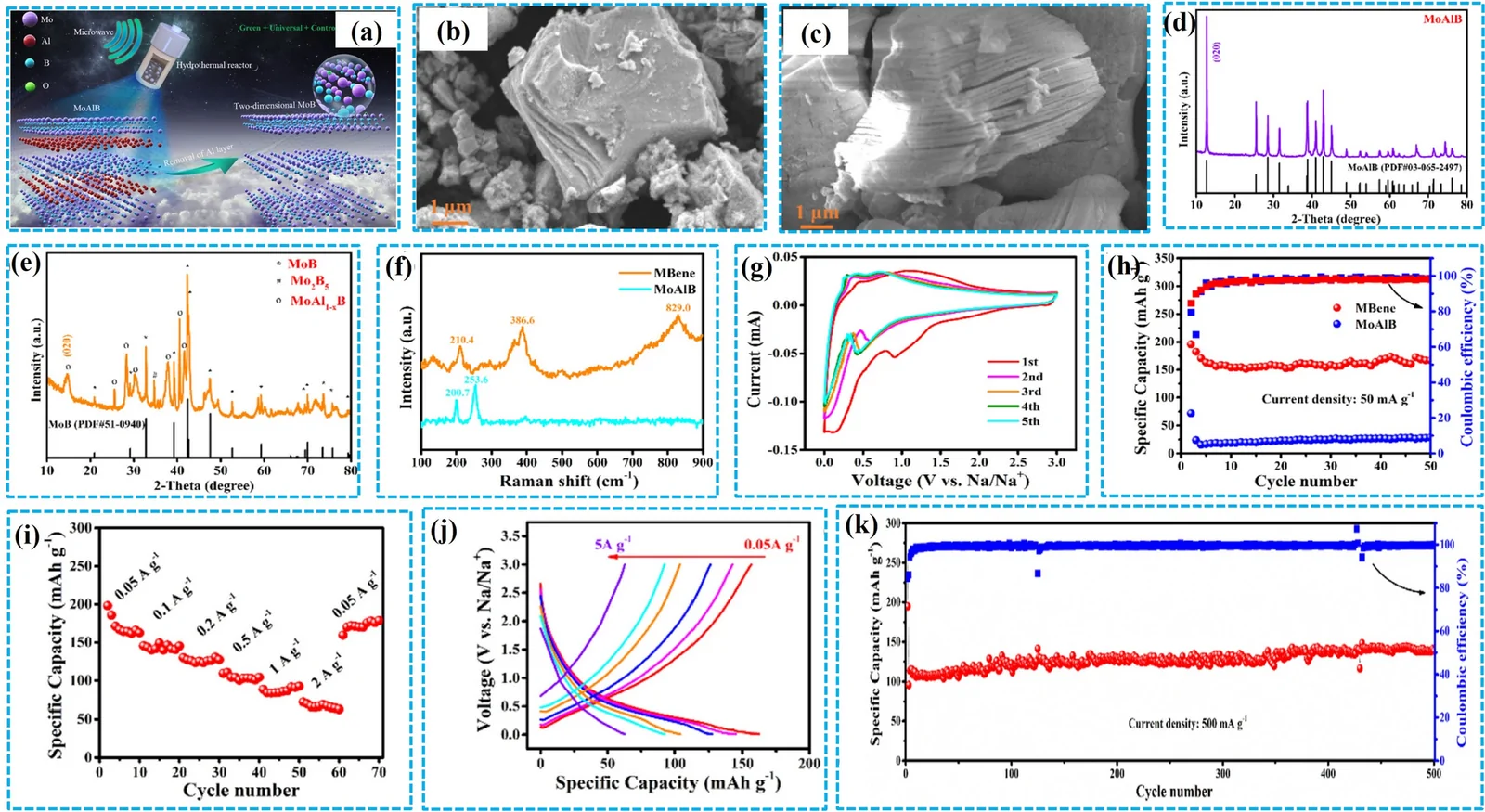
**a** Synthesizing process of MoB MBene, SEM images of **b** MoAlB, **c** MoB. XRD peaks of **d** MoAlB, **e** 2D MoB Mbenes. **f** Raman spectra of MoAlB and MBene, Electrochemical analysis of the MBene, **g** CV curve, at 0.1 mV s^–1^. **h** Cycle performance of MoAlB and MoB MBene at 50 Ma g^–1^. **i** Specific capacities of MoB MBenes at different current densities, **j** The corresponding voltage curves of MoB Mbenes. **k** Cycling stability and coulombic efficiency of MoB MBenes at 500 mA g^–1^ (adapted with permission [124])

With the abundance and limited cost of sodium resources, SIBs are a promising replacement for LIBs. Although SIB technology is comparable to LIBs, there are notable distinctions between the behavior of Na^+^ ions and that of Li^+^ ions. These distinctions have an impact on the design of these batteries as well as the materials that are utilized in their construction [121, 122]. However, it has been shown that Na ions have little detectable effect when readily accessible graphite is used as an electrode material in SIBs [123]. This circumstance highlights the urgent need for innovative anode materials to improve SIB efficiency. Xioang et al. [124] synthesized the MoB MBene by dissolving MoAlB powder into a specific volume of NaOH in an autoclave using a microwave-assisted hydrothermal machine. Figure 12a shows the whole synthesis procedure for MoB MBene from MoAlB*.* SEM pictures of the MoAlB and MoB MBene show lamellar-stacking structural and accordion-like layer structures similar to MXene, as shown in Fig. 12b, c, respectively; there is a coincidence between the diffraction peak values of the pure MAB phase and the MoAlB standard XRD pattern, as given in Fig. 12d. The XRD peaks show that the MoAlB MBene is formed. On the other hand, it is observable that there is still some MoAlB there, which gives the impression that the Al atoms have not been eliminated. When contrasted with MoAlB’s original state (Fig. 12e). The Raman spectra of MoAlB possess peaks at 200.7 and 253.6 cm^−1^; these peaks shift 210.4 and 386.6 cm^–1^, and a broad peak centered at 829.0 cm^–1^ appears in the MoB MBene after the elimination of Al layer (Fig. 12f). The electrochemical performance of the MoB-based MBene anode material in SIBs is presented in Fig. 12g–k. The BET calculation was used to investigate the impact of the acid etching on the SSA of MBene. The SSA of the MoAlB and MBene are 5.9 and 28.1 m^2^ g^–1^, respectively. So, the acid etching has a valuable effect on the enhancement of the SSA of the MBene. As compared to the MoAlB, MBene possesses 5.5 times more SSA. In SIBs, the MBene-based anode material possesses a charge storage capacity of up to 196.5 mAh g^–1^ at 50 mA g^–1^ and 138.6 mAh g^–1^ after 500 cycles at 0.5 A g^–1^. The charge storage capacity of the synthesized MBene-based anode material in SIBs is better than the many reported MXenes. Mathematical calculations were carried out to uncover its mechanism, and the process’s structural inequality and electrical and thermal aspects were investigated. Researchers hope that the enhanced technology will ease the process of producing high-quality MBene materials, eventually making it easier to examine the possibility of using these materials in various metal-ion batteries of tremendous potential.

Liang et al. [125] employed the DFT computational analysis technique to explore the N-functionalized Ti_2_B MBene-based anode material in SIBs. The pristine MBen-based electrode material generally owns a lower specific capacity than the functionalized Ti_2_B MBene-based anode material; the theoretical charge storage capacity of the MBene-based anode material for SIBs is up to 797 mAh g^−1^. Ti_2_BN_2_ has a low diffusion resistance of 0.34 eV and a reasonable open-circuit voltage of 0.27 V, demonstrating that it is an excellent choice for the anode material in SIBs. Gao et al. [126] employed the theoretical DFT calculation to investigate the charge storage mechanism and capacity of the Y_2_B_2_ MBene-based anode material for SIBs. The synthesized anode material possesses adsorption sites S1, S2, and S3, and the Na metal is adsorbed at S2 and S3 adsorption sites. The adsorption energy of the Na at S1 and S3 is − 0.449 and − 0.441 eV, respectively. The sites’ highest negative value shows that Na is readily adsorbed.

In conclusion, the dynamical and thermal characteristics, electron conduct, and possible uses of 2D Y_2_B_2_ MBene as anode material in recharging SIBs were investigated methodically using first-principles computations. After these calculations, it was shown that the lowest diffusion energy barrier for sodium ions on the Y_2_B_2_ MBene sheet is 0.008 eV. When the temperature is 300 degrees Kelvin, the diffusivity of sodium can reach as high as 0.013 cm^2^ s^−1^. This indicates the capacity to charge and discharge 2D Y_2_B_2_ MBene quickly, as the anode in batteries is of excellent quality. On 2D Y_2_B_2_ MBene, the specific capacitance of sodium is reached 403.16 mAh g^−1^. Variations in the open-circuit voltage (OCV) ranges for sodium with varying quantities of sodium range from 0.45 to 0.15 V, respectively. 2D Y_2_B_2_ MBene possesses remarkable physical characteristics, indicating a promising battery potential. At the end of this section, we can say that theoretical and experimental investigation on the MBene-based anode material for SIBs proved that MBenes have magical potential to solve the charging and stability issue of the traditional 2D material-based anode material issues of the stability. The diffusion resistance is an essential feature of the electrode materials because it controls the pace at which metal ions move during the charge and discharge processes [127]. When the diffusion barrier is reduced, metal ions can diffuse more quickly [114]. As a result, once they have identified the most stable adsorption site for metal ions, they investigate the diffusion barrier between this stable site and other adsorption sites. First, the authors placed the procedure’s start and final states in their appropriate placements. The CI-NEB method was subsequently employed to calculate the diffusion pathway and the corresponding barrier. Based on the system’s symmetry, the potential trajectory may have three consecutive segments, designated as S1–S3–S1 internally. The Na ions were found at the S2 location throughout the experiment. The energy required for the adsorption of sodium ions at site S2 is comparable to that needed at site S3. There was a rapid diffusion of sodium ions toward S2–S3–S2. The computed diffusion barrier was shallow, with a value of approximately 0.011 eV. Because this barrier was relatively low, it was determined that V_2_B_2_ possesses excellent potential for anode material usage.

### MBene-based Mg-ion Batteries

**Fig. 13:**
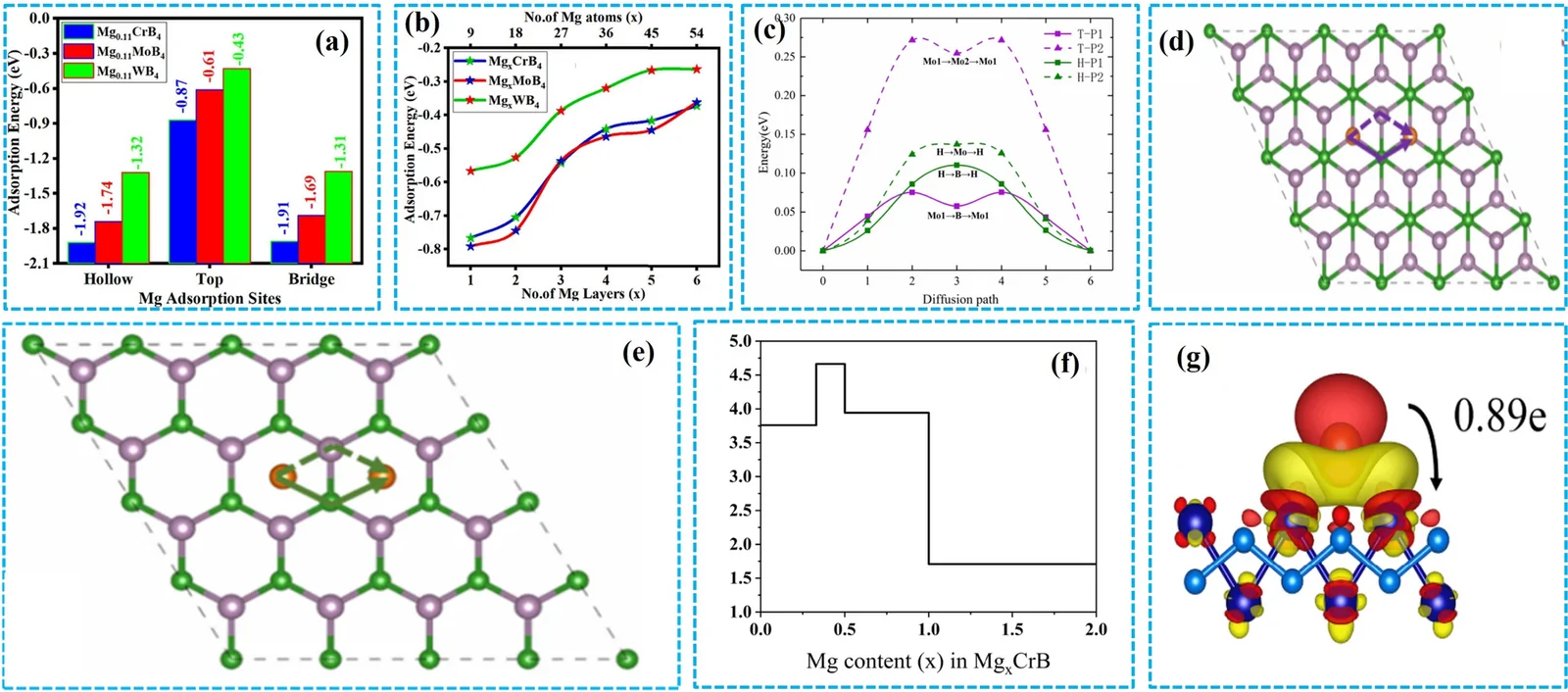
**a-b** Surfaces of CrB_4_, MoB_4_, and WB_4_, as well as the adsorption energies of magnesium atoms; **c-e** Ultralow diffusion obstacles of Mg atoms on their outermost surfaces (improved with permission [128]). **f** OCV for CrB in adsorption at various concentrations of Mg atoms. **g** Charge density difference plots for Mg/CrB, the red and blue colors resemble to charge gathering and reduction (improved with permission [130])

Magnesium ion batteries (MIBs) are a novel rechargeable battery technology that uses magnesium ions as charge carriers. Magnesium possesses a superior volumetric capacity compared to lithium, possibly enabling batteries with enhanced energy density. Rechargeable MIBs have deposited important courtesy recently due to their favorable attributes, including their plentiful accessibility, environmental stability, affordability, and friendliness. Energy storage in MIBs, which rely on electrochemical processes involving the movement of electrons and ions, is of significant interest to researchers. In energy storage devices’ charging and discharging process, electrons are stored and produced to supply power to external devices [115]. Consequently, the capacity for reversible energy storage in MIBs is essentially determined by the number of electrons exchanged, the capability of materials to preserve their structure during ion intercalation and deintercalation, and the rate at which the electrolyte regulates the diffusion of ions. Therefore, the battery’s characteristics of both the electrode material and the electrolyte will determine efficiency. The primary hurdle the Mg ion batteries face is the robust polarization of the Mg ions with traditional electrode materials, making it challenging to capture Mg ions in such electrode material. MBene is a promising material for fixing such obstacles in Mg-ion batteries. Masood et al. [128] employed the DFT calculation to analyze the 2D monolayers of CrB_4_, MoB_4_, and WB_4_, MBene energy storage behavior in MIBs as anode material. Their DFT findings show that the synthesized MBene possesses excellent structural, mechanical, and thermal stability. The highly negative adsorption energy is crucial for stabilizing the Mg ions on the surface, stopping them from clumping, and keeping MIBs stable. The surfaces of CrB_4_, MoB_4_, and WB_4_, as well as the adsorption energies of magnesium atoms, are observed, as presented in Fig. 13a, b. A comparison of the adsorption energies of magnesium metal atoms were carried out to ascertain which adsorption site is optimal for magnesium ions. A negative sign is present in every single computed Eads value that indicates exothermic adsorption. This is a characteristic that frequently leads to the creation of metallic clusters. Compared to the B site and the T site, the H site is more stable regarding the adsorption of magnesium atoms on the surfaces of CrB_4_, MoB_4_, and WB_4_. Because of this, the H site was selected for the subsequent adsorption of magnesium ions. During the adsorption process, the metallicity is kept in excellent condition. Exceptional stability during the magnification process demonstrates its potential for practical use in sophisticated battery systems. The maximum charge capacity and OCV of the CrB_4_, MoB_4_, and WB_4_ are 3377, 2311, 1416 mAh g^−1^ and 0.27, 0.28, and 0.19 V, respectively. These results show that the synthesized MBene-based anode material suits the MIBs.

Mei et al. [129] utilized the DFT calculation to investigate the T-type and H-type Mo2B MBene as anode materials for MIBs. The simulation findings demonstrate that the energies of adsorption of T-type and H-type Mo_2_B MBene for Mg atoms are − 1.08 and − 0.78 eV, respectively. These energies are enough to keep the magnetization process stable. Figure 13c–e illustrates the ultralow diffusion obstacles of magnesium atoms on their outermost surfaces, which exhibit an acceptable charge and discharge efficiency; the specific capacity of T-type and H-type Mo_2_B monolayers and their theoretical voltages. The results of this study suggest that T-type and H-type Mo_2_B monolayers can be used as anode materials for MIBs. Similarly, Wang et al. [130] employed the DFT calculation to examine the adsorption behavior of the Mg ions over CrB, FeB, and MnB 2D monolayer MBenes as an anode material in MIBs. The findings indicate that the single-layered MBenes have low barriers to diffusion and can keep an average OCV that is steady, as shown in Fig. 13f, and Mg ion adsorption capacity for CrB, FeB, and MnB 2D monolayer MBenes as an anode material in MIBs are 426, 402, and 407 mAh g^−1^, respectively. Based on these characteristics, monolayer CrB, FeB, and MnB can potentially be anode materials for MIBs with exceptional rate capacity. Figure 13g displays the changes in charge density of the three most stable adsorption systems containing CrB, FeB, and MnB monolayers. As we can see in the picture, electrons build up between the metal ions and the B atoms next to them. This makes a chemical bond that stops metal groups from forming. Additionally, the Mg atoms shipped to the monolayer CrB, FeB, and MnB are 0.89, 1.08, and 0.98 e, respectively. The number of charges that pass shows how much power is being absorbed. Because of this, there is a strong link between the metal atoms and the shape of the surface. That makes it easier for particles to stick to the top of things, which creates more space.

Chotsawat et al. [131] employed the DFT calculation to investigate the charge storage and diffusion behavior of the Mo_2_B MBene-based anode material for the MIBs. Researchers investigate the structural inequality, electrical, and electrochemical aspects of both H- and T-type geometries of Mo_2_B. The findings show that Mo_2_B shows metallic behavior and outstanding metal adsorption qualities, with Mg displaying the highest affinity for binding. As a consequence of this strong binding, particularly with magnesium, more considerable diffusion barriers are produced, improving layer adsorption and theoretical capacity. The computed optimum capacities are statistically significant, with 923 mAh g^−1^ being the greatest. The OCV for Mg falls within the range of 0.1–1.0 V, indicating that Mo_2_B MBene is a good candidate for anode materials that reduce the probability of dendrite development. Based on the research results, Mo_2_B MBene is excellent as an anode material for next-generation metal-ion batteries due to its high capacity, voltage, and safety. These results might be helpful in the future experimental expansion of anodes based on Mo_2_B for energy storage devices.

### MBene-based Ca-ion Batteries

**Fig. 14:**
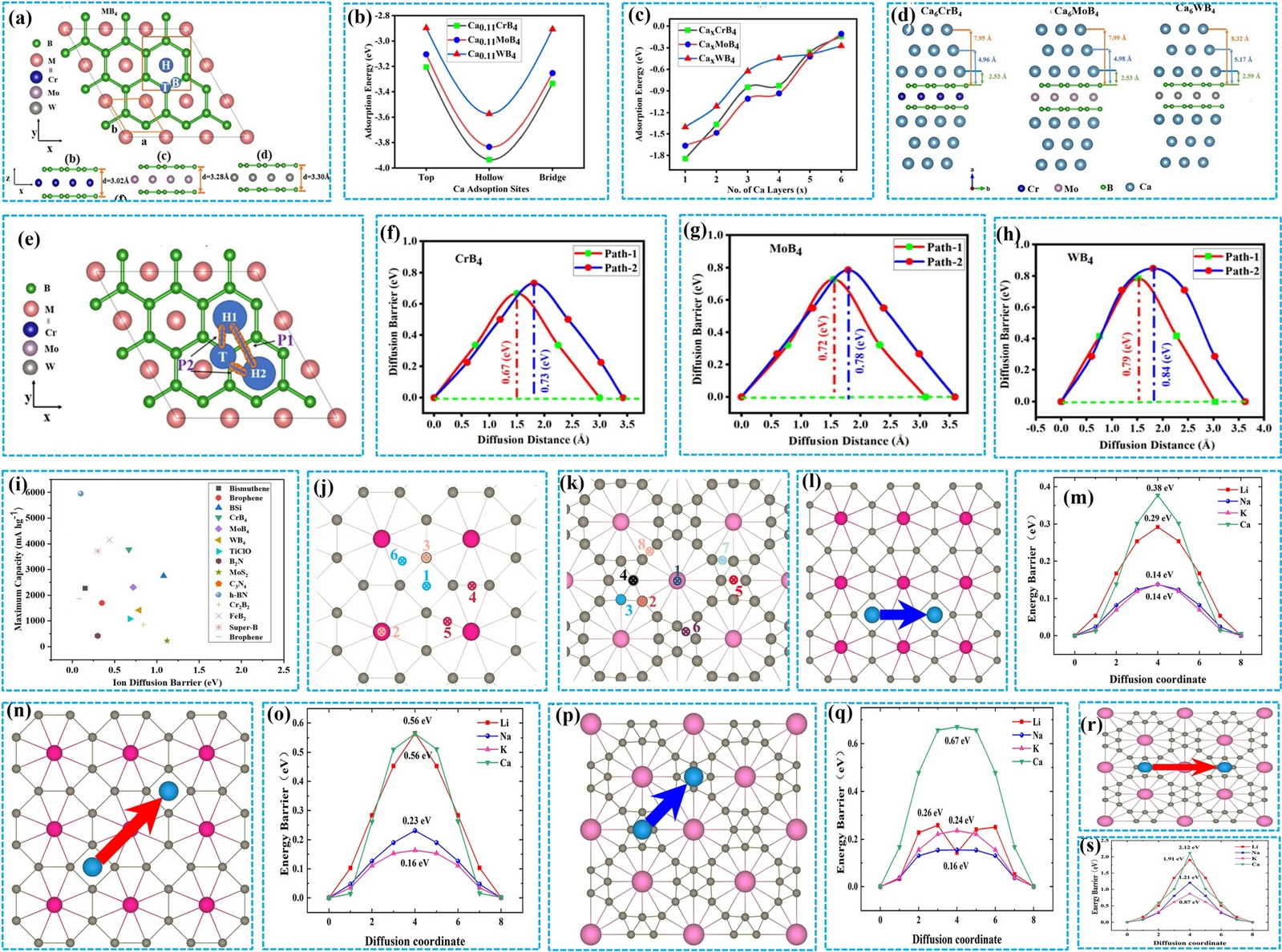
CrB_4_-, MoB_4_- and WB_4_-based anode materials: **a** Adsorption sites, **b** Adsorption energies of the stable Ca^2+^ ions adsorption sites, **c** Impact of the Ca^2+^ ions adsorption layers on the adsorption energies next Ca.^2+^ ions. **d** Comparative analysis of the interlayer spacing between the adsorbed Ca-ion layers on MBene-based anode. **e** Migration path 1 (H1–H2) and path 2 (H1–T–H_2_) for Ca ions on the MB_4_ monolayer. **f–h** Diffusion energy barriers for Ca-ion diffusion. **i** Comparative analysis of the ion diffusion barrier and maximum storage capacity with the other reported anode materials (adapted with permission [63]). The top view of the adsorption sites on **j** TiB_4_ MBene Sites T1-T6, **k** SrB_8_ MBene adsorption sites S1-S8. **l** Migration path of the charges over TiB_4_ Mbene. **m** Energy barriers for the diffusion of charges over TiB_4_ Mbene. **n** Migration path of the charges over TiB_4_ Mbene. **o** The diffusion barrier profile of charges over TiB_4_ Mbene. **p** Migration path of the charges over SrB_8_ Mbene. **q** Energy barriers for the diffusion of charges over SrB_8_ Mbene. **r** Migration path of the charges over SrB_8_ Mbene. **s** The diffusion barrier profile of charges over SrB_8_ MBene (adapted with permission [133])

The CIBs are a promising class of rechargeable batteries. In CIBs, Ca^2+^ ions are the charge carriers. Because of the abundant supply of calcium, the inexpensive nature of CIBs, and their capacity to offer exceptionally high energy densities, CIBs are attracting much attention as a possible alternative to LIBs. The divalent charge on the interface of the calcium shows that each calcium ion carried out two positive charges, which means it possesses higher charge densities than the Li^+^. The CIBs are a better choice as compared to the LIBs because of (a) their divalent nature, which encourages more powerful bonding and the retention of one electron for lithium and two electrons for each Ca atom, which ultimately leads to a rise in both the theoretical volume and the specific capacity, respectively. (b) No dendritic growth issues can occur in metallic lithium, which means that calcium can be electrodeposited without any interference, and (c) the smaller radius of the Ca^2+^ ion responsible for the rapid diffusion of the Ca^2+^ than Li^2+^ [132]. Masood et al. [63] introduced the DFT to investigate the charge storage capacity and Ca^2+^ ion diffusion barrier over CrB_4_, MoB_4,_ and WB_4_ monolayer MBene-based anode material for CIBs. The energy surfaces of the MoB_4_, CrB_4_, and WB_4_ monolayers select how Ca sticks to the MB_4_ monolayer. Figure 14a, b shows the adsorption energy of the Ca atom on the three different adsorption sites of the monolayer MB_4_ MBenes. The hollow site (H site), the bridge site (B site), and the top site (T site) are these. It takes − 3.94, − 3.18, and − 2.74 eV for the Ca atom to bind to the MBene monolayer’s hollow sites; − 3.93, − 3.27, and − 2.69 eV for the B sites; and − 3.20, − 2.33, and − 1.89 eV for the T sites. There is much less absorption energy at the Hollow site than at the T and B sites for all three materials. This means the H site is probably the best place for the expected Ca atom to stick. The average binding energy of the calcium layers on the artificial MBene-based anode material is shown in Fig. 14c. The predicted MB_4_ MBene possesses excellent adsorption energy of Ca^2+^ on their MBene anode layers because of the solid negative adsorption energy. The enhancement of the distance between Ca layers and the MB_4_ monolayer is because of the enhancement of the adsorption of the Ca^2+^ layer over MBene. So, the adsorption energy for the third layer is less than the second. However, the adsorption energies remain negative, indicating a beneficial outcome. Adding further layers is impossible as this would result in the adsorption energies becoming positive. The Ca atoms and the substrate have a sufficiently strong connection to allow the adsorption of a monolayer of Ca atoms. The contacts decreased strength upon absorbing the 2nd and 3rd layers, resulting in weak coulombic interactions between the highest layer and the substrate.

Consequently, the adsorption energy experienced a decrease. The deposition of the layer of Ca atoms increases on the MBene, which is responsible for enhancing columbic repulsion between the following Ca-deposited atoms; this increases the repulsion force between Ca atoms and MBene-based anode. The adsorption energy decreases but stays negative due to the repulsion of the intralayer Ca atoms. The adsorption of the Ca over MB_4_ anode material increases the distance between MB_4_ layers and next-to-next adsorption of the Ca layers, as presented in Fig. 14d. These findings again verified that the MBene layered has lower adsorption energy for coming Ca^2+^ ions than the previously adsorbed Ca^2+^ layers. The mechanism of the migration of the Ca^2+^ ions over MBene is presented in Fig. 14e. The diffusion berries are an effective technique to investigate the active material batteries’ rate capability and cyclability. The energy barrier indicates the amount of energy needed for the diffusion process to take place. The diffusion barrier governs the movement of Ca ions between the MB_4_ compounds that form the battery’s electrode in Ca-ion batteries. During the charging and discharging process, diffusion barriers regulate the pace at which ions traverse a material. Simply put, the rate at which ions diffuse is directly proportional to the energy barrier’s decreasing value. Consequently, we can comprehend the structural uniformity necessary for finding the most effective diffusion path by locating the pathway with the lowest diffusion barrier. The outermost layer of WB_4_ has a higher diffusion barrier than the surface of CrB_4_ and MoB_4_, respectively. W has bigger atoms than Cr and Mo, which might change how Ca ions interact with the grid and make it harder for them to move around. The diffusion barriers for the adsorption of the Ca^2+^ ions over MBene are schematically presented in Fig. 14f–h. The comparative analysis of the MBene charge storage capacity of the MBene-based anode material with other 2D anode materials is graphically presented in Fig. 14i. The comparative analysis of MBene with other two-dimensional materials shows that MBene has exceptional charge storage capacity and diffusion barriers relative to the other 2D materials. The theoretical charge storage capacity of the CrB_4_, MoB_4,_ and WB_4_-based anode materials for CIBs are 3377, 1231, and 1416 mAh g^−1^, respectively, at low open-circuit voltage of 0.45, 0.47, and 0.35 V. These findings show that the MBene is a promising choice for the anode material in CIBs.

Wang et al. [133] employed the first-principles calculations to investigate the electrochemical performance of the TiB_4_ and SrB_8_ MBene-based anode material for CIBs. The theoretical Ca^2+^ ions charge storage capacity of the TiB_4_ and SrB_8_ MBene-based anode material for CIBs are 1176 and 616 mA h g^−1^, respectively. They also investigated the impact of the charge storage capacity of the TiB_4_ and SrB_8_ MBene-based anode material in LIB, SIBs, and PIBs. Their finding shows that the charge storage capacity of the TiB_4_ and SrB_8_ anode material for LIB, SIBs, and PIBs are 588, 588, 588, and 308, 308, 462, and 616 mAh g^−1^, respectively. These results show that TiB_4_ and SrB_8_ MBene-based anode material possess a promising Ca^2+^ ions charge storage capacity compared to the LIB, SIBs, and PIBs. The Ca^2+^ ions adsorption energy for the TiB_4_ MBene T1 to T6 adsorbent sites are − 0.646, 0.227, − 0.372, − 0.366, − 0.177, and − 0.215, all in eV, respectively, these findings show that the T1 is the effective sites for the adsorption of the Ca^2+^ ions, the visualization of the existence of the Ca^2+^ ions adsorption sites on TiB_4_ MBene from T1 to T6 are presented in Fig. 14j. Similarly, The Ca^2+^ ions adsorption energy for the SrB_8_ MBene S1 to S8 adsorbent sites is 1.748, 0.129, 0.112, − 0.010, − 0.169, 0.107, 0.103, and 0.137, respectively, these findings show that the S5 is the effective sites for the adsorption of the Ca^2+^ ions, the visualization of the existence of the Ca^2+^ ions adsorption sites on TiB_4_ MBene from S1 to S8 are presented in Fig. 14k. The ion diffusion and rapid migration rate between respective electrodes are directly proportional to the diffusion energy barriers because the lower energy barriers of the MBnee electrode possess better rate performance than the electrode material with higher energy barriers. The ion diffusion barriers over TiB_4_ MBene for Li, Na, K, and Ca are 0.29, 0.14, 0.14, and 0.38 eV, the lowest energy barriers the Li ions possess over MBene. The detailed mechanism of the synthesized MBene diffusion energy barriers is presented in Fig. 14m, o. The diffusion visualization for TiB_4_ was first calculated by considering the two paths, as illustrated in Fig. 14l, n. The ion diffusion barriers over TiB_4_ MBene for Li, Na, K, and Ca are 0 0.26, 0.16, 0.24, and 0.67 eV, the lowest energy barriers the Li ions possess over MBene. The detailed mechanism of the synthesized MBene diffusion energy barriers is presented in Fig. 14q, s. The diffusion visualization for TiB_4_ was first calculated by considering the two paths, as illustrated in Fig. 14p, r.

### MBene in Supercapacitors

Supercapacitors (SCs) are a promising type of energy storage device, which possess a significantly higher charge density, quick charge storage performance, higher power production, and long cycling life compared to traditional charge storage devices, especially batteries and fuel cells (FCs) [134, 135]. SCs can be categorized based on the charge storage mechanism into electric double-layered capacitors, asymmetric supercapacitors (ASCs), and pseudocapacitors (PCs) during electrochemical processes [136]. The carbon-based electrode material for EDLC possesses promising stability and poor specific capacitance [137, 138]. The ASCs and PC electrode materials overcome these limitations because of the use of the oxides as electrode materials. Still, these electrode materials also suffer from quick capacity fading with repeated charge/discharge cycles [139, 140]. MBene is a promising electrode material for SCs to overcome these challenges. Wei et al. [141] utilized fluorine-free multiple concentrations of NaOH and etching time to eliminate the Al layer from MoAlB. The concentration and etching time vary from 12, 24, and 48 as NaoOH concentrations vary in order of the one wt% NaOH at room temperature (RT), with different amounts of the Al in the final product. Their investigation shows that removing the Al percentage from MBene is directly proportional to the charge storage capacity of the MBene, where the CV cure occupying the area at a constant scan rate rises as the Al content elimination percentage rises. In other words, the charge storage capacity of the MBene increases as Al removal content increases. The higher charge storage capacity of the MBene is devoted to the significant SSA, higher layered distance, and development of the extra active sites on their interface after the etching process. All these parameters provided additional access to the electrolyte to interact with the MBene interface and store more charge. The existence of the functional groups on the interface of the MBene is responsible for the additional enhancement of the material storage capacity. During the charge storage process, they also verified the impact of the surface functionalities on the interface of the MBene. Their findings show that the MBene surface possesses higher charge storage capacity when its interface contains -O functional groups than the -F functional groups. The synthesized 1/24-MoAl_1-*x*_B cathode for SCs possessed superb conductivity and area capacitance up to 2006 mF cm^−2^ with a retention capacity of up to 80.2% after 5,000 working cycles. The synthesized sample via NaOH etching agent possessed magical energy storage capacity compared to the sample synthesized by LiF/HCl etching. Simply put, the MBene’s ability to store charges directly relates to how much removal is present in the Al layer. It will be increased by decreasing the Al layer concentration in the M–B phase. According to these results, 2D MBenes offer excellent electrochemical resistivity with high energy and power density as SCs. We have tabulated a comparative charge storage capacity, characterization, SSA, d-spacing, and electrochemical performance of MBene in different energy storage fields, which are displayed in Table 3.

**Table 3:** Comparative charge storage capacity, characterization, SSA, d-spacing, and electrochemical performance of MBene and MXene

MBene-based Cathode material	Preparation Route	Type of Storage Device	Electrolyte Used	SSA m^2^ g^−1^	d-spacing (nm)	Electrochemical Performance								Remarks	References
						Capacity	Current Density	Voltage Range (V)	Long-Term Electrochemical Cycling Performance						
									Cs	CurrentDensity	CE	No. of Cycles	Ret %		
MoB	Hydrothermal process	LIBs	1 M LiPF_6_	28.1	0.24	641.7 mAh g^−1^	2 A g^−1^	0–3.00	144.2 mAh g^−1^	2 A g^−1^	98	1000	62.1	The synthesized electrode material has excellent structural stability and electrochemical performance. Its outstanding stability and performance are attributed to MBene’s fluorine-free synthesizing route.	[111]
Ti_3_C_2_T_*x*_ nanosheets	HF Etching	LIBs	1 M LiPF_6_	24	0.27	570 mAh g^−1^	0.1 A g^−1^	0–3.00	570 mAh g^−1^	0.1 A g^−1^	97	80	0.78	The interlayer spacing of the MXene enhancement is responsible for the enhancement of the specific capacity of the material.	[142]
h-MBenes	Acid Etching	LIBs	1 M LiPF_6_	11.4	3.3 to 6.8 Å	420 mAh g^−1^	0.05 A g^−1^	0–3.00	250 mAh g^−1^	0.1	95	600	55.65	They analyzed the characteristics and performance of h-MBene and orth-MBene. h-MBenes have more excellent stability than orth-MBenes due to their increased symmetry, better consistent and effective bonding, advantageous electronic structure, and reduced lattice energy. Due to these considerations, the hexagonal phase exhibits lower energy and better thermodynamic stability than the orthorhombic phase.	[15]
Ti_3_C_2_ MXene	LiF Etching	Li-SBs	1 M LiTFSI	21.9	0.237	844.7 mAh g^−1^	0.2 C	1.6–2.8	7.5 mAh cm^−2^	0.2C	89	100	82.19	The construction of TCM-decorated CNFs by a versatile electrospinning technique serves as a dual-functional interlayer for energizing sulfur and maintaining lithium in improved Li–S batteries. Capitalizing on abundant lithophilic sites and the seamless fibrous network architecture.	[143]
S-MBene/CNT	HCL/LiF Etching	Li-SBs	0.5 M Li_2_S_8_	131.8	0.42	12.73 mAh cm^−2^	21.80 mA cm^−2^	1.8–2.8	11.07 mAh cm^−2^	21.80 mA cm^−2^	87	120	86.96	The enhancement of the electrochemical performance of the MBene by the introduction of the CNTs is devoted to the higher SSA and porosity of the MBene because of their tubular structure. The higher SSA and porous nature of the material provided the extra space for the electrolyte to interact with the electrolyte. The individual layers’ restacking of MBene is the primary disadvantage of MBene. This issue also solved the addition of the CNTs.	[91]
MoAl_1-x_B	Simple Stirring	ASSS	1 M Na_2_SO_4_ 1 M H_2_SO_4_ 2 M NaOH	–	–	2006.60 mF cm^−2^	0.5 mA cm^−2^	0–1.0	–	–	–	5000	80.2	Their findings investigated the impact of the Al removal content on the electrochemical performance of the synthesized MBene because the enhancement of the removal of Al layers from MoAlB exposed higher open spaces on the developed MBene to enhance their specific capacitance.	[141]
MoB MBene	Facile solid-state synthesis	ASCS	H_2_SO_4_	135.10	0.28–0.31 nm	445 F g^−1^	1 A g^−1^	0–2	–	–	97	2000	78	They investigated the impact of the electrolyte nature on the charge storage capacity of the pristine MBene. Their finding showed that the MBene-based material possesses an excellent charge storage capacity in H_2_SO_4_ compared to Na_2_SO_4_ electrolytes. This behavior could be attributed to the size of the ions in the electrolyte and their mobility.	[144]
MoB Mbene	Facile solid-state synthesis	ASCs	Na_2_SO_4_	135.10	0.28–0.31 nm	130 F g^−1^	1 A g^−1^	0–2	–	–	99	2000	88		
Ti_3_C_2_ MXene	Ex situ HF etching	ASCs	KOH	–	0.245 nm	383.3 F g^−1^	1 A/g	− 0.2–0.4	180	1 A g^−1^	–	10,000	94	This study offers significant insights into the design of improved electrode materials for energy storage systems, presenting a promising approach to enhance the performance and durability of ASC devices.	[145]
MBene–MoB	Alkaline etching process	ASSS	Na_2_SO_4_	–	0.617–0.707 nm	4025.60 mF cm^−2^	5 mA cm^−2^	0–1	–	21.80 mA cm^−2^	–	6000	90	The results of their research indicate that the capacitance of MBene–MoB supercapacitors is superior to that of standard 2D materials and that this improvement occurs as the amount of Al in the material steadily decreases. This accomplishment can be due to a synergistic interaction between surface-controlled and diffusion-controlled capacitance storage processes and fast electrolyte diffusion within the interlamellar space.	[146]
Ti_3_C_2_T_x_	LiF/HCl etching	ASSS	1 M H_2_SO_4_	93.088	–	428 F cm^−2^	10 mA cm^−2^	− 0.3–0.5	–	10 mA cm^−2^	98	8000	94.9	The incorporation of graphene and carbon nanotubes into novel MXene materials presents significant potential for the scalable production of flexible supercapacitors for wearable electronic applications.	[133]
CoO/MoB	Exfoliation	LIBs	1 M LiPF_6_	18.8525	0.245–0.413 nm	999.4 mAh g^−1^	100 mA g^−1^	0.01–3.0 V	601.3 mAh g^−1^	1000 mA g^−1^	73.3	600	85	The combination of the MBene with CoO improves their electrochemical performance, structural stability, prevents their volume expansion, lowers the barrier, and boosts electron transfer.	[147]
CrB_4_	Theoretical calculation	CaIBs	–	–	–	3377 mA h g^−1^	–	–	–	–	–	–	–	As the low activation barrier values showed, Ca^2+^ ions moved more quickly through the MB_4_ monolayer. In addition, the metallic structure of the materials was preserved entirely even after the materials absorbed the whole quantity of Ca ions. These features indicate that monolayers predicted MBenes are viable materials for anodes for CIBs, with noteworthy rate capacities because of their high-rate capacities.	[63]
MoB_4_	Theoretical calculation	CaIBs	–	–	–	2311 mA h g^−1^	–	–	–	–	–	–	–		
WB_4_	Theoretical calculation	CaIBs	–	–	–	1416 mA h g^−1^	–	–	–	–	–	–	–		
Ti_3_C_2_ MXene	Theoretical calculation	CaIBs	–	–	–	319.8 mA h g^−1^	–	–	–	–	–	–	–	MXenes possess strong conductivity, an architecture of layers, and diverse surface chemistry, facilitating effective calcium-ion storage, rapid ion diffusion, and superior cycling stability, rendering them promising candidates for CaIBs.	[148]
TiB_4_	Theoretical calculation	CaIBs	–	–	–	1176 mA h g^−1^	–						–	The higher Ca^2+^ charge storage capacity of TiB_4_ than SrB_8_ MBene is because of the promising electronic structure, extremely low diffusion barrier, and open-circuit voltage.	[133]
SrB_8_	Theoretical calculation	CaIBs	–	–	–	616 mAh g^−1^	–						–		

## MBene in Energy Harvesting

Energy harvesting (EH) is the cost-effective, sustainable, and promising technology of the twenty-first century. It is the process in which mechanical load, temperature gradient, vibrations, and light are used to create power or electrical energy, and it is called energy harvesting. The performance of the HE system depends on the amount of energy, energy conversion to electrical energy, and size limitations. The advanced EH techniques are mechanical, thermal, magnetic, electronic, and biochemical. The concept of the advanced EH originated in the 2000s. In the advanced EH concept, non-renewable energy sources, including heat, light, and vibration, are utilized as feedstock to save the environment and reduce their concentration on the environment. While some approaches and strategies are being developed, their full potential remains unknown. The EH will support green innovations that save energy and lower CO_2_ emissions, making them essential for building sustainable societies and smart cities of the future.

### CO_2_ Reduction Reaction

The termination of the interface of MBene has a viable impact on the CO_2_ catalytic performance of the MBene and its adsorption. The pristine MBene generally has poor catalytic performance compared to the functionalized MBene. The investigation of the MBene practical surface functionality for the adsorption of CO_2_ is still under investigation. However, some published theoretical research studies may show that O-terminated MBene possesses excellent CO_2_ catalytic and adsorption performance [13, 150, 151]. Because of the -O-terminated functional groups on the surface of the MBene, highly active and reactive sites have been developed on the MBene surface to capture CO_2_ [152, 153]. Compared to the other surface functionalities, MBenes rich with O-terminated functionalities interact strongly with CO_2_ species via van der Waals interactions. Similarly, The –OH and –O-based MXene have excellent CO_2_ capture efficiency. Through the formation of hydrogen bonds and the exchange of electrostatic forces, these groups interact with CO_2_ molecules. Because the –OH and –O surface functionalities on MXenes are responsible for forming hydrogen bonds with CO_2_, the molecules of CO_2_ are effectively anchored to the outermost layer of the MXene [153, 154]. The effective charges on the interface of the MBene and CO_2_ molecules facilitated the strong adsorption by the electrostatic attraction between them. The MBene material rich in O termination possesses excellent CO_2_ adsorption compared to the -F and -O terminations.

Similarly, MXene-based catalyst rich in O termination possesses an excellent CO_2_ adsorption capacity of up to 19.8 wt% at 298 k and 1 atm [31]. These findings show that the MBene surfaces rich in O termination possess an outstanding CO_2_ reaction activity compared to the pristine MBenes and MXenes. The valuable significance that O surface functional groups play in carbon dioxide absorption is brought to light by increased catalyst adsorption capability. Similarly, the O-surface-rich MBene possesses a promising selectivity to separate the CO_2_ from N_2_. MBene-based membranes terminated with oxygen could maintain stability in humid circumstances, a vital quality for practical use [13]. Regarding CO_2_ capture uses, oxygen-terminated MBenes are the most attractive ingredients. They are attractive to carbon dioxide molecules, are not overly challenging to produce, and can maintain stability in humid environments. These MBenes, once they have been created, exhibit good stability, meaning they can keep their structural integrity and functional capabilities even when exposed to moisture. This is an essential benefit for use in the real world. Still, additional research is required to enhance these materials’ optimization further and advance the development of carbon capture.

**Fig. 15:**
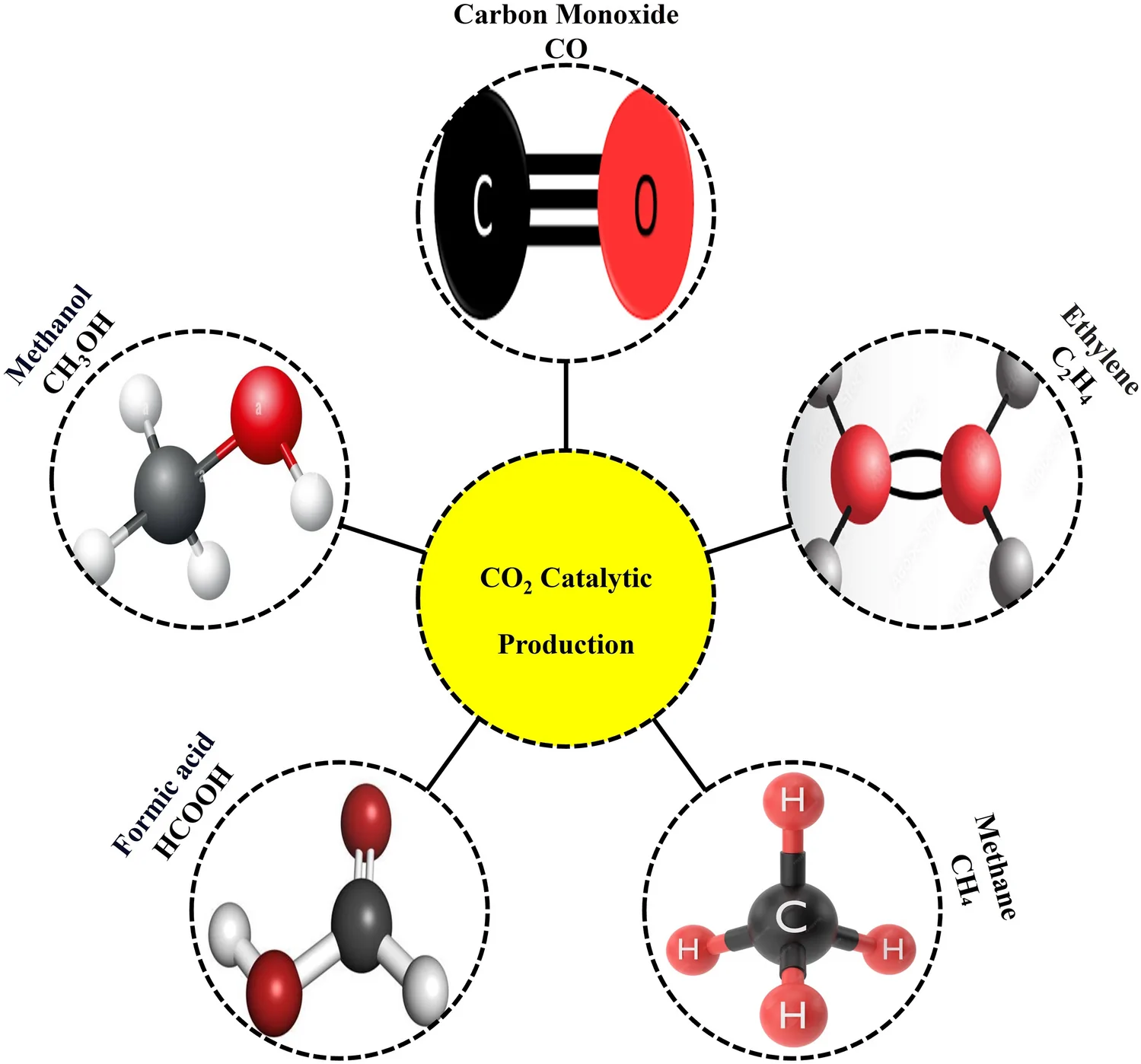
Production of the valuable products by the catalytic conversion of the CO_2_ (adapted with permission [149])

The CO_2_ reduction reaction (CO_2_RR) is the electrochemical process in which CO_2_ is converted into valuable products, as shown in Fig. 15. It is of considerable significance that this technique contributes to a circular carbon economy by reducing carbon emissions and producing products with value-added from carbon dioxide [155]. In the electrochemical cell, the CO₂RR was taking place at the cathode. The production rate of the final product during the CO₂RR in an electrochemical cell depends on the nature of the cathode material, applied potential, reaction conditions, and nature of the electrolyte. CO_2_ adsorption and activation are the preliminary and essential steps for successful and effective CO_2_RR [156]. The production reaction of the various products from CO are presented below.

Synthesizing of the CO and HCOOH by two-electron reduction:

1 $${\mathrm{CO}}_{2} { } + 2{\mathrm{H}}^{ + } { } + 2{\mathrm{e}}^{ - } { } \to {\text{CO }} + {\text{ H}}_{2} {\mathrm{O}}$$

2 $${\mathrm{CO}}_{2} + 2{\mathrm{H}}^{ + } { } + { }2{\mathrm{e}}^{ - } { } \to {\mathrm{HCOOH}}$$

Synthesizing of the formaldehyde by four-electron reduction:

3 $${\mathrm{CO}}_{2} { } + 4{\mathrm{H}}^{ + } { } + 4{\mathrm{e}}^{ - } { } \to {\text{ H}}_{2} {\mathrm{C}} = {\text{O }} + {\text{ H}}_{2} {\text{O }}$$

Synthesizing of methanol by six-electron reduction:

4 $${\mathrm{CO}}_{2} { } + 6{\mathrm{H}}^{ + } { } + 6{\mathrm{e}}^{ - } { } \to {\text{ CH}}_{3} {\mathrm{OH}} + {\text{ H}}_{2} {\mathrm{O}}$$

Synthesizing of Methane by eight electron reduction:

5 $${\mathrm{CO}}_{2} { } + 8{\mathrm{H}}^{ + } { } + 8{\mathrm{e}}^{ - } \to {\text{ CH}}_{4} + 2{\mathrm{H}}_{2} {\text{O }}$$

**Fig. 16:**
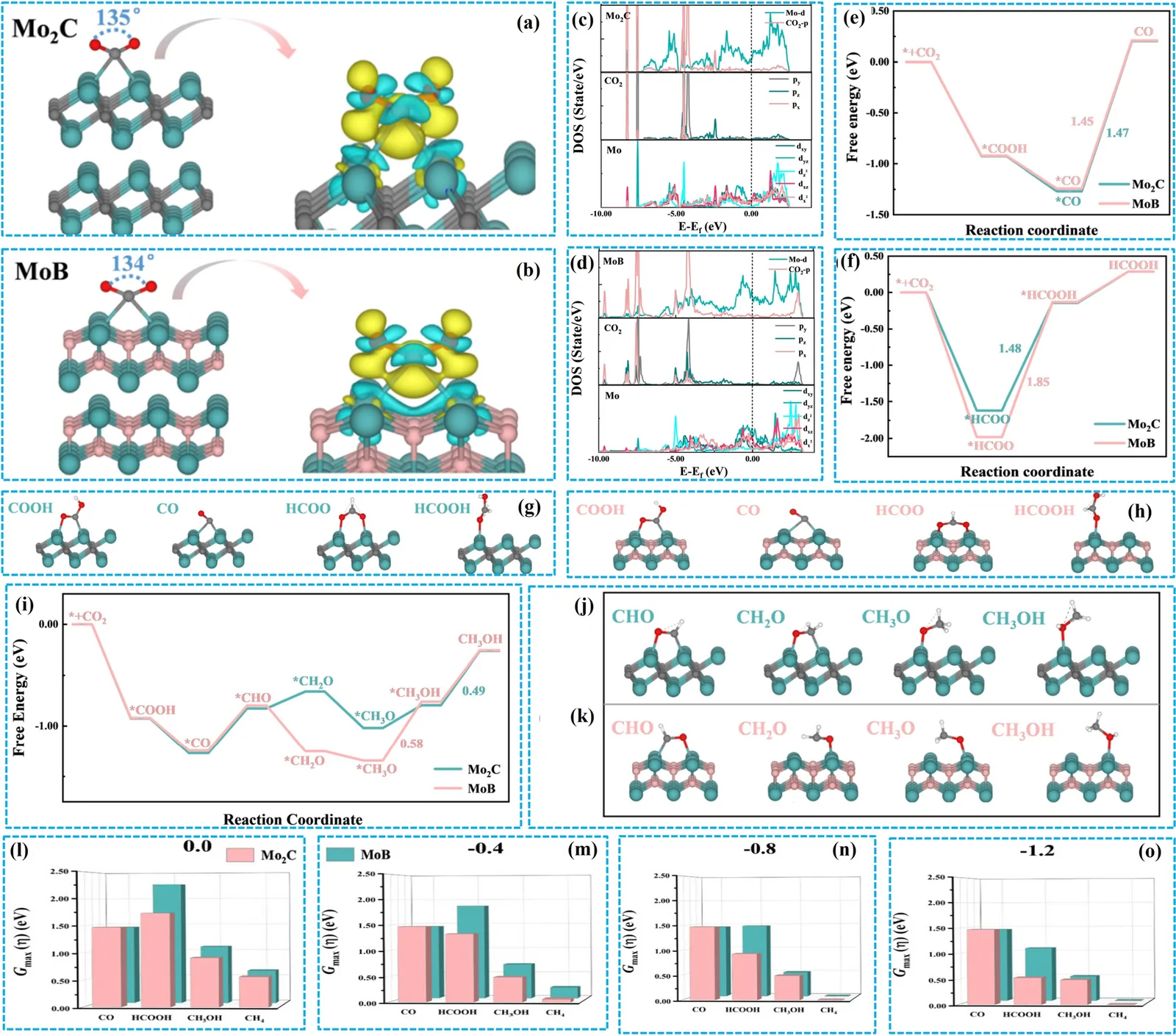
Stable CO_2_ adsorption sites and charge density difference on **a** MoC MXene and **b** MoB MBene (Yellow and cyan areas signify electron accumulation and depletion). DOS of CO_2_ adsorption on **c** Mo_2_C MXene and **d** MoB Mbene. Free energy diagram of **e** Co and **f** HCOOH on a MoC MXene and MoB Mbene. The improved intermediate adsorption formations on **g** Mo_2_C and **h** MoB. **i** Free energy illustrations of the CO_2_RR to CH_3_OH. The optimized intermediate adsorption configurations on **j** Mo_2_C MXene and **k** MoB Mbene. **l-o** The maximum free energies of HER and CO_2_RR to CO, CH_3_OH, CH_4_ on Mo_2_C and MoB at four applied potentials (U = 0.0, 0.4, 0.8, and 1.2 V versus RHE) (adapted with permission [158])

In this regard, Liu et al. [92] utilized the four types of MBene, including Cr_2_B_2_, Mn_2_B_2_, Fe_2_B_2_, and Mo_2_B_2_, as catalysts to convert the CO_2_ into methane (CH_4_). All four MBene consist of four layers: two middle layers of boron and two upper and lower metallic layers. Their comparative analysis shows that MBene possesses a more magical CO_2_ to CH_4_ conversion than a conventional Cu^3+^ catalyst. This suggests that MBenes are superior to traditional metallic catalysts. The outcomes demonstrate that MBenes’ distinct electrocatalytic efficiency is caused by the boron layer being adjusted to the charged atmosphere on the metal surface. Nevertheless, it is essential to remember that a solid adsorption contact may hurt CO_2_’s hydrogenation to COOH. Gibbs free energy is significantly increased during the hydrogenation of CO to CHO in the following hydrogenation step from COOH to CH_4_. However, other MBenes Mo_2_B_2_ comparatively possess smaller Gibbs free energy in forming COOH to CH_4_. Researchers also assessed the potential for the production of methanol on Fe_2_B_2_. The selective capacity of CO_2_RR is enhanced by the weak HER catalytic efficiency of Cr_2_B_2_ and Mo_2_B_2_ in the selectivity benchmark. On the other hand, MBenes’ performance in the ultimate production and desorption of H_2_O was superb. Xiao et al. [157] inspected the potential of MBene in energy harvesting by quantum mechanical screening of 2D MBene. The catalytic performance of the MBene in the HER and CO_2_RR reaction was evaluated based on the various limiting potentials U_L_ (CO_2_RR). This makes it easier for CO_2_ to convert to *HCO_2_ or *COOH and promotes higher-quality end-product manufacturing of products on the MBenes electrode. Researchers examined that MBenes were robust and exhibited an average binding of CO_2_RR intermediates with complete optimization of these structures. The binding power of the intermediates on these MBene catalysts dictated every feasible pathway for the chemical process. Since the Gibbs energy profile diagram was used to design the most favorable reaction route, all reaction steps had to be made downward in the free energy profile to establish the applied potential based on the minimum limiting potential. The lower potential of the CO_2_ conversion into CO makes MBene an ideal candidate for energy harvesting. Lu et al. [158] employed the first-principles analysis to compare the electrochemical CO_2_RR performance of the MBene and MXene-based catalysts. They compared the theoretical MoB MBene-based catalyst catalytic performance with conventional Mo_2_C MXene. The theoretical calculation of the MBene possesses an excellent metallic nature and electrical conductivity. The MBene-based catalyst possesses a promising interaction energy of up to 3.64 eV with CO_2_ compared to the Mo_2_C MXene. The DOS and charge difference density confirm the valuable charge transfer from MoB MBene to CO_2_. The inhibited hydrogen evolution process and low reaction energy for the CO_2_RR are two factors that contribute to MoB’s better catalytic efficiency. The most CO_2_-suitable adsorption sites on the Mo_2_C MXene and MoB MBene interface are presented in Fig. 16a, b. Similarly, the DOS of CO_2_ adsorption on Mo_2_C MXene and MoB MBene are presented in Fig. 16c, d. The chemical adsorption of CO_2_ on the interface of the MoB MBene at the C bonding with two respective Mo atoms and the angle between O–C–O is bent to 134 degrees. According to the calculations, the interaction energy of O_2_ deposited on MoB is − 3.64 eV. Because electrons are transferred from Mo and B atoms to the CO_2_ molecule, the CO_2_ molecule is successfully activated for the subsequent reactions. The chemical adsorption of CO_2_ on the interface of the Mo_2_C MXene at the C bonding with two respective Mo atoms and the angle between O–C–O is bent to 180^o^ to 135°.

These findings show that the MBene CO_2_ adsorption energy is slightly higher than the Mo_2_C MXene. MoB exhibits a process for CO_2_ adsorption and activation comparable to Mo_2_C. Furthermore, the Mo 3*d* orbitals in MoB, located at 0.60 eV, accelerate the chemical reaction with CO_2_. The production of CO, HCOOH, CH_3_OH, and CH_4_ through CO_2_RR reaction are given in Eqs. 1–5. The free energy requirement for the catalytic conversion of the CO_2_ into the targeted product on the catalytic surface of the Mo_2_C MXene and MoB MBene is presented in Fig. 16e, f. The finding shows that the MBene-based catalyst possesses a slightly better catalytic performance than the conventional MBene catalyst. The CO_2_ reaction with HCOOH is unfavorable on Mo_2_C MXene and MoB MBene because the *HCOO intermediate is excessively adsorbed, leading to instability. The optimized intermediate adsorption configurations on Mo_2_C MXene and MoB MBene are presented in Fig. 16g, h. They also investigated the free energy required for forming the CH_3_OH by CO_2_RR reaction on the MoC MXene and MoB MBene, as shown in Fig. 16i-k. The findings indicate that both MoB and Mo_2_C exhibit comparable capacity for CH_3_OH production, and the conversion of the CO_2_ to CH_3_OH is followed by Eq. 3. Additionally, it has been discovered that the active sites of Mo have a more significant attraction to the O-end adsorbed intermediates, such as *CH_2_O, *CH_3_O, and *CH_3_OH. Additionally, the presence of boron in MoB can further enhance this attraction. The electrochemical process’s CO_2_RR activity and product selectivity are subject to change depending on the applied voltage and can fluctuate [159]. The impact of the potential alteration on the targeted species’ production rate is presented in Fig. 16l-o. Based on these findings, MoB is an adequate substitute for Mo_2_C MXene in the CO_2_RR to CH_4_ conversion process. Considering the remarkable performance of MXenes, we anticipate that MBene materials will have a wide range of potential applications in electrocatalysis.

### Catalytic Mechanism of the MBene-based Catalyst for NRR Application

**Fig. 17:**
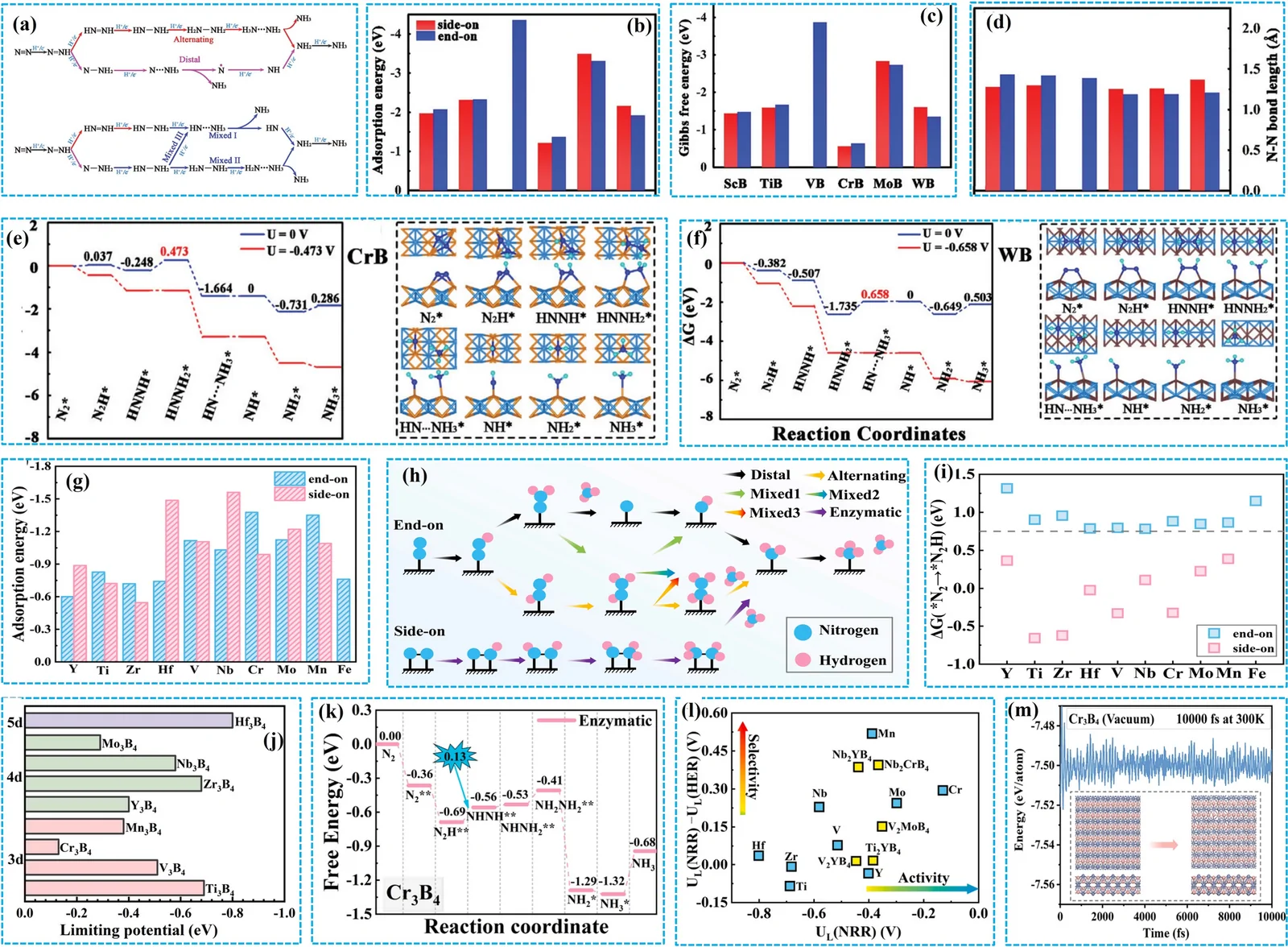
**a** Mechanism of the reduction of N_2_ to NH_3_. **b** N_2_ on MBene adsorption energy. **c** Gibbs free energy of N_2_ molecules. **d** N–N bond length on the surface of the MB monolayer. **e–f** Gibbs free energy illustrations and the consistent structures of intermediates of N_2_ conversion into NH_3_ on CrB, and WB monolayers (adapted with permission [170]). **g** Adsorption energy of the various MBene at end-on and side-on locations. **h** Selective paths for the reduction of the N_2_ to NH_3._ **i** Gibbs free energy value on the side-on and the end-on. **j** U_L_ of nine Mbene. The enzymatic mechanism served as the basis for the calculation of free energy diagrams for NRR **k** Cr_3_B_4_ Mbene. **l** Plot of U_L_(NRR)–U_L_(HER) as a function of U_L_(NRR) on catalysts, **m**) stability of MBene-based catalyst (adapted with permission [171])

The NH_3_ is considered a primary chemical feedstock for producing medicines, chemicals, and fertilizers, which possess high hydrogen content and gravimetric energy of ∼17.7 wt% and 3 kWh kg^−1^ [160]. Transportation of the NH_3_ from one place to another is more convenient than transportation of the H_2_ in ambient conditions. The promising supremacy of NH_3_ over traditional sources is that it has been investigated as a potential zero-carbon energy source for various uses, such as fuels, including direct NH_3_ fuel cells and the generation of H_2_ (by NH_3_ cracking). In 1931, Carl Bosch was awarded the Nobel Prize in Chemistry for transforming the intensive Haber–Bosch (H–B) process into commercial-scale production of NH_3_. The H–B process is enabled by high pressures and temperatures of 100 − 150 bar and 350 − 450 °C with the uses of gray/blue H_2_, which annually consumes 1 − 2% of global energy (22.2 GJ $\mathrm{t}_{\mathrm{NH}_3}$^−1^) and emits ∼1.3% of global CO_2_ (1.2 $\mathrm{t}_{\mathrm{CO}_2}$^−1^ $\mathrm{t}_{\mathrm{NH}_3}$^−1^) [161, 162]. The promising alternative pathways for NH_3_ synthesizing are renewable energy sources that play a key role in mitigating environmental and climate effects. The catalytic conversion of N_2_ and nitrogen oxides (NO_*x*_) into NH_3_ is a cutting-edge, safe, and economically feasible technique to minimize their environmental concentration. The design and synthesis of the SACs is an emerging technique for developing cost-effective and high-efficiency photocatalysts. Despite the various benefits of SACs, there are a few main drawbacks regarding nitrogen reduction reaction (NRR), most notably the low stability of single-atom photocatalysts [163–165]. It is essential to establish an exemplary configuration, which includes developing a modular strategy to guarantee the formation of transplanted reactive sites and accurately controlling the interactions between the active centers and semi-conductive supports during the synthetic procedure. Based on the drawbacks of traditional SACs and DACs, the scientific community needs to develop a new, advanced, and promising catalyst with improved catalytic performance and stability to convert N_2_ to NH_3_. So, in this section, we discussed the catalytic performance of the newly developed material MBene as a catalyst to convert N_2_ to NH_3_. The excellent choice of catalyst for NRR possesses low electrocatalytic denitrification to minimize the HER reaction on the catalyst’s surface [166]. It also possesses extremely low overpotential to break the triple bond in N_2_ molecules to facilitate ammonia’s rapid development [167, 168]. The adsorption and activation of N_2_ are the primary parameters for selecting the catalyst, which is the electrochemical N_2_ reduction into NH_3_ [169]. Li et al. [170] employed theoretical calculations to investigate nitrogen adsorption on the interface of the six ScB, TiB, VB, CrB, MoB, and WB and their electrocatalytic NRR process for the MBene-based electrode materials. Their findings show that the MBene-based electrode material possesses an excellent N_2_ adsorption capacity and nitrogen reduction to ammonia. The MBene-based electrocatalyst exhibits three general pathways in the NRR process for synthesizing ammonia from hydrogen and dinitrogen, as illustrated in Fig. 17a. All types of MBene are predicted to have different N2 adsorption properties at their interface. Generally, the N_2_ adsorption energy on the interface of the MBene varies from − 1.215 to − 4.357 eV, as shown in Fig. 17b. This suggests that these MB monolayers possess enough capacity to retrieve and activate N_2_ molecules. Similarly, all MBene possess a Gibbs free energy of N_2_ molecules on the interface of the theoretical predicted MBene in the range of − 0.566 to − 3.871 eV, as presented in Fig. 17c. The results of this study suggest that these MB monolayers can spontaneously catch nitrogen dioxide molecules. When compared to the other six MB single layers, the WB, a single layer with the end-on orientation, is the one that is most likely to adsorb N_2_ molecules. On the other hand, the binding of N_2_ molecules on the CrB in a single layer is far less effective than on other MB monolayers. Generally speaking, the N_2_ molecules that are spontaneously collected are advantageous for inducing the NRR phase. The adsorption of the N_2_ on the MBene interface effectively improves the bond length. It weakens the bond between N_2_ molecules, as present in Fig. 17d. The bond energy of the N_2_ molecules increases from 1.114 to 1.434 Å after the adsorption of the MBene. Compared to the other five kinds of MBene, VB MBene possesses excellent adsorption of N_2_ molecules. Similarly, in all MBenes, CrB monolayer MBene possesses the weakest adsorption of the N_2_ on their interface of the MBene. In a nutshell, all MBene possess an acceptable N_2_ adsorption and an excellent NRR reaction potential. The five selective paths proposed for the catalytic performance of the MBene for the NRR reaction process. These paths are alternating, distal, mixed I, II, and III. All MBene possesses an exact mechanism of six-electron reaction such as N_2_ + 6H^+^  + 6e^−^  → 2NH_3_. The ΔG for the possible pathway and the corresponding structure of WB and CrB are presented in Fig. 17e, f. These research findings have determined that VB, CrB, and MoB monolayers exhibit remarkable catalytic activity for the NRR, making them potentially attractive candidates for the role of NRR electrocatalysts. The technique presented here offers a practical alternative to catalysts made of noble metals and a theoretical foundation for developing highly active electrocatalysts made of MBene. Zhang et al. [171] employed the first principal calculation to investigate the N_2_ bond breaking and conversion into NH_3_ of ten M_3_B_4_-type MBenes. Among all ten predicted MBne types, M_3_B_4_ MBenes possess excellent NRR electrocatalytic activity. Generally, the adsorption energy of the N_2_ over MBene-based catalyst is found at two different adsorption sites, end-on, and side-on, because adsorption arrangements can significantly affect NRR routes. The details of the adsorption energy of the various MBene at end-on and side-on locations are presented in Fig. 17g. All the MBene possess an end-on and side-on arrangement with negative adsorption values in the range of the − 0.60 and − 1.56 eV. The bond length of the N_2_ before adsorption is 1.11 Å, which can be enhanced at end-on and side-on up to from 1.13 to 1.14 and 1.16 ~ 1.22 Å, respectively, after adsorption on MBene. This suggests that the adsorbed N_2_ molecules are efficiently activated. These charge density difference graphs demonstrate a significant buildup of electrons between the N_2_ and TM atoms in MBenes, which indicates the establishment of strong chemical bonds. In addition, there is an apparent lack of electrons between the two N atoms, which reveals a weaker N_2_ bond that is more likely to be targeted by protons that are larger in number. Their findings show that the electron migration from the MBene interface to adsorbed N_2_ molecules at side-on and end-on are 0.55 ~ 0.94 e and 0.26 ~ 0.42 e. These data pieces, including an increasingly substantial charge transfer between N_2_ and catalysts and the longer N_2_ bond of adsorbed N_2_, demonstrate that the activation of N_2_ molecules becomes much more complete when the adsorption process takes place in a side-on configuration environment. The selective paths for the reduction of the N_2_ to NH_3_ are presented in Fig. 17h. The value of Gibbs free energy at the side-on and end-on is − 0.66 to 0.39 eV and 0.78 to 1.31 eV. So, the Gibbs free energy value on the side-on is greater than the end-on, as presented in Fig. 17i, j. The results of this study suggest that there is a thermodynamic propensity for H atoms to be boned to the adsorbed N_2_** through the use of enzymatic pathways. This inference corresponds with the higher charge transmission and more extended N_2_ bond elongation indicated earlier about the side-on arrangement.

Consequently, the end-on arrangement is ignored since it cannot catalyze the conversion of N_2_** into N_2_H** in an effective manner. The U_L_ of nine MBene are predicted in Fig. 17k. The U_L_ values of the nine MBene and Ru are − 0.13 ~ -0.80 and − 0.98 V; these findings show that the U_L_ values of the MBene are lower than the Ru catalyst. Additionally, the catalytic performance of the MBene is presented in Fig. 17l. These MBenes have good selectivity and may successfully suppress rival HER. Lastly, our AIMD simulations demonstrate that, except for Mo_3_B_4_, all MBenes exhibit exceptional stability in ambient environments (Fig. 17m**)**. These intriguing findings offer insight into using M3-nM’nB_4_ (*n* = 0, 1) MBene as newly developed catalysts for NRR and provide a logical catalyst design approach.

Table 4 delineates several MBene materials investigated for NRR and CO₂RR applications, emphasizing their target products, reaction types, and optimal operating potentials. The electrocatalytic efficacy of MBenes in the nitrogen reduction reaction (NRR) and carbon dioxide reduction reaction (CO₂RR) demonstrates significant potential relative to their MXene equivalents. In NRR, MBenes like CrB, Cr₃B₄, and vacancy@Mo₂B₂ exhibit low overpotentials (− 0.13 to − 0.81 V) and efficient NH₃ synthesis, with vacancy engineering (e.g., in Mo₂B₂) further augmenting catalytic performance by generating many active sites. Conversely, the majority of MXenes (e.g., Ti_3_C_2_T_*x*_, V_2_CT_*x*_) often have elevated overpotentials (> − 0.6 V) and face challenges with inadequate selectivity owing to intense competition from the hydrogen evolution process (HER). Furthermore, the B sites in MBenes often provide modest binding energies for N₂ activation, enhancing the selectivity of the nitrogen reduction reaction compared to other MXenes.In CO₂RR, MBenes such as Au_2_B and MoB generate CH₄ at comparatively advantageous potentials (0.46 and − 0.62 V), exhibiting notable product selectivity. In contrast, MXenes often yield CO or formate with moderate Faradaic efficiency and little control over C–C coupling products. Their surface terminations (e.g., –OH, –O, –F) may restrict CO₂ adsorption and the stability of intermediates. Consequently, MBenes, due to their distinctive electronic configurations, stable B-metal frameworks, and adjustable defect chemistry, seem to surpass several traditional MXenes in both nitrogen reduction reaction (NRR) and carbon dioxide reduction reaction (CO₂RR), particularly regarding selectivity and possible operational efficiency.

**Table 4:** Comparative analysis for NRR and CO_2_ RR for MBene and MXene

Name of Material	Synthesizing Routes	Reduction Technique	Name of Conversion Technique	Synthesizing Products	Optimal Potentials	F.E	References
MoB	Hydrothermal technique	NRR	Electrocatalytic	NH_3_	− 0.2 V	55.97	[172]
Cu–Ti_3_C_2_T_*x*_	Sonication	CO_2_RR	Electrocatalytic	CO	− 1.0 V	58.4	[173]
Mo_2_B_2_	DFT	CO_2_RR	Electrochemical	CH_4_	− 0.45V	-	[92]
Ag/Ta_2_CT_*x*_	Hydrothermal technique	CO_2_RR	Electrochemical	CO	− 0.95 V	75	[174]
Au_2_B	DFT	CO_2_RR	Electrocatalytic	CH_4_	0.46 V	–	[157]
Ti_3_C_2_T_*x*_	Simple ttching	CO_2_RR	Electrochemical	Formate	− 2.8 V	90	[175]
MoB	DFT	CO_2_RR	Electrochemical	CH_4_	− 0.62 V		[158]
CrB	DFT	NRR	Electrocatalytic	NH_3_	− 0.277 V	-	[170]
Ti_3_C_2-_MXene	Molten salts etching	NRR	Electrocatalytic	NH_3_	− 0.7 V	92.13	[176]
Cr_3_B_4_	DFT	NRR	Electrocatalytic	NH_3_	− 0.13 V	–	[171]
Pd–Cu/MXene	Magnetic stirring	NRR	Electrochemical	NH_3_	− 0.8 V	45	[177]
ZrB	DFT	NRR	Electrocatalytic	NH_3_	0.65 V	–	[89]
Ti_3_C_2_T_*x*_	Chemical tching	NRR	Electrochemical	NH_3_	− 1.0 V	98.7	[178]
Vacancy@Mo_2_B_2_	DFT	CO_2_RR	Electrocatalytic	CH_4_	− 0.81 V	–	[179]

### Hydrogen Evolution Reaction

The hydrogen evolution reaction (HER) depends on the nature of the electrode material because all of these reactions are carried out at the surface of the electrode. Platinum (Pt) is a widely used electrocatalyst because of its practical and magical electrochemical performance. Unfortunately, its expense and limited availability scale out its industrial use. So, the scientist pays their potential to generate cost-effective and readily available material as the alternative electrocatalyst with more excellent or close electrochemical performance as Pt. Various 2D materials have been investigated and experimentally utilized as HER. In different kinds of 2D material, transitional metal carbides and nitrides caught the attention of scientist because of their silent theoretically and experimentally, their excellent metallic nature, extra active sites, and significant SSA [180–183]. The generation of the mixed various functionalities on the surface of the MXene, such as –F, –Cl, –O, and –OH, is responsible for the uncontrollable and complex atomic composition of the MXene. So, industrialists urgently want a controlled atomic composition-based 2D material with magical catalytic activity. The electrode material’s performance in HER depends on the value of the ΔG in –eV. If the value of the ΔG is less than Pt (0.09 eV) electrocatalysts, it is considered that the synthesized MBene electrocatalyst performs better than Pt and vice versa. So, MBene is a good choice for the HER because MBene gives the best results as electrode material in charge storage devices, as discussed previously.

Additionally, it is always necessary to integrate various reactions into a single device for the energy conversion and storage indicated above. The reusable metal–air battery redox rate highly depends on oxygen-reversible electrochemical reactions occurring at cathodes. When dual-functional or even multifunctional catalysts operate under identical ideal circumstances, they demonstrate significant advantages in facilitating the system’s architecture and lowering costs, compared to single-function catalysts, because two distinct catalysts for various reactions always require special machinery to attain optimal operating conditions. The general condition for the HER active material is that it is overpotential and hydrogen adsorption (ΔG_H_) is less than 0.2 and 0.2 eV, respectively [184, 185].

Additionally, the fact that the catalytic efficiency is frequently more significant than single-function catalysts is what matters most. Applications for heterogeneous catalysts in HER and OER are substantial in advancing electrochemical processes [186–188]. Nevertheless, these catalysts have limited bifunctional activity, high expenses, and shortages, seriously restricting their commercial uses [189, 190]. The intrinsic linear scaling relations (LSR) among reaction intermediate adsorption energies frequently cause OER and OER catalysts to have large intrinsic overpotentials and slow reaction kinetics [191].

**Fig. 18:**
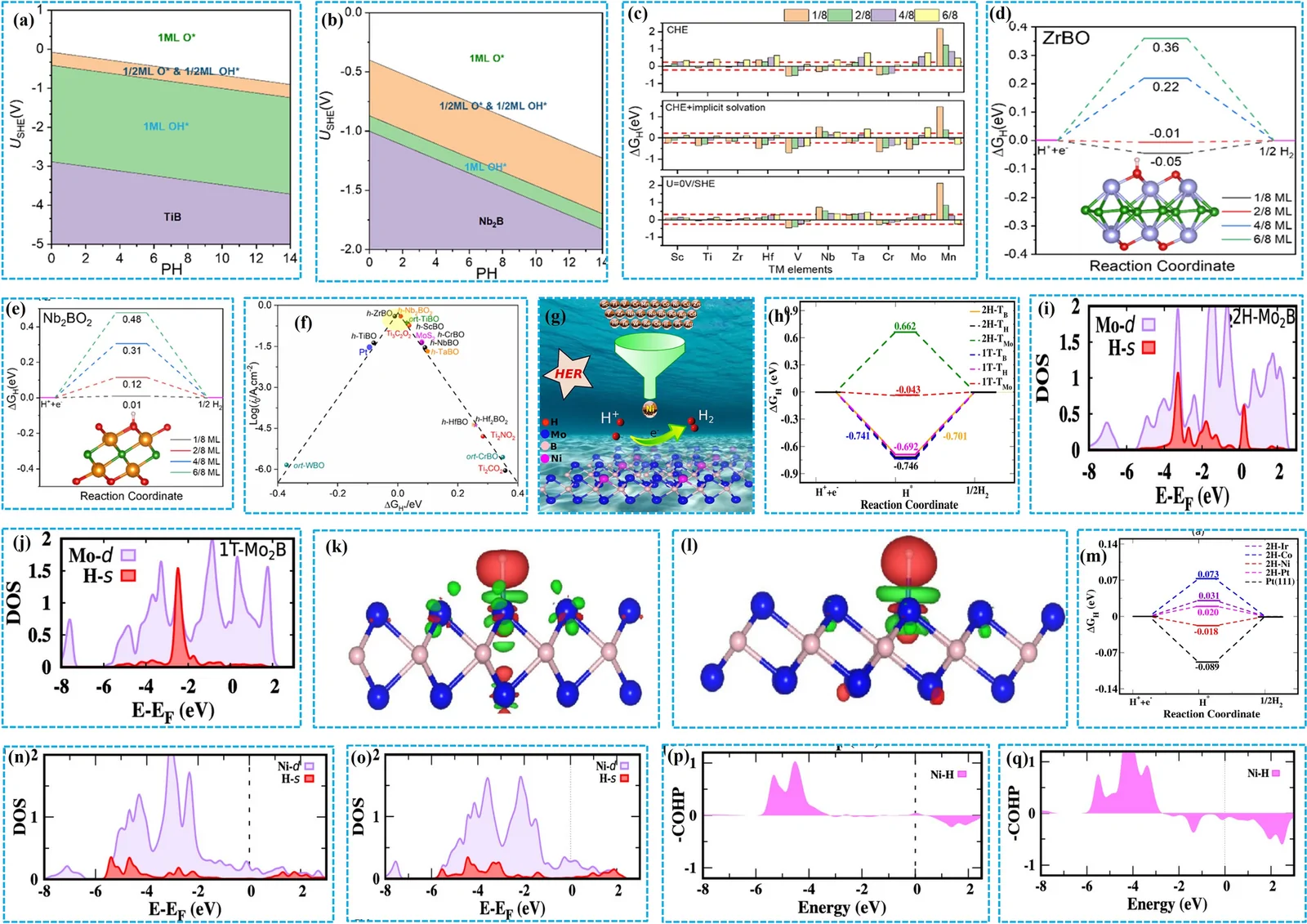
Surface Pourbaix diagrams of **a** h-TiBTx, **b** h-Nb_2_BT_*x*_; **c** Evaluated ΔG_H*_ on different H coverages of 1/8, 2/8, 4/8, and 6/8 ML for h-MBenes MBO consuming the computational hydrogen electrode (CHE), Enlarged results of ΔG_H*_ considered by the CHE model in c) on numerous H coverages for **d** ZrBO and **e** Nb_2_BO_2_; **f** volcano curve of designed exchange current densities log (i0) vs. ΔG_H*_ on h-MBenes, ort-MBenes, and MXenes functionalized with O (adapted with permission [45]). **g** HER mechanism. **h** Obtained ΔG_H_* values of pure Mo_2_B MBene for obtainable sites. PDOS of H adsorption on the TMo site of original, **i** 2H and, **j** 1TMo_2_B. The charge density difference of H adsorbed on the TMo site of original **k** 2H and **l** 1 T Mo_2_B. **m** Intended ΔG_H_* on SACs and Mo_2_B for the 2H phases. PDOSs of Ni-Mo_2_B for the **n** 2H and **o** 1 T phases. COHP between Ni–H bonds of the **p** 2H and **q** 1 T stages (adapted with permission [199])

Moreover, it was scientifically verified that MBene exhibits good potential as a cathode in Li–S and LIBs. The basal plane atoms of pure MBene can exhibit anticipated catalytic active sites for effective electrochemical processes. This contrasts with the newly emerging 2D catalysts, such as MoS_2_, graphene, and g-C_3_N_4_, where the catalytically active sites are restricted to the exposed edges or defective atoms, while the bulk of in-plane particles remain catalytically inert [192–194]. This might reduce the need for stringent condition optimization and other techniques to generate appropriate doping or defect sites and enhance the material’s unique activity. Furthermore, in the case of MXene, MBene can be stabilized efficiently without surface passivation groups, so it successfully guards against poisoning of the surface reaction center. Miao et al. [45] employed the theoretical prediction to investigate the HER performance 15 h-MBene. Their theoretical prediction shows that the four h-MBenes possess excellent antiferromagnetic metal characteristics and acceptable electrocatalysis for HER. The stability of the catalytic material is crucial during the process of the material during the process HER. In this regard, the impact of the functional groups –O and –OH on the h-MBenes’ stability was investigated using Surface Pourbaix diagrams as presented in Fig. 18a, b. Their findings show that the functionalized MBene is stable during the HER process. The impact of the pH on the catalytic activity of the h-MBenes in HER was assessed by conducting the Gibbs free energy (ΔG_H*_) of the MBenes with vary in hydrogen coverage. Their outcomes show that the Gibbs free energy value of the h-MBenes in HER continuously increases by increasing the hydrogen coverages. The detailed mechanism of how the enhancement of the hydrogen content impacts the h-MBenes in HER performance is presented in Fig. 18c-e. The Gibbs free energy value of the eight h-MBenes is lower than the 0.2 eV to prove that MBene possesses excellent HER characteristics. It was discovered that h-M_2_BO_2_ demonstrates a lower HER activity level than h-MBO, and most adsorption Gibbs free energies estimated for h-M_2_BO_2_ display a positive value. A reduced B content is present in h-M_2_BO_2_, which can be attributable to this phenomenon [195]. The volcano curve was plotted to provide a comparative analysis of the catalytic performance of the h-MBenes, ort-MBenes, and MXenes, as presented in Fig. 18f.

The placement of the h-ZrBO and h-TiBO MBene on the left side of the volcano curve shows the powerful interaction between the outer surface of the H and O atoms. However, in contrast to other systems, there is a greater degree of desorption between the surface oxygen and hydrogen. In the volcano curve, the h-MBO and h-Nb_2_BO_2_ values lay at the peak of the curve as compared to the Pt, MoS_2_, ort-MBO, Ti_2_NO_2_, and Ti_2_CO_2_. These findings show that the MBenes possess an excellent HER performance. To produce CH_4_ selectively in practical uses, Guao et al. [24] presented MB2 MBene monolayers with high catalytic activity, Fe_2_B_4_, and Mn_2_B_4_ monolayers with shallow limiting potential for CH_4_ generation. Mo_2_B, a novel form of MBene material, has also demonstrated potential applications in LIBs due to its high thermal and electrical conductivity [196]. A form of Ti_2_B_2_ monolayer with electronic metallic characteristics and exceptional catalytic activity has also been used in HER [197]. The theoretical calculations show that MBene is a good candidate for energy harvesting. Zhang et al. [69] utilized the computational method to investigate the multifunctional HER reaction of the three MBenes, namely Ni_2_B_2_, Fe_3_B_4_, and Co_3_B_4_ MBenes. Their computational studies indicated the Ni_2_B_2_, Co_3_B_4_, and Fe_3_B_4_ MBenes are excellent, promising multifunctional HER and biofunctional HER electrocatalysts, respectively. The MBene ultralow low overpotentials of 0.26 and 0.30 V for OER and ORR reaction make it a better RuO_2_ catalyst. Most MBenes have outstanding inherent catalytic power, and their basal planes are also very effective at inhibiting interface oxidation, as measured by OER and ORR. The active regions are protected from O*/OH* by Fe_3_B_4_, Co_3_B_4_, and Ni_2_B_2_ MBenes. Additionally, the robust linear scaling connection between ΔG_OH*_ and the d-band center or charge migration assessment provides additional insight into the overpotentials of OER/ORR on MBene, which may be described by the variable ΔG_OH*_ that is available. The significantly reduced overpotentials of OER and ORR are made possible by the establishment of new scaling equations between the adsorption energies of OOH* and O* on MBenes, wherein ΔG_OOH*_ and ΔGO* possess distinct slopes versus ΔGOH*.

Zhang et al. [198] applied the DFT to calculate the HER electrocatalytic activity of the 2D chromium boride (Cr_n+1_B_2n_) MBenes. The DFT outcomes specified that the synthesized MBene-based electrocatalyst possessed good mechanical and ferromagnetic conductive structure and stability. They also investigated the impact of the thickness of the electrocatalysts on their mechanical and HER activity. The 2 × 2 supercell models were utilized to calculate the HER catalytic activity of Cr_n+1_B_2n_. The various H atom adsorption active sites were selected on the MBene-based catalyst to determine the effective sites of the adsorption of the MBene. They found that H adsorbed on the cr atom top and lower layer by comparing the H adsorption energy of the various selective sites of the MBene. The ΔG_H_ value of the Cr_2_B_2_, Cr_3_B_4,_ and Cr_4_B_6_ MBene was − 0.198, − 0.178, and − 0.078 eV, respectively; all MBene possessed good HER actively. Nevertheless, Cr_4_B_6_ has even more HER activity in all of them because of the low ΔG_H_ value. They also compare the MBene HER performance with MXene. The comparative analysis results show that MBene is good compared to the MXene as an electrocatalyst. In all of the considered 2D systems, Cr_4_B_6_ MBene performs better than Fe_2_B_2_. Cr_4_B_6_ shows nearly zero |Δ| GH at any taken H coverages.

Additionally, Cr_4_B_6_ has significantly higher HER catalytic activity than Pt when it has a complete layer of H atoms on its outermost layer. The catalytic efficiency increases with Cr_n+1_B_2n_ thicknesses. Consequently, the reason for the increased HER catalytic efficiency for thicker Cr_n+1_B_2n_ is that as the thickness of Cr_n+1_B_2n_ increases, the hydrogen adsorption becomes less powerful, resulting in a smaller ΔGH and less negative value of the hydrogen adsorption energy (ΔE_H_). The extremely –ve and + ve values of the ΔE_H_ are responsible for the low HER activity because these too much lower values possess any material in ideal conditions. The MBene is the best material for the HER and OER reactions because, in both situations, it gives the best theoretical performance compared to the practical working electrodes.

Shukla et al. [199] employed the DFT calculations to investigate the impact of the anchored MBene on the HER reaction performance of the single-atom catalysts (SACs) Pt. Their finding shows that the SACs anchored with MBene effectively and successfully improve the hydrogen adsorption with a volcano-like tendency, the Ni-Mo_2_B MBene possess optima ΔG_H*_ values for 1 T and 2H are − 0.003 and − 0.018 eV, respectively. The mechanism of the HER reaction over MBene is presented in Fig. 18g. Additionally, the SACs anchored with MBene have promising current densities up to 1.15 × 10^−3^ A cm^−2^; the obtained current density of the Pt/MBene is approximately ∼125% higher than most standard metal catalysts like Pt (111). The adsorption energy of the Hydrogen on the three different sites, T_H_, T_B,_ and T_Mo_ are − 0.959, − 0.914, and 0.432 eV for the pristine Mo_2_B MBene. The T_H_, T_B,_ and T_Mo_ represented the hollow, top, and Mo atom sites of the MBene for hydrogen adsorption. On the other hand, H more strongly interacts with the 1 T phase than the 2H phase for Mo_2_B, exhibiting intense adsorption energy to follow the trend TB < T_H_ < T_Mo_ is − 0.946 <  − 0.895 eV <  − 0.261 eV. It has been discovered that the T_Mo_ site is not very favorable for hydrogen adsorption in both phases. Based on the ΔG_H*_ values, the thermodynamics of the reactions are presented in Fig. 18h. The charge density difference and PDOS of H-adsorbed (T^Mo^ site) Mo_2_B are revealed in Fig. 18i-l. Now, we compared the HER performance of the Mo_2_B attached SACs. The MBene-based composite of the SACs such as Ir, Co, Ni, and Pt-based Mo2B composites ΔG_H*_ values are 0.031, 0.073, − 0.018, and − 0.036 eV, respectively, as presented in Fig. 18m. The MBene-based SACs composites possess very low ΔG_H*_ values compared to the pristine MBene, as demonstrated in Fig. 18h. Additionally, the PDOS of the MBene was calculated to evaluate the efficiency of the MBene-based composite materials, as specified in Fig. 18n-o. The most effective catalyst for the hydrogen evolution reaction among these systems is Ni-Mo_2_B. It shows a substantial orbital overlap between − 2 and − 6.1 eV, indicating an intense interaction between the hydrogen and nickel atoms, as seen in Fig. 18i, j. Nevertheless, the comparable energy levels of these two orbitals do not guarantee a robust bonding relationship between them. Thus, we do the expected COHP computations, as depicted in Fig. 18p, q. The COHP method divides the energy of the band’s configuration into orbital pair interactions, allowing us to determine the corresponding contributions of bonding and antibonding states for a specific bond based on the energy. In COHP diagrams, bonding (antibonding) phases are represented by a positive (negative) COHP with a positive value (Fig. 18p, q). The d and H s orbitals exhibit robust bonding features lower than the Fermi level. To impartially evaluate the bonding power, the ICOHP varies as − 1.143 eV for 2H and − 1.034 eV for 1TNi-Mo_2_B, respectively.

## Artificial Intelligence-based Opportunities in MBene Research

**Fig. 19:**
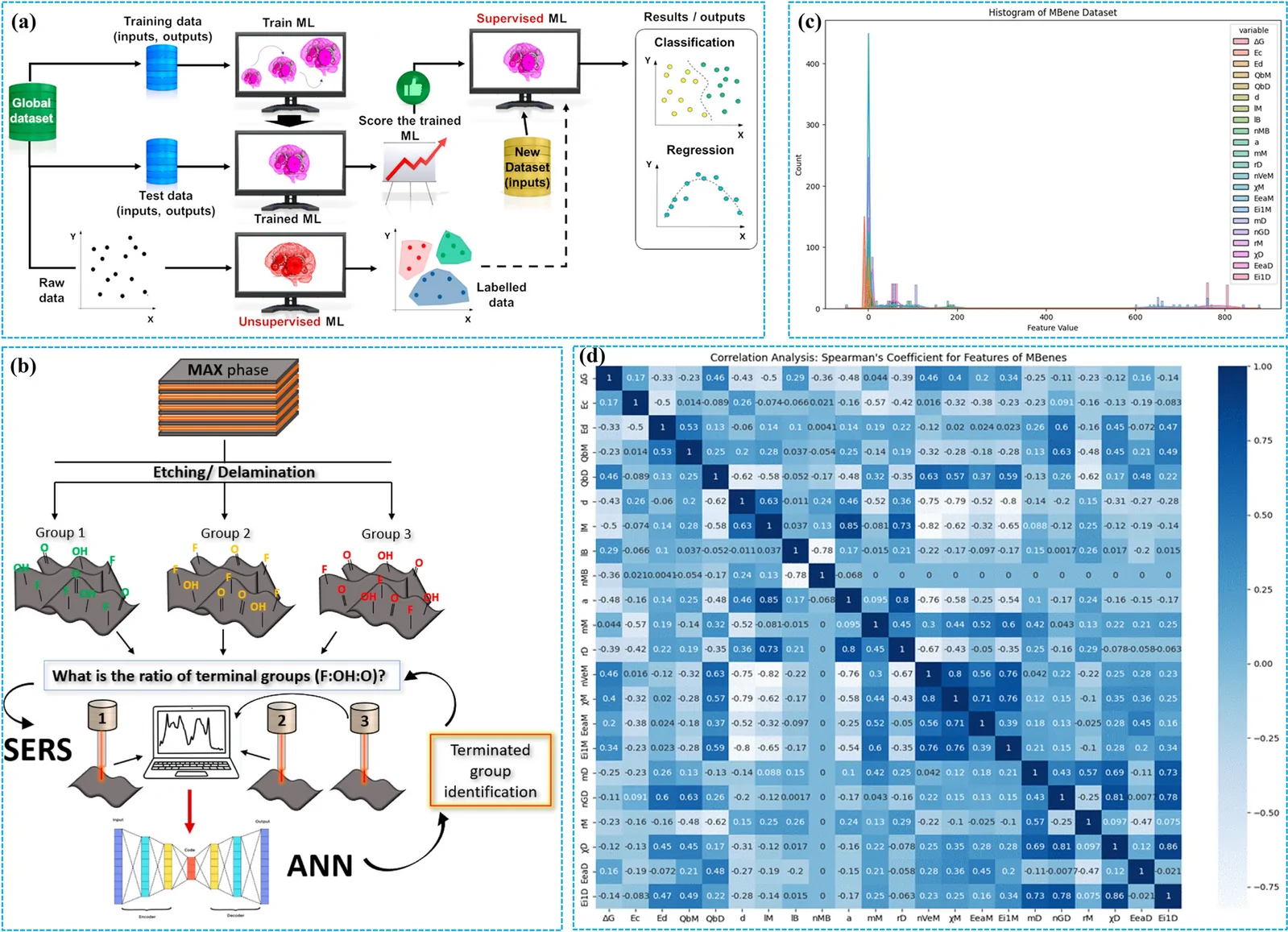
**a** Process of implementing machine learning (extracted with permission [201]). **b** Schematic illustration of the suggested experimental approach integrating SERS with ANN for the accurate and dependable assessment of the surface chemistry of MXene flakes (Ti_3_C_2_T_*x*_) (extracted with permission [202]). **c** An investigation of feature distribution in MBenes was performed using histogram evaluation. **d** Spearman’s correlation coefficient analysis was utilized to assess the correlation among various properties of MBenes (extracted with permission [203])

Although many studies offer significant insights into the potential of MBenes in energy applications, research that incorporates AI into the development or optimization of MBenes for energy storage and harvesting remains rare. The incorporation of AI in materials science is an expanding domain, and forthcoming study may investigate AI-facilitated design and optimization of MBenes to improve their efficacy in energy applications. 2024 may be the year of artificial intelligence. Nobel Prize in Physics laureates assert that artificial neural networks and machine learning have established the foundation for the advancement of AI [200]. The Nobel Prize in Chemistry was awarded to individuals who designed and forecasted protein structures. Currently, artificial intelligence in battery technology is in its early stages and has received inadequate research. Teo and coauthors analyzed artificial intelligence in battery technology [201]. The literature review indicates that AI influences various elements of battery growth (Fig. 19a). Artificial intelligence facilitates multiscale material modeling and enhances the evaluation and forecasting of solid, liquid, and active electrode materials for battery design. Multitarget machine learning is employed by Li et al. [201] to design MXenes for high-capacity energy storage. They use a new category descriptor to characterize the MXene chemical formula and forecast numerous electrochemical parameters. Subsequently, they reverse the design challenge and forecast MXenes formulas using battery performance requirements. This procedure uses multitarget regression and classification to focus on battery-relevant physicochemical characteristics. The final inverse model suggests Li_2_M_2_C and Mg_2_M_2_C (M: Sc, Ti, Cr) for further research due to their ideal gravimetric capacity, voltage, and induced charge. Recent research has focused on the potential integration of AI and machine learning ML into the analytical process of MBenes. Trelin et al. advocate for the integration of surface-enhanced Raman spectroscopy (SERS) and artificial neural networks (ANN) to accurately and reliably ascertain the surface chemistry of MXene flakes (Ti_3_C_2_T_*x*_). Ti_3_C_2_T_*x*_ flakes were separately synthesized by three research teams and subsequently analyzed using three distinct Raman spectrometers, utilizing resonance excitation frequencies [202]. The manual examination of the SERS spectra did not provide precise identification of the flake surface terminal. The integrated SERS-ANN methodology enabled us to ascertain the surface termination with considerable precision (Fig. 19b). The method’s dependability was confirmed by a series of independently manufactured samples. They meticulously examined the influence of flake stability and variations in flake processing and Raman measurement settings on the outcomes of the SERS-ANN approach. Consequently, they have devised a universal approach that is unaffected by the aforementioned criteria, yielding findings with precision comparable to XPS, while superior in analysis duration and ease of use. Two studies presented include a Particle Swarm Optimization-based analysis and stability assessment of Mbene materials using the Scikit-Learn Motivated Application Programming Interface: gplearn [203]. The stability of MBenes, characterized by the chemical formulas M_2_B_1_ and M_2_B_2_, has been examined with and without the doping of Pd and Co, where M represents a transition metal and B denotes boron. They accomplished this by employing gplearn, an application programming interface (API) influenced by scikit-learn. Upon first examination of the dataset, the researchers observed that it did not conform to a normal distribution. The Shapiro–Wilk test was conducted again on the entire dataset to confirm that the characteristics do not adhere to a normal distribution. Due to the absence of a bell-shaped distribution in the histogram of the complete dataset (illustrated in Fig. 19c) and the Shapiro–Wilk test yielding a normality significance threshold below 0.05, Spearman’s correlation coefficient was employed to find features with a correlation exceeding 90 percent. Figure 19d illustrates that the dataset lacked any such traits. These studies illustrate a growing interest in employing AI and ML for the analysis and enhancement of MBenes in energy storage applications. The prediction of novel MAB phases has been greatly accelerated by recent developments in ML, which has prepared the way for the investigation of MBene materials. Using ML techniques in conjunction with high-throughput DFT calculations and crystal structure prediction (CSP), scientists have discovered stable ternary borides [204]. These advancements make it easier to create novel MBene candidates, which have remarkable qualities like increased surface reactivity, metallic conductivity, and strong chemical stability [205]. Mo_4/3_B_2_ MBene, for example, has shown exceptional surface-enhanced Raman scattering (SERS) activity, with ultralow detection limits down to 10^–9^ M and a Raman enhancement factor of 3.88 × 10^6^ [206]. The promise of these 2D borides in next-generation sensing, energy storage, and environmental technologies is highlighted by the collaboration between MBene applications and ML-driven material discovery. As the field progresses, greater integration of AI is expected to accelerate the discovery and development of MBenes with enhanced properties for energy-related applications. Sharma and coauthors presented: designing possible energy surfaces for graphene-based 2D–3D interfaces from altered high-dimensional neural networks for uses in energy storage, illustrating the application of machine learning to model interfaces in heterostructure energy storage systems [207]. This research centers on graphene-based systems, although the approaches utilized may be adaptable for MBenes in subsequent investigations.

## Challenges and Opportunities in MXene Derivatives

As is the case with many other new materials, the development and implementation of MBenes are confronted with several problems that must be solved to realize their potential advantages fully. These are some of the most significant issues and possible remedies.

1. The production process of the MBene to eliminate the Al layer from their parent material is complex and costly because of the higher cost of the raw material and equipment. To solve the issue of the production process of MBene, a new, cost-effective, environmentally friendly, and advanced chemical vapor deposition (CVD) or bottom-up synthesizing technique must be developed. Many difficulties are involved in increasing production while preserving quality and consistency. The synthesis process should be automated and standardized according to industry standards to guarantee reproducibility and quality control and tackle the production and quality of the synthesized final MBene-based material. Generally, during the acid or alkali etching process, the concentration of the etching agent plays a vital role in enhancing the Al elimination concentration from the parent material of MBene. For example, Wang et al. [14] investigated the impact of the etching agent NaOH concentration on the elimination of the Al layer from MBene parent material enhanced by the concentration of the NaOH. Their experimental results show that synthesizing the hex-Ti_2_B_2_ from Ti_2_InB_2_ makes it purer by eliminating the ln layer in more quantities from their parent material by enhancing the concentration of the etching agent under mild conditions.
2. Although MBenes signify a swiftly evolving domain in two-dimensional materials, their practical implementation is hindered by obstacles in synthesis and application. Recent research has predominantly concentrated on post-etching alterations and stabilization techniques; nonetheless, the quality and characteristics of MBenes are fundamentally dictated by the structure and composition of their MAB phase precursors. The majority of MBenes are produced via selective etching of A elements from MAB phases, wherein the bonding characteristics and thermodynamic stability of these precursors critically affect the etching efficiency and the resultant 2D shape, crystallinity, and chemical functionality of MBenes. Consequently, future investigations should focus on refining etching techniques and the systematic design and identification of innovative MAB phase precursors. This may entail customizing the A layer chemistry, using metastable or alloyed MAB phases, or utilizing high-throughput computational screening to identify etchable candidates with advantageous M–A bonding properties. Moreover, investigating non-conventional or soft etching techniques that accommodate a wider array of precursors could extend the MBene family beyond existing constraints.
3. The long-term stability of MBenes is a significant problem, especially under ambient circumstances, due to their vulnerability to moisture and oxygen exposure deterioration. These environmental variables might cause the slow oxidation of virgin MBenes, hence degrading their fundamental characteristics and restricting their practical application [208]. A comparable issue is thoroughly documented for MXenes, whose oxidation in atmospheric and aqueous environments has been intensively investigated [209]. Indeed, MXenes in their aqueous dispersion demonstrate markedly accelerated oxidation relative to their freestanding sheet form [210]. The oxidative degradation of MBenes in aqueous conditions is under research; nevertheless, preliminary studies indicate similarities with Ti_3_C_2_T_*x*_-MXenes, where water is recognized as a primary catalyst for hydrolysis and degradation, with oxygen as an additional component. Multiple characteristics, including pH, flake size, concentration, and surface terminations, have been identified as influencing the oxidation behavior of MXenes [9]. Considering the structural and compositional parallels between MXenes and MBenes, it is plausible to deduce that MBenes may demonstrate inadequate stability for specific applications, particularly under oxidative or humid environments. Nonetheless, further systematic investigations are required to precisely evaluate and contrast their stability characteristics [20].
4. The composition and functionalization are effective techniques for altering the MBene’s energy storage and harvesting properties. The available methods are unsuitable for the effective and successful functionalization of the MBene. To increase the energy harvesting and storage capability of virgin MBene, scientists must discover a sophisticated and ecologically safe method to functionalize it. Hu et al. [211] carried out the DFT calculations to investigate the impact of the OH, O, Cl, and H functionalization on the performance of the pristine Cr_2_B_2_MBene. Still, limited experimental data on the functionalized and MBene-based composite in energy storage and harvesting is available. So, we need to develop new and advanced techniques to synthesize MBene-based composites and utilize their synergistic power to advance the performance and stability of the MBene.
5. The investigation of the MBene and MBene-based material is at an initial stage. Still, limited experimental studies are available on the structural and morphological behavior of the MBene. So, it is necessary to employ advanced characterization methods to comprehend the essential characteristics of MBenes at the atomic level. The structure and characteristics of MBenes are being investigated in great detail by applying advanced microscopy and spectroscopic methods. Additionally, the utilization of effective predictive models to explore the design and application of the MBene plays an essential role in an easy and detailed understanding of the structure of the MBene.
6. Despite showing great promise, the specific mechanism by which MBenes store and release energy is not always understood. Researchers are responsible for discovering these mechanisms to maximize their effectiveness. Even though these obstacles exist, researchers and scientists are working hard to find solutions to these problems. There is a possibility that MBenes materials will play an essential part in developing energy storage technologies in the coming years, as experts continue to investigate and better understand these materials.

## Conclusion

MBenes is gaining prominence because of its distinct architectural characteristics, intriguing activities, and considerable promise for energy storage and harvesting applications. Still, investigations into these innovative 2D materials are in their initial stages. MBenes have various opportunities and limitations because of the experimental stage’s unresolved difficulties and untapped potential. This paper examines the present issues related to empirical and theoretical studies. Multiple points of view are presented to provide solutions for tackling these difficulties. Moreover, this article offers an intricate depiction of potential research domains in MBenes. The fields of focus encompass the investigation of novel MBenes, the production of these substances, their possible uses, the study of phase-changing mechanisms, and the examination of hex-MBenes, orth-MBenes, tetra-MBene, tri-MBene, and MXenes with identical transition metal components. The objective is to encourage researchers to engage in this field of study.
